# An empirical comparison of Bayesian modelling strategies for missing binary outcome data in network meta-analysis

**DOI:** 10.1186/s12874-019-0731-y

**Published:** 2019-04-24

**Authors:** Loukia M. Spineli

**Affiliations:** Midwifery Research and Education Unit, Hannover Medical School, Carl-Neuberg-Str. 1, 30625 Hannover, Germany

**Keywords:** Missing data, Pattern-mixture model, Missing at random, Network meta-analysis, Systematic review

## Abstract

**Background:**

A number of strategies have been proposed to handle missing binary outcome data (MOD) in systematic reviews. However, none of these have been evaluated empirically in a series of published systematic reviews.

**Methods:**

Using published systematic reviews with network meta-analysis (NMA) from a wide range of health-related fields, we evaluated comparatively the most frequently described Bayesian modelling strategies for MOD in terms of log odds ratio (log OR), between-trial variance, inconsistency factor (i.e. difference between direct and indirect estimates for a comparison), surface under the cumulative ranking (SUCRA) and rankings. We extended the Bayesian random-effects NMA model to incorporate the informative missingness odds ratio (IMOR) parameter, and applied the node-splitting approach to investigate inconsistency locally. We considered both pattern-mixture and selection models, different structures for prior distribution of log IMOR, and different scenarios for MOD. To illustrate level of agreement between different strategies and scenarios, we used Bland-Altman plots.

**Results:**

Addressing MOD using extreme scenarios and ignoring the uncertainty about the scenarios led to systematically different and more precise log ORs compared to modelling MOD under the missing at random (MAR) assumption. Hierarchical structure of log IMORs led to lower between-trial variance, especially in the case of substantial MOD. Assuming common-within-network or trial-specific log IMORs yielded similar posterior results for all NMA estimates, whereas intervention-specific structure systematically inflated uncertainty around log ORs and SUCRAs. Pattern-mixture model agreed with selection model, particularly under the trial-specific structure; however, selection model systematically reduced precision around log IMORs. Overall, different strategies and scenarios mostly had good agreement in the case of low MOD.

**Conclusions:**

Addressing MOD using extreme scenarios and/or ignoring the uncertainty about the scenarios may negatively affect NMA estimates. Modelling MOD via the IMOR parameter can ensure bias-adjusted estimates and offer valuable insights into missingness mechanisms. The researcher should seek an expert opinion in order to decide on the structure of log IMOR that best aligns to the condition and interventions studied and to define a proper prior distribution for log IMOR. Our findings also apply to pairwise meta-analyses.

**Electronic supplementary material:**

The online version of this article (10.1186/s12874-019-0731-y) contains supplementary material, which is available to authorized users.

## Background

Missing (participant) outcome data (MOD) in a series of trials have preoccupied a number of researchers who have contributed to the development of several methods of different complexity (for example, [1–10]) to address primarily *binary* MOD in a pairwise meta-analysis. Only a handful of these methodologies have been extended further to operate in a network of several interventions [8, 11]. These methodological articles provide only limited empirical evidence to demonstrate the merits and demerits of proposed methods as they usually consider one published systematic review with pairwise or network meta-analyses (NMA). Furthermore, the modelling strategies and missingness scenarios considered to investigate the value of proposed methods differ considerably across methodological articles (Additional file 1: Table S1).

There is no universally ‘best’ strategy for how authors of systematic reviews should deal with MOD in included trials. Like other types of missing data (e.g. missing studies and outcomes), successful handling of MOD rests on plausible yet untestable assumptions regarding the missingness mechanism in conjunction with appropriate analytical strategies [4]. In practice, the missingness mechanism is explored by making sensible assumptions on whether data are informatively missing, and if so, what the outcomes would plausibly be if participants had never left the trial. When included trials provide limited or no information on the reasons for MOD, in order to explore assumptions empirically, the meta-analyst examines the sensitivity of results to plausible scenarios [2]. A usual starting point of the analysis is to assume that data are missing at random (MAR) and then investigate any deviations from this assumption by performing a series of sensitivity analyses (Additional file 1: Table S1) [2–4, 12].

According to the Cochrane handbook (version 5.1.0) [13], principal options to deal with MOD in a pairwise meta-analysis constitute (i) exclusion of missing participants from the analysis, (ii) imputation of missing outcomes in each arm of every trial using specific scenarios and (iii) statistical modelling of the missingness mechanism. Furthermore, uncertainty induced by imputing MOD according to item (ii) might be accounted for or not in the meta-analysis results [1, 2]. These options are also relevant in the context of NMA. Since NMA is an extension of pairwise meta-analysis, these options extend naturally even though authors of relevant published literature may not have explicitly done (e.g. Turner et al. [10]). However, extension of these options to a network of interventions should be accompanied by comprehensive investigation and acknowledgement of the implications of MOD on core components of the NMA model (i.e. consistency equation and ranking measures). Otherwise, a suboptimal reporting and handling of MOD in a network of interventions can greatly raise risk of providing misleading conclusions.

We consider statistical modelling to be a more proper strategy to handle MOD because – contrary to exclusion or imputation of MOD before analysis – it accounts for possible bias and uncertainty around trial-specific estimates of treatment effect due to MOD while maintaining the randomised sample in each trial [10]. In particular, modelling MOD using Bayesian approaches – the latter being very popular in NMA as they foster probabilistic statements that are an integral part of the inferential NMA framework [14, 15] – naturally allows for uncertainty induced by MOD to be incorporated into NMA estimates using proper prior distributions. To explore the implications of different Bayesian modelling strategies of binary MOD on core NMA components, we set up a comprehensive empirical study using published systematic reviews with NMA from a wide range of health-related fields [16]. In this way, we can investigate whether, and for which NMA estimates, the compared modelling strategies disagree using real data and taking into account the extent and balance of MOD within each network – factors that may trigger this discordance. Since NMA constitutes an increasingly applied evidence-synthesis tool that has become widely acknowledged by researchers and policy-making bodies, such as the National Institute of Clinical Excellence [15, 17–19], it is crucial to provide the necessary, empirically based directions to handle MOD appropriately in a network of several interventions.

The rest of the article is organised as follows. Initially, we describe our analysed dataset and then review the modelling strategies and missingness scenarios that we incorporated in the Bayesian random-effects NMA model. Furthermore, we delineate the analyses we performed to compare the reviewed modelling strategies in terms of NMA estimates. Then, we present the results of the empirical evaluation, we discuss our results and highlight important limitations and recommendations, and we provide our conclusions.

## Methods

### Selection process of analysed dataset

This empirical study was based on our previous survey with systematic reviews of multiple interventions published between 01/01/2009 and 31/03/2017 in peer-reviewed journals of several health-related fields [16]. Details on the search strategy and selection process of the eligible systematic reviews and NMAs can be found in our previous work [16].

We only considered NMAs (31 in total) that provided arm-level binary outcome data with present MOD in included trials; however, we excluded one review where no NMA was employed, and one review for reporting data in a non-extractable manner. The whole selection process resulted in 29 eligible NMAs in total that comprised our empirical dataset.

We used odds ratio (OR) as the effect measure in all eligible NMAs mainly due to its preferred statistical properties [20]. In each network, we recorded outcome events so that OR more than 1 indicated beneficial effect for the first intervention in each comparison.

### Characterising networks based on prevalence and balance of MOD

We considered the ‘five-and-twenty rule’ as proposed by Sackett et al. [21] to determine a trial as having low (MOD ≤5%), moderate and large risk (MOD > 20%) of attrition bias. Furthermore, we calculated difference in percentage of MOD (%MOD) between compared interventions in order to define MOD as being balanced or unbalanced in each trial of every network. By applying these rules, we distinguished networks with ‘low’, ‘moderate and balance’, ‘moderate and imbalance’, ‘large and balance’ and ‘large and imbalance’ MOD. Step-by-step details on this strategy can be found in the web appendix (Additional file 2).

### Missingness models in network meta-analysis

In the presence of MOD, we need a model that incorporates both the missing and observed information and, in addition, allows us to learn about missingness mechanisms. We briefly describe two missingness models that have been proposed for that purpose.

#### Pattern-mixture model

Consider a network of *N* trials investigating different sets of *T* interventions. In arm *k* = 1, 2, … , *a*_*i*_ of trial *i*, we observe the number of events, *r*_*ik*_, and the number of MOD, *m*_*ik*_, out of the total randomised, *n*_*ik*_. In arm *k* of trial *i*, the number of observed events and the number of MOD are assumed to be sampled from the corresponding binomial distributions [10]:

$$ {r}_{ik}\sim Bin\left({p}_{ik}^o,{n}_{ik}-{m}_{ik}\right)\ \mathrm{and}\ {m}_{ik}\sim Bin\left({q}_{ik},{n}_{ik}\right) $$

with ${p}_{ik}^o$ being the probability of event conditional on the completers and *q*_*ik*_ being the probability of MOD.

The pattern-mixture model was the most commonly described model to address MOD in systematic reviews (Additional file 1: Table S1). It describes distribution of the outcome between completers and missing participants [3, 10]. Then, the underlying probability of event in arm *k* of trial *i*, *p*_*ik*_, is modelled conditional on whether an event is observed or missing [10]:

$$ {p}_{ik}={p}_{ik}^o\bullet \left(1-{q}_{ik}\right)+{p}_{ik}^m\bullet {q}_{ik} $$

where ${p}_{ik}^m$ indicates the probability of event conditional on missing participants in arm *k* of trial *i*. Following Turner et al. [10], the above equation can be re-arranged to link ${p}_{ik}^o$ with the remaining parameters:

1 $$ {p}_{ik}^o=\frac{p_{ik}-{p}_{ik}^m\bullet {q}_{ik}}{1-{q}_{ik}} $$

Then, using the logit function, we define the log odds of event in arm *k* of trial *i* as follows:

2 $$ logit\left({p}_{ik}\right)={u}_i+{\theta}_{i,k1}\bullet I\left(k>1\right) $$

where *u*_*i*_ = *logit*(*p*_*i*1_) is the log odds of event in the baseline arm of trial *i* and *θ*_*i*, *k*1_ is the log OR of event in arm *k* relative to the baseline arm of trial *i*. Typically, *θ*_*i*, *k*1_ follows a normal distribution with mean ${\mu}_{t_{ik}{t}_{i1}}$ (i.e. the summary log OR of event between intervention *t*_*ik*_ and *t*_*i*1_ of trial *i*) and variance *τ*^2^, which is commonly assumed to be constant across different comparisons. The index *t*_*ik*_ indicates the intervention studied in arm *k* of trial *i*. In trial *i* with *a*_*i*_ ≥ 3 arms, log ORs are correlated since they share the same comparator and therefore follow a multivariate normal distribution, which is equivalent to conditional univariate normal distributions for *θ*_*i*, *k*1_ of arm *k* > 2, conditional on all arms from *k* = 2 to *a*_*i*_ − 1 (eq. 11 in Dias et al. [22]).

Under the consistency assumption (which implies statistical agreement between direct and (possibly more than one) indirect sources of evidence [14]), summary log ORs for all possible comparisons among non-reference interventions are obtained as functions of *T* − 1 summary log ORs for the basic parameters, namely, treatment effects relative to the reference intervention of the network (here, the reference is intervention 1):

3 $$ {\mu}_{tl}={\mu}_{t1}-{\mu}_{l1} $$

with *t*, *l* = {2, 3,  … , *T*} and *t* ≠ *l*.

#### Selection model

Another way to model observed data (i.e. *r*_*ik*_, *n*_*ik*_ − *r*_*ik*_ − *m*_*ik*_ and *m*_*ik*_) is to consider the following multinomial distribution [4, 11] in arm *k* of trial *i*:

$$ {\left({r}_{ik},{n}_{ik}-{r}_{ik}-{m}_{ik},{m}_{ik}\right)}^T\sim M\left({p}_{1, ik},{p}_{2, ik,},{p}_{3, ik},{n}_{ik}\right) $$

with

$$ {\displaystyle \begin{array}{c}{p}_{1, ik}=\left(1-{c}_{1, ik}\right)\bullet {p}_{ik}\\ {}{p}_{2, ik}=\left(1-{c}_{0, ik}\right)\bullet \left(1-{p}_{ik}\right)\\ {}{p}_{3, ik}={c}_{1, ik}\bullet {p}_{ik}+{c}_{0, ik}\bullet \left(1-{p}_{ik}\right)\end{array}} $$

where *p*_1, *ik*_ reflects the marginal probability of observing the underlying event, *p*_2, *ik*_ reflects the marginal probability of not observing the underlying event and *p*_3, *ik*_ is actually the probability of MOD in arm *k* of trial *i* (i.e. *p*_3, *ik*_ = *q*_*ik*_) and is modelled conditional on whether the missing participants may have experienced the underlying event or not [4, 11]. The last line describes the selection model [4, 11]. Then, parameters *c*_1, *ik*_ and *c*_0, *ik*_ denote the probability of MOD conditional on those participants with the underlying event and the probability of MOD conditional on those participants without the underlying event in arm *k* of trial *i*, respectively. Only *q*_*ik*_ is estimable from the data, and thus, we need to assign proper prior distributions on all other parameters.

### Informative missingness odds ratio parameter

To be able to incorporate plausible informative prior beliefs about the missingness process, we need alternative missingness parameters to ${p}_{ik}^m$*, c*_1, *ik*_ and *c*_0, *ik*_ that measure the relationship between the underlying outcome (event or non-event) and the status of the outcome (being missing or observed) [10]. Alternative missingness parameters have been already proposed in the literature.

Informative missingness odds ratio (IMOR) appeared to be the most popular missingness parameter in the literature (Additional file 1: Table S1). Under the pattern-mixture model, it is defined as the ratio of the odds of an event conditional on missing participants to the odds of an event conditional on completers in arm *k* of trial *i* [2, 3, 10]:

$$ {IMOR}_{ik}={\varphi}_{ik}=\frac{p_{ik}^m/\left(1-{p}_{ik}^m\right)}{p_{ik}^o/\left(1-{p}_{ik}^o\right)}. $$

Then, Eq. (1) can be re-written as follows (see also Appendix A in Turner et al. [10]):

$$ {p}_{ik}^o=\frac{-\left(\left({q}_{ik}-{p}_{ik}\right)\left(1-{\varphi}_{ik}\right)-1\right)-\sqrt{{\left(\left({q}_{ik}-{p}_{ik}\right)\left(1-{\varphi}_{ik}\right)-1\right)}^2-4{p}_{ik}\left(1-{q}_{ik}\right)\left(1-{\varphi}_{ik}\right)}}{2\left(1-{q}_{ik}\right)\left(1-{\varphi}_{ik}\right)} $$

Under the selection model, IMOR is defined as the ratio of the odds of MOD conditional on those with the underlying event to the odds of MOD conditional on those participants without the underlying event in arm *k* of trial *i* [4, 11]:

$$ {\varphi}_{ik}=\frac{c_{1, ik}/\left(1-{c}_{1, ik}\right)}{c_{0, ik}/\left(1-{c}_{0, ik}\right)} $$

Then, *c*_1, *ik*_ and *c*_0, *ik*_ can be parameterised with regard to *φ*_*ik*_ in the logarithmic scale (i.e. *log*(*φ*_*ik*_) = *δ*_*ik*_) and parameter *γ*_*ik*_ that indicates the average MOD across underlying event and underlying non-event in arm *k* of trial *i* as follows [4, 11]:

$$ {\displaystyle \begin{array}{c} logit\left({c}_{1, ik}\right)={\gamma}_{ik}+{\delta}_{ik}/2\\ {} logit\left({c}_{0, ik}\right)={\gamma}_{ik}-{\delta}_{ik}/2\end{array}} $$

with

$$ {\gamma}_{ik}=\frac{logit\left({c}_{1, ik}\right)+ logit\left({c}_{0, ik}\right)}{2} $$

In both missingness models, IMOR takes positive values, with IMOR equals 1 being equivalent to MAR. Then, in both missingness models, we use equations (2) and (3) with a random-effects model for *θ*_*i*, *k*1_ to apply random-effects NMA model with consistency equations.

Similar to OR, IMOR is applied in the logarithmic scale but is back-transformed in order to aid interpretation. Then, a natural choice is to apply a normal prior distribution on *δ*_*ik*_:

$$ {\delta}_{ik}\sim N\left({\varDelta}_{ik},{\sigma}_{ik}^2\right) $$

where *Δ*_*ik*_ is the average belief about the missingness scenario in arm *k* of trial *i* and ${\sigma}_{ik}^2$ is the uncertainty about this belief.

Other alternative missingness parameters that have been proposed are the event probability ratio within a pattern-mixture model by Akl et al. [6], and the response probability ratio within a selection model by Magder [23]. Being ratios of risks, these missingness parameters are more likely to be used alongside the relative risk ratio as outcome measure. Turner et al. [10] also reported these missingness parameters in the context of a Bayesian framework. In the present study, we preferred IMOR to the aforementioned alternative missingness parameters for being intuitively related to OR and for sharing the same statistical properties with OR (i.e. symmetry and prediction of event rates within [0, 1]) [2].

### Identical and hierarchical structure of normal prior distribution for ***δ***_***ik***_

Identical structure was the preferred prior structure in the majority of methodological articles (Additional file 1: Table S1) and is the simplest assumption as it yields the least parameters to estimate. Under this structure, *δ*_*ik*_ is considered identical depending on further assumptions that relate to whether missingness mechanisms may be common in the whole network:

$$ {\delta}_{ik}=\delta, \delta \sim N\left(\Delta , {\sigma}^2\right) with\ {\Delta }_{ik}=\Delta \ and\ {\sigma}_{ik}^2={\sigma}^2, $$

trial-related:

$$ {\delta}_{ik}={\delta}_i{\delta}_i\sim N\left({\varDelta}_i,{\sigma}_i^2\right)\mathrm{with}{\Delta}_{ik}={\Delta}_i and{\sigma}_{ik}^2={\sigma}_i^2, $$

or intervention-related:

$$ {\delta}_{ik}={\delta}_{t_{ik}},{\delta}_{t_{ik}}\sim N\left({\Delta}_{t_{ik}},{\sigma}_{t_{ik}}^2\right) with{\Delta}_{ik}={\Delta}_{t_{ik}} and{\sigma}_{ik}^2={\sigma}_{t_{ik}}^2 $$

In the present study, we considered *σ*^2^, ${\sigma}_i^2$ and ${\sigma}_{t_{ik}}^2$ to be the same: ${\sigma}^2={\sigma}_i^2={\sigma}_{t_{ik}}^2$.

Hierarchical structure assumes that *δ*_*ik*_ s are different yet related to each other by allowing for ‘information to be borrowed’ that is common-within-network:

*δ*_*ik*_~*N*(*Δ*, *σ*^2^) with *Δ*~*N*(*ξ*, *ψ*^2^), *σ*~*U*(0, *ψ*),

trial-specific (i.e. across different interventions in the same trial):

$$ {\delta}_{ik}\sim N\left({\varDelta}_i,{\sigma}_i^2\right) $$ with $$ {\varDelta}_i\sim N\left({\xi}_i,{\psi}_i^2\right) $$ and *σ*_*i*_~*U*(0, *ψ*_*i*_),

or intervention-specific (i.e. across different trials for the same intervention):

$$ {\delta}_{ik}\sim N\left({\varDelta}_{t_{ik}},{\sigma}_{t_{ik}}^2\right) $$ with $$ {\varDelta}_{t_{ik}}\sim N\left({\xi}_{t_{ik}},{\psi}_{t_{ik}}^2\right) $$, $$ {\sigma}_{t_{ik}}\sim U\left(0,{\psi}_{t_{ik}}\right). $$

with *ξ*, *ξ*_*i*_ and ${\xi}_{t_{ik}}$ being the mean of the hyper-parameters *Δ*, *Δ*_*i*_ and ${\varDelta}_{t_{ik}}$, respectively, and *ψ*^2^, ${\psi}_i^2$ and ${\psi}_{t_{ik}}^2$ being the corresponding variances. In the present study, we considered *ψ*^2^, ${\psi}_i^2$ and ${\psi}_{t_{ik}}^2$ to be the same: ${\psi}^2={\psi}_i^2={\psi}_{t_{ik}}^2$. We assigned a uniform distribution on *σ*, *σ*_*i*_ and ${\sigma}_{t_{ik}}$; however, researchers may consider other appropriate prior distributions for variance components [24]. Turner et al. [10] also briefly presented the independent structure, which is the least strong assumption to consider but yields the most parameters to estimate; however, in the present study, we did not consider the independent structure.

### Missingness scenarios using ***δ***_***ik***_

On average MAR (i.e. $\varDelta ={\varDelta}_i={\varDelta}_{t_{ik}}=0$ and $\xi ={\xi}_i={\xi}_{t_{ik}}=0$ under identical and hierarchical structure, respectively) with moderate prior variance of *δ*_*ik*_ (i.e. *σ*^2^ = 1 and *ψ*^2^ = 1 under identical and hierarchical structure, respectively) was the principal scenario in the present study. In addition, we considered the following extreme scenarios for identical structure only and we applied them under the pattern-mixture model (again with *σ*^2^ = 1):

- ${e}^{\varDelta_{t_{ik}}}=2$: the odds of an event in missing participants is twice the odds of an event in completers across all interventions – we call this scenario ‘more missing cases are events (MME)’;
- ${e}^{\varDelta_{t_{ik}}}=1/2$: the odds of an event in completers is twice the odds of an event in missing participants across all interventions – we call this scenario ‘more missing cases are non-events (MMNE);
- the odds of an event in missing participants is twice the odds of an event in completers in all non-reference interventions of the network (i.e. ${e}^{\varDelta_{t_{ik}}}=2$ for *t*_*ik*_ ≠ 1 with 1 being the reference of the network), whereas the opposite holds for the reference intervention (i.e. ${e}^{\varDelta_1}=1/2$ with 1 being the reference of the network) – we call this scenario ‘more missing cases are events for the non-reference interventions of the network’ (best-case scenario (BC) for the non-reference interventions); and
- the odds of an event in completers is twice the odds of an event in missing participants in all non-reference interventions of the network (i.e. ${e}^{\varDelta_{t_{ik}}}=1/2$ for *t*_*ik*_ ≠ 1 with 1 being the reference of the network), whereas the opposite holds for the reference intervention (i.e. ${e}^{\varDelta_1}=2$ with 1 being the reference of the network) – we call this scenario ‘more missing cases are non-events for the non-reference interventions of the network’ (worst-case scenario (WC) for the non-reference interventions).

Ideally, ${\varDelta}_{t_{ik}}\ne 0$ should be defined based on expert judgment tailored to the condition and interventions studied; however, we used the values we applied in our previous work [11].

### Research questions investigated

We re-analysed all 29 networks while considering the aforementioned missingness models and structures of normal prior distribution for *δ*_*ik*_ in order to investigate, initially (i) whether there is agreement between on average MAR and extreme scenarios (analysis A1); and (ii) whether there is agreement between accountability and ignorance of uncertainty due to MOD under MAR and extreme scenarios (analysis A2). Then, we evaluated (i) whether there is agreement between identical and hierarchical prior structure for *δ*_*ik*_ while considering *δ*_*ik*_ to be common-within-network, trial- and intervention-specific (analysis B1); (ii) whether there is agreement among further structural assumptions (i.e. common-within-network, trial- and intervention-specific) when *δ*_*ik*_ has identical prior structure (analysis B2a) and when *δ*_*ik*_ has hierarchical prior structure (analysis B2b); and (iii) whether there is agreement between pattern-mixture and selection model while considering *δ*_*ik*_ to be common-within-network, trial- and intervention-specific (analysis B3). Lastly, as an additional analysis, we investigated whether moderate prior variance of *δ*_*ik*_ (*σ*^2^ = 1 applied in all aforementioned analyses) agrees with conservative (*σ*^2^ = 4) and liberal (*σ*^2^ = 0.25) prior variance of *δ*_*ik*_ (analysis C1) – the latter carries more information about the missingness mechanism. These prior variance values for *δ*_*ik*_ have been recommended by White et al. [3, 4]. Details on missingness models, structures of *δ*_*ik*_ and missingness scenarios considered in each analysis can be found in Table 1.

**Table 1:** Research questions investigated and description of applied missingness models and prior structures for *δ*_*ik*_

Analysis	MOD model	Determination of *δ*_*ik*_				What is compared?
		Structure	Structural assumption	Scenario	Prior specification	
A1	pattern-mixture	identical	intervention-specific	MAR on average	${\delta}_{ik}={\delta}_{t_{ik}}$, ${\delta}_{t_{ik}}\sim N\left(0,1\right)$	MAR on average is compared with extreme scenarios (on average). Intervention 1 is the reference of the network.
				MME on average	${\delta}_{ik}={\delta}_{t_{ik}}$, ${\delta}_{t_{ik}}\sim N\left(\mathit{\ln}(2),1\right)$	
				MMNE on average	${\delta}_{ik}={\delta}_{t_{ik}}$, ${\delta}_{t_{ik}}\sim N\left(-\mathit{\ln}(2),1\right)$	
				BC on average	${\delta}_{ik}={\delta}_{t_{ik}}$, ${\delta}_{t_{ik}}\sim N\left(\mathit{\ln}(2),1\right)$, *t*_*ik*_ ≠ 1 ${\delta}_{ik}={\delta}_{t_{ik}}$, ${\delta}_{t_{ik}}\sim N\left(-\mathit{\ln}(2),1\right)$, *t*_*ik*_ = 1	
				WC on average	${\delta}_{ik}={\delta}_{t_{ik}}$, ${\delta}_{t_{ik}}\sim N\left(-\mathit{\ln}(2),1\right)$, *t*_*ik*_ ≠ 1 ${\delta}_{ik}={\delta}_{t_{ik}}$, ${\delta}_{t_{ik}}\sim N\left(\mathit{\ln}(2),1\right)$, *t*_*ik*_ = 1	
A2	pattern-mixture	identical	intervention-specific	MAR fixed	${\delta}_{ik}={\delta}_{t_{ik}}$, ${\delta}_{t_{ik}}=0$	‘On average’ scenarios are compared with corresponding ‘fixed’ scenarios. Intervention 1 is the reference of the network.
				MME fixed	${\delta}_{ik}={\delta}_{t_{ik}}$, ${\delta}_{t_{ik}}=\mathit{\ln}(2)$	
				MMNE fixed	${\delta}_{ik}={\delta}_{t_{ik}}$, ${\delta}_{t_{ik}}=-\mathit{\ln}(2)$	
				BC fixed	${\delta}_{ik}={\delta}_{t_{ik}}$, ${\delta}_{t_{ik}}=\mathit{\ln}(2)$, *t*_*ik*_ ≠ 1 ${\delta}_{ik}={\delta}_{t_{ik}}$, ${\delta}_{t_{ik}}=-\mathit{\ln}(2)$, *t*_*ik*_ = 1	
				WC fixed	${\delta}_{ik}={\delta}_{t_{ik}}$, ${\delta}_{t_{ik}}=-\mathit{\ln}(2)$, *t*_*ik*_ ≠ 1 ${\delta}_{ik}={\delta}_{t_{ik}}$, ${\delta}_{t_{ik}}=\mathit{\ln}(2)$, *t*_*ik*_ = 1	
B1	pattern-mixture	identical	common-within-network	MAR on average	*δ*_*ik*_ = *δ*, *δ*~*N*(0, 1)	Identical is compared with hierarchical for each structural assumption.
			trial-specific		*δ*_*ik*_ = *δ*_*i*_, *δ*_*i*_~*N*(0, 1)	
			intervention-specific		${\delta}_{ik}={\delta}_{t_{ik}}$, ${\delta}_{t_{ik}}\sim N\left(0,1\right)$	
		hierarchical	common-within-network	MAR on average	*δ*_*ik*_~*N*(*Δ*, *σ*^2^), *Δ*~*N*(0, 1), *σ*~*U*(0, 1)	
			trial-specific		${\delta}_{ik}\sim N\left({\varDelta}_i,{\sigma}_i^2\right)$, *Δ*_*i*_~*N*(0, 1), *σ*_*i*_~*U*(0, 1)	
			intervention-specific		${\delta}_{ik}\sim N\left({\varDelta}_{t_{ik}},{\sigma}_{t_{ik}}^2\right)$, ${\varDelta}_{t_{ik}}\sim N\left(0,1\right)$, ${\sigma}_{t_{ik}}\sim U\left(0,1\right)$	
B2a	pattern-mixture	identical	as in analysis B1	MAR on average	as in analysis B1	Structural assumptions are compared with each other.
B2b	pattern-mixture	hierarchical	as in analysis B1	MAR on average	as in analysis B1	Structural assumptions are compared with each other.
B3	pattern-mixture	identical	intervention-specific	MAR on average	${\delta}_{ik}={\delta}_{t_{ik}}$, ${\delta}_{t_{ik}}\sim N\left(0,1\right)$	Pattern-mixture model is compared with selection model for each structural assumption.
	selection					
C1	pattern-mixture	identical	intervention-specific	MAR on average	${\delta}_{ik}={\delta}_{t_{ik}}$, ${\delta}_{t_{ik}}\sim N\left(0,1\right)$ – moderate ${\delta}_{ik}={\delta}_{t_{ik}}$, ${\delta}_{t_{ik}}\sim N\left(\mathrm{0,0.25}\right)$ – liberal ${\delta}_{ik}={\delta}_{t_{ik}}$, ${\delta}_{t_{ik}}\sim N\left(0,4\right)$ – conservative	Moderate prior variance for *δ*_*ik*_ is compared with liberal and conservative prior variance.

Abbreviations: *BC* Best-case scenario, *MAR* Missing at random, *MME* More missing cases are events, *MMNE* More missing cases are non-events, *MOD* Missing outcome data, WC, worst-case scenario

### Network estimates and measure of disagreement

We obtained posterior distribution of log ORs for the basic parameters, *τ*^2^ s, inconsistency factors (IF; difference between direct and indirect estimates for a comparison in a closed loop, that is, a polygon that connects three or more interventions [14]) through the node-splitting approach [25, 26], SUCRAs (surface under the cumulative ranking) and posterior median rankings for all studied interventions [27]. A brief explanation of node-splitting approach and SUCRAs can be found in Additional file 2. For each analysis, we measured disagreement in compared methods in terms of NMA estimates using difference in posterior mean of log ORs, IFs, SUCRAs, and ratio of posterior median of *τ*^2^ s. Furthermore, we measured disagreement in compared methods in terms of uncertainty around NMA estimates using ratio of posterior standard deviation of log ORs, *τ*^2^ s, IFs, and difference in posterior standard deviation of SUCRAs. Moreover, we measured disagreement in compared methods in terms of *δ*_*ik*_ (analyses B1, B3, and C1) using differences in posterior mean and ratio of posterior standard deviation under the corresponding structural assumptions (Table 1).

### Presentation of results using Bland-Altman plots and Cohen’s kappa statistic

We used Bland-Altman plots to investigate level of agreement in all analyses [28]. In each Bland-Altman plot, we displayed average bias (i.e. mean of the differences or exponential of the mean of log ratios) and 95% limits of agreement (LoA; average bias as mean of differences or log ratios ± 1.96 ∙*s*_*D*_, that is, the standard deviation of differences or log ratios, respectively) [28]. We decided in advance to consider compared methods as having good agreement when average bias was close to 0 (for differences) or 1 (for ratios) and most of the points were uniformly scattered within the LoA – the narrower the LoA, the better the agreement. Agreement in terms of posterior median of rankings was investigated using heat-maps.

Furthermore, in each analysis, we compared strength and direction of evidence in posterior mean of log ORs and posterior mean of IFs. For that purpose, we applied Cohen’s kappa statistic (a coefficient that measures the inter-rater agreement for nominal items) [29] and we presented the estimated statistic alongside its 95% confidence interval. We used the divisions of agreement reported in Landis and Koch [30] in order to interpret this statistic. In a similar way, we worked with the extent of *τ*^2^ in each network, where we considered empirical distributions tailored to studied outcome and intervention-comparison type per network in order to determine posterior median of *τ*^2^ as low (less than the median of empirical distribution), moderate (between median and 3rd quartile) and large (above 3rd quartile) [31].

### Model specification

All NMA models were fitted using JAGS via the R package *R2jags* [32] (statistical software R, version 3.3.1 [33]), whereas the node-splitting model was performed using the R package *gemtc* [25, 34] in conjunction with the node-splitting model of Dias et al. [26]. Further information on specification of the NMA models and node-splitting approach (e.g. prior distributions assigned and diagnostic evaluation of convergence) can be found in the web appendix (Additional file 2). The codes to run all NMA models in JAGS can be found in Additional file 3, whereas the analysed dataset can be found in Additional file 4. We produced self-created Bland-Altman plots using the R packages *ggplot2* and *cowplot* [35, 36].

## Results

### Distribution of MOD across health-related fields

Out of 29 NMAs, 14 (48%) were judged to have ‘moderate and balance’ MOD, followed by 12 (41%) with ‘low’ MOD, two with ‘moderate and unbalanced’ MOD, and one with ‘large and unbalanced’ MOD (Additional file 1: Tables S2 and S3). No network fell into the ‘large and balance’ MOD category.

**Fig. 1:**
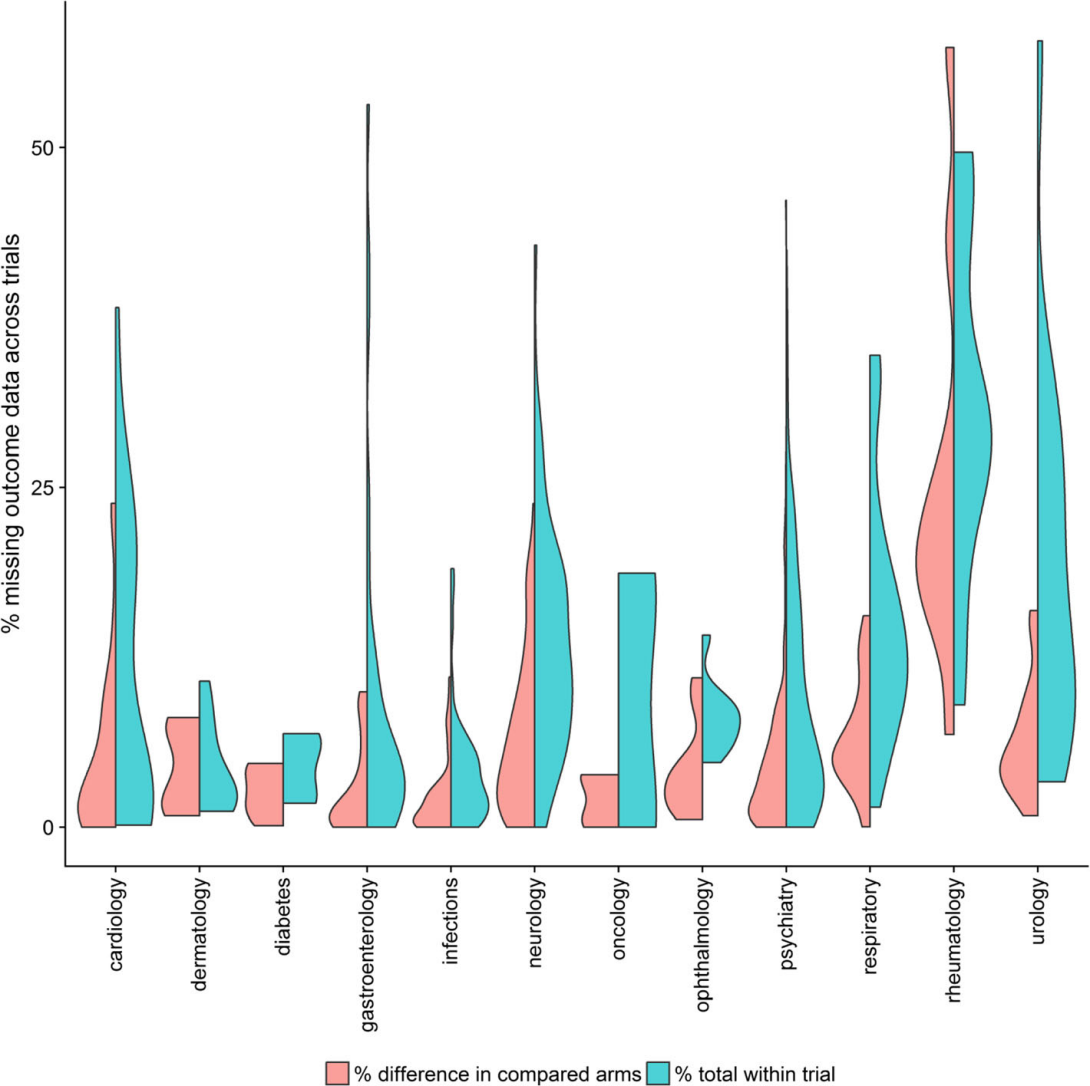
Split violin plots grouped by health specialty. Red violins illustrate density of differences in percentage missing outcome data between compared intervention arms across trials of all networks, whereas blue violins indicate distribution of total percentage missing outcome data across trials of all networks

Overall, there was great dispersion of total %MOD (blue violin plots) across trials in all health-related fields (Fig. 1). In comparison with dermatology, diabetes, infections and ophthalmology, total %MOD for the remaining health-related fields were distributed across a greater range – most of them exceeding 10%. On the contrary, differences in %MOD between compared arms (red violin plots) were relatively less dispersed across health-related fields, except for cardiology, neurology, respiratory, rheumatology and urology (Fig. 1).

### Implications of extreme scenarios about the missingness mechanism

**Fig. 2:**
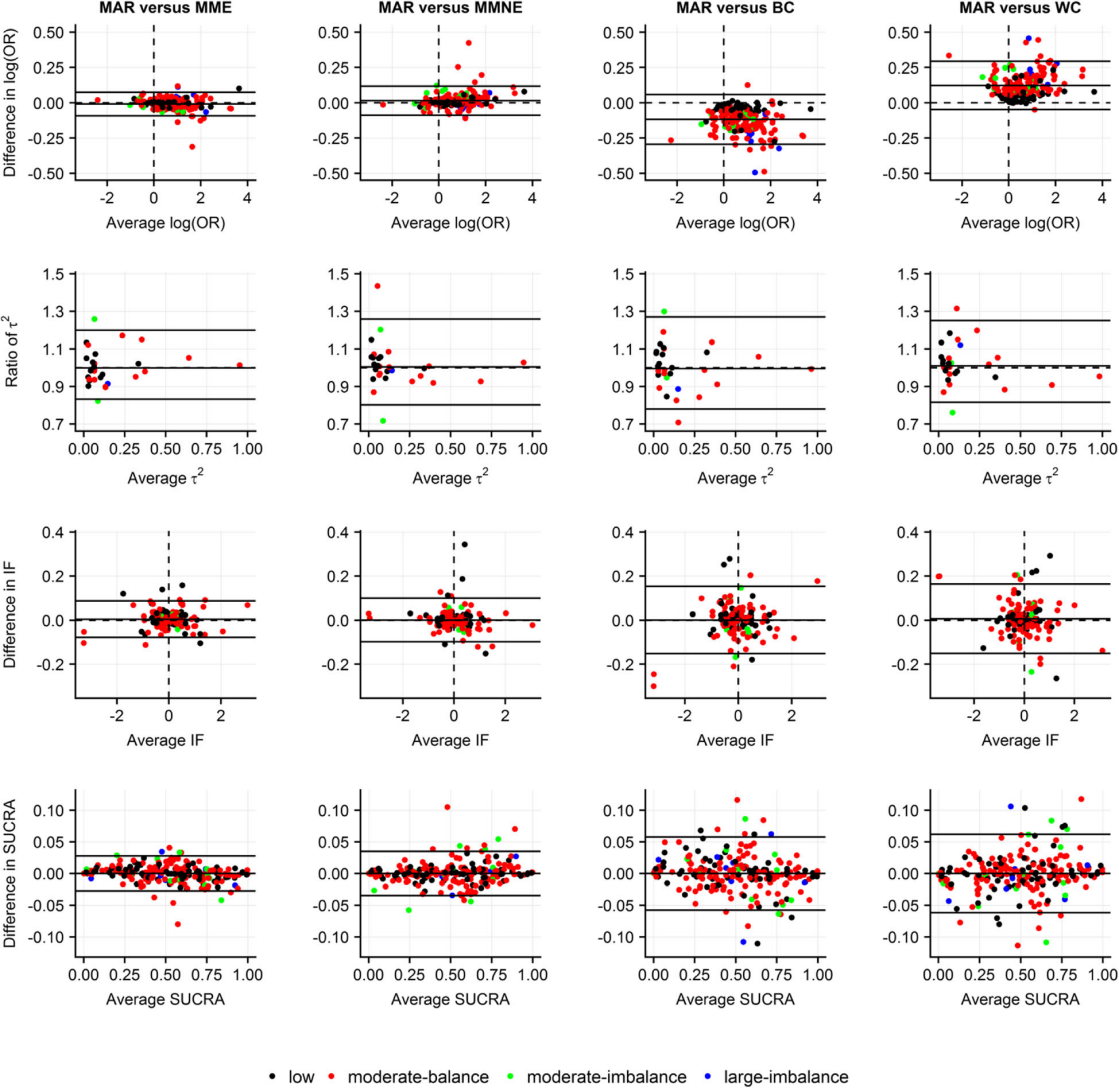
Bland-Altman plots on *level of agreement between on average missing at random and four extreme scenarios* in terms of posterior mean of log odds ratio for basic parameters (first row), posterior median of common between-trial variance (second row), posterior mean of inconsistency factor (third row) and posterior mean of SUCRA values (fourth row). Use of identical, intervention-specific, normal prior distribution on log IMORs with moderate prior variance. Different colors indicate extent and balance of MOD across 29 networks (17 networks with at least one closed loop). BC, best-case scenario; IF, inconsistency factor; MAR, (on average) missing at random; MME, more missing cases are events in all interventions; MMNE, more missing cases are non-events in all interventions; OR, odds ratio; SUCRA, surface under cumulative ranking; WC, worst-case scenario

Overall, differences in terms of posterior mean of log ORs ranged in much narrower LoA for on average MAR versus MME and MMNE as opposed to on average MAR versus BC and WC where almost all differences were concentrated systematically below and above 0, respectively, for networks with moderate and large MOD (Fig. 2). Most ratios were uniformly scattered at low averages of posterior median of *τ*^2^ s (approximately below 0.15). In line with log ORs, differences in terms of posterior mean of IFs and posterior mean of SUCRAs, as well as ratios in terms of posterior standard deviations, ranged overall in narrower LoA for on average MAR versus MME and MMNE as opposed to on average MAR versus BC and WC (Fig. 2; Additional file 5: Figure S1(a)). Generally, there were small perturbations in posterior median of rankings (Additional file 5: Figure S1(b)).

### Implications of discounting uncertainty due to MOD

**Fig. 3:**
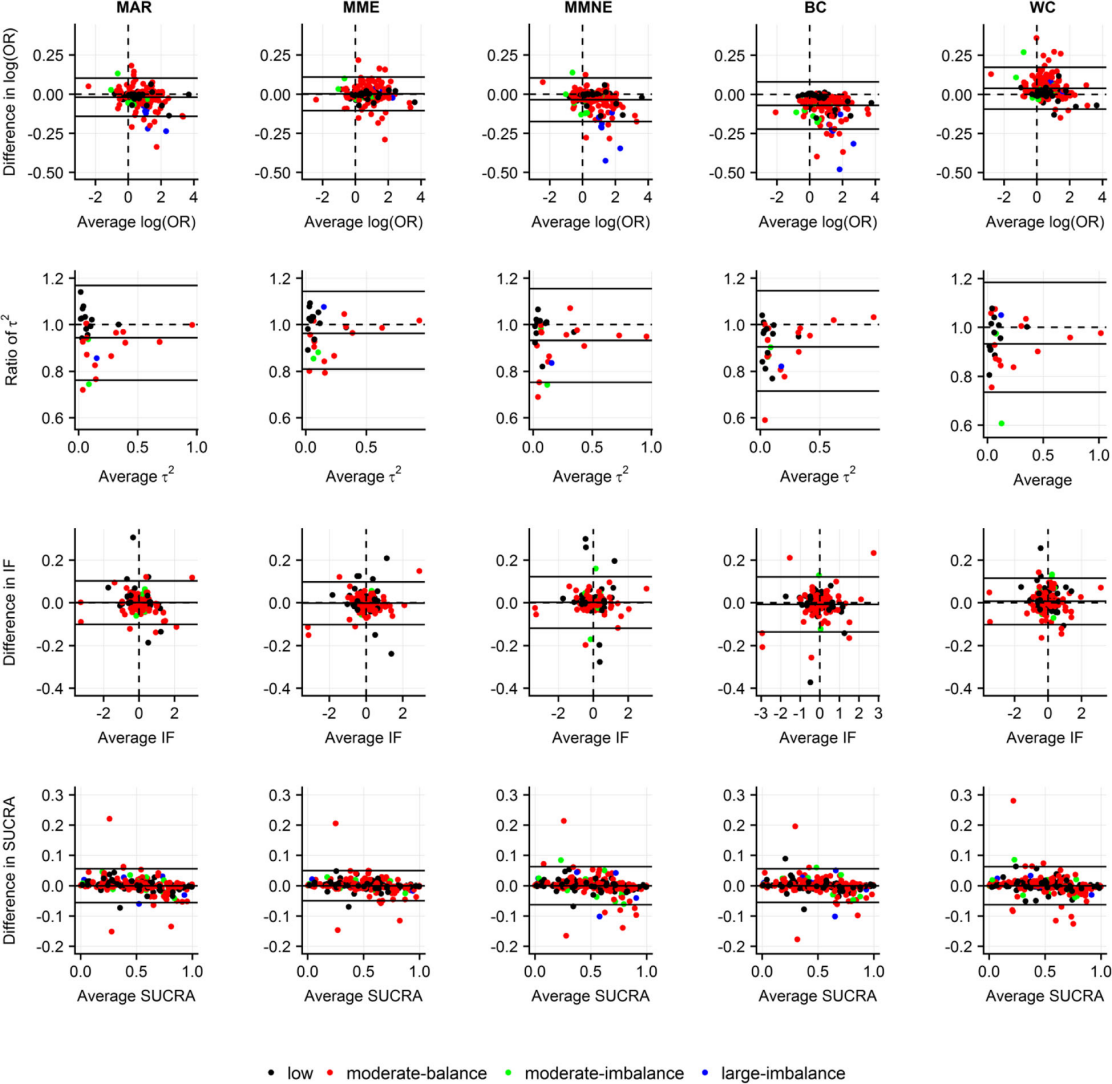
Bland-Altman plots on *level of agreement between accountability and ignorance of uncertainty due to MOD* under missing at random and four extreme scenarios in terms of posterior mean of log odds ratio for basic parameters (first row), posterior median of common between-trial variance (second row), posterior mean of inconsistency factors (third row) and posterior mean of SUCRA values (fourth row). Use of identical, intervention-specific, normal prior distribution on log IMORs with moderate and zero prior variance to reflect accountability and ignorance of uncertainty due to MOD, respectively. Different colors indicate extent and balance of MOD across 29 networks (17 networks with at least one closed loop). BC, best-case scenario; IF, inconsistency factor; MAR, missing at random; MME, more missing cases are events in all interventions; MMNE, more missing cases are non-events in all interventions; OR, odds ratio; SUCRA, surface under cumulative ranking; WC, worst-case scenario

Discounting uncertainty due to MOD led to systematically larger posterior mean of log ORs for MMNE and BC scenarios, yet systematically smaller posterior mean of log ORs for WC scenario, especially for moderate and large MOD (Fig. 3). The majority of ratios of posterior standard deviation of log ORs were systematically above 1 across all scenarios indicating a tendency for increased precision when uncertainty due to MOD was ignored (Additional file 5: Figure S2(a)).

Interestingly, posterior median of *τ*^2^ s was systematically larger when uncertainty due to MOD was ignored regardless of scenario (Fig. 3). Overall, ignoring uncertainty due to MOD led to slightly smaller and larger posterior mean of SUCRAs for averages below 50% and above 75%, respectively, regardless of scenario. Most differences in posterior standard deviation of SUCRAs were systematically positive across all scenarios after discounting uncertainty due to MOD, indicating a tendency for increased precision. Generally, there was little implication for posterior median of rankings (Additional file 5: Figure S2(b)).

### Agreement between identical and hierarchical prior structure for ***δ***_***ik***_

**Fig. 4:**
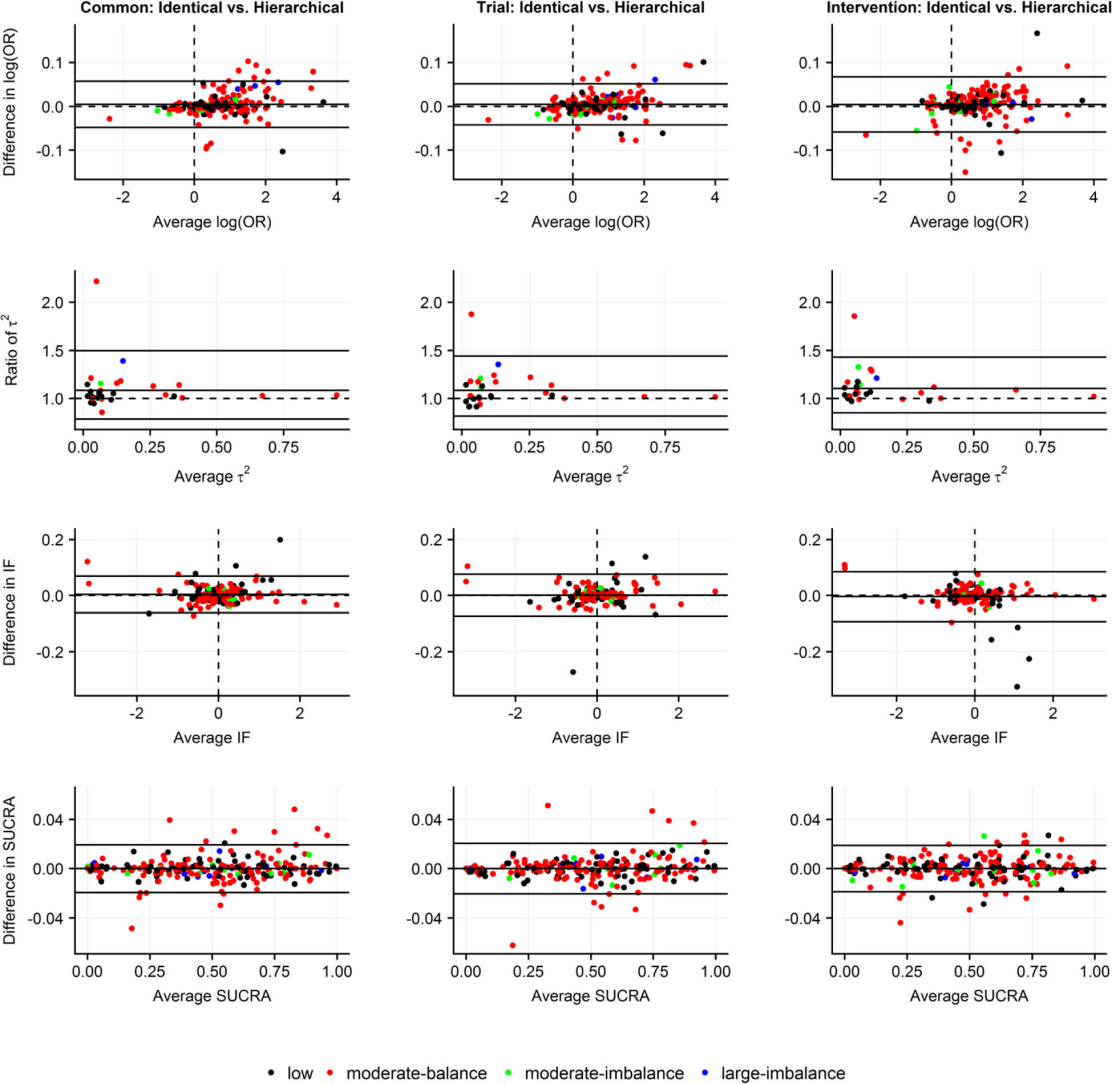
Bland-Altman plots on *level of agreement between identical and hierarchical structure of log IMORs* in terms of posterior mean of log odds ratio for basic parameters (first row), posterior median of common between-trial variance (second row), posterior mean of inconsistency factors (third row) and posterior mean of SUCRA values (fourth row) with respect to common-within-network, trial-specific and intervention-specific normal prior distribution on log IMORs under on average missing at random with moderate prior variance. Different colors indicate extent and balance of MOD across 29 networks (17 networks with at least one closed loop). Common, common-within-network; IF, inconsistency factor; Intervention, intervention-specific; OR, odds ratio; SUCRA, surface under cumulative ranking; Trial, trial-specific

Imposing identical, as opposed to hierarchical, structure on *δ*_*ik*_ led to systematically larger posterior median of *τ*^2^ s across all structural assumptions for *δ*_*ik*_; however, ratios of posterior standard deviation of *τ*^2^ s were uniformly scattered (Fig. 4; Additional file 5: Figure S3(a)). Overall, differences ranged in quite narrow LoA in terms of posterior mean of log ORs (mostly in the case of low MOD), posterior mean of IFs and posterior mean of SUCRAs, as well as ratios of posterior standard deviations (especially for log ORs and SUCRAs under intervention-specific assumption) (Fig. 4; Additional file 5: Figure S3(a)). In general, perturbations for posterior median of rankings were small (Additional file 5: Figure S3(b)).

In all structural assumptions, the majority of differences in posterior mean of log IMORs (i.e. *δ* s and *Δ* s for identical and hierarchical structure, respectively), especially those corresponding to networks with low MOD, were uniformly scattered around 0 and in a range from − 0.25 to 0.25 averages of posterior mean of log IMORs (Additional file 5: Figure S3(c)). Ratios of posterior standard deviation of log IMORs were also scattered uniformly in narrow LoA (especially under the trial-specific structure).

### Agreement among different prior structures for ***δ***_***ik***_

Under identical structure, differences in terms of posterior mean of log ORs, posterior mean of IFs and posterior mean of SUCRAs as well as ratios of posterior standard deviations were scattered in narrower LoA when common-within-network was compared with trial-specific prior structure (Additional file 5: Figure S4(a-c)). Particularly interesting were the results on posterior standard deviation of log ORs and SUCRAs as they were systematically larger under intervention-specific prior structure, especially in the case of moderate and large MOD (Additional file 5: Figure S4(b)). Under hierarchical structure, inferences were similar to those under identical structure for all NMA components (Additional file 5: Figure S5(a-c)).

### Agreement between pattern-mixture model and selection model

**Fig. 5:**
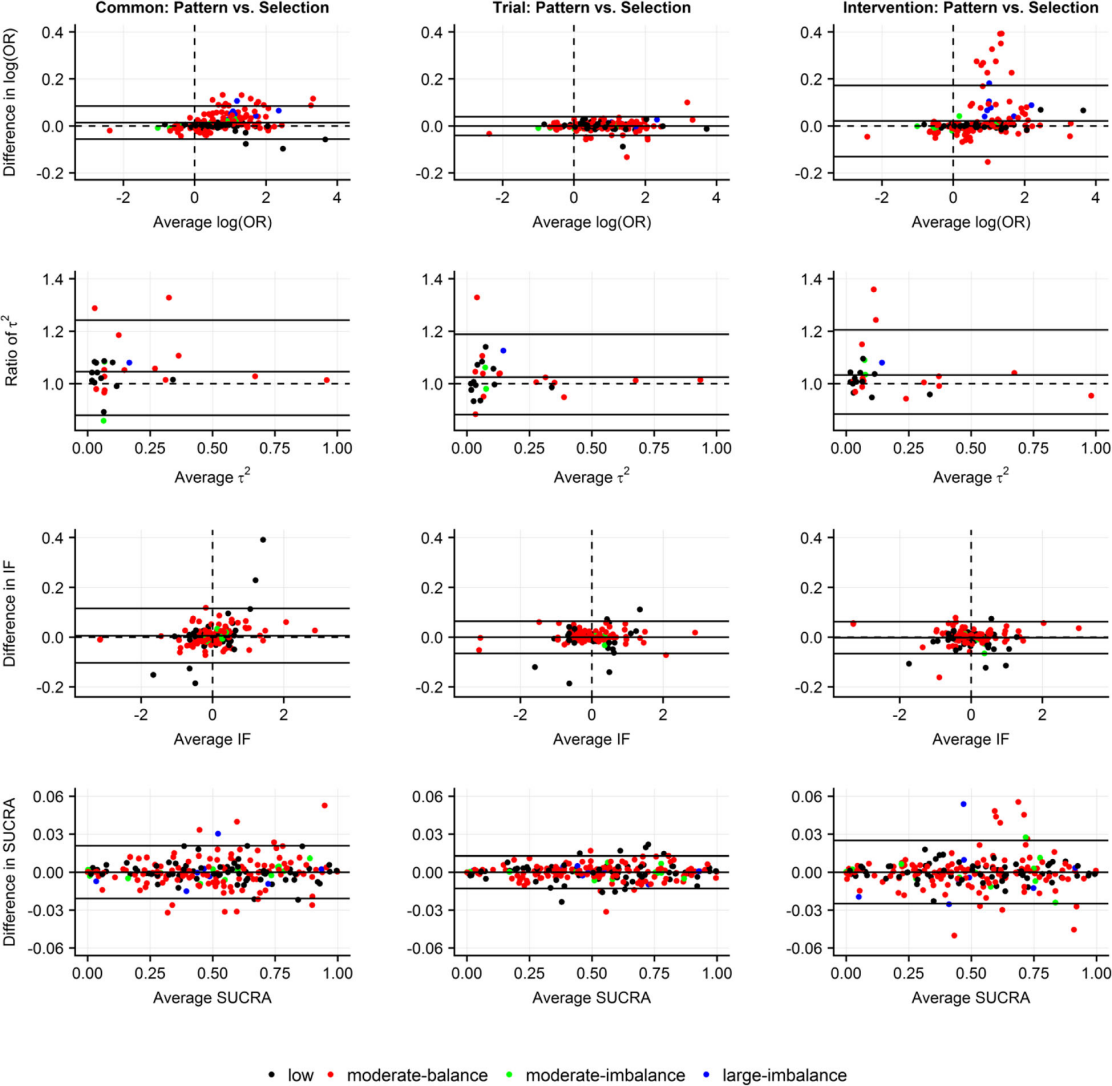
Bland-Altman plots on *level of agreement between pattern-mixture model and selection model* in terms of posterior mean of log odds ratio for basic parameters (first row), posterior median of common between-trial variance (second row), posterior mean of inconsistency factors (third row) and posterior mean of SUCRA values (fourth row) with respect to common-within-network, trial-specific and intervention-specific normal prior distribution on identical log IMORs under on average missing at random with moderate prior variance. Different colors indicate extent and balance of MOD across 29 networks (17 networks with at least one closed loop). Common, common-within-network; IF, inconsistency factor; Intervention, intervention-specific; OR, odds ratio; SUCRA, surface under cumulative ranking; Trial, trial-specific

Assuming common-within-network or intervention-specific prior structure on identical *δ*_*ik*_ led to relatively wider LoA for posterior mean of log ORs and SUCRAs as opposed to trial-specific prior structure where differences were uniformly scattered in narrower LoA (Fig. 5). Overall, ratios of posterior standard deviation of all NMA estimates were scattered in narrow LoA (especially for trial-specific structure) (Additional file 5: Figure S6(a)). Perturbations for posterior median of rankings were small (Additional file 5: Figure S6(b)). Most posterior means of *δ*_*ik*_ s were scattered uniformly around 0 and in a range from − 0.5 to 0.5 averages of posterior mean of *δ*_*ik*_ s for all prior structures (Additional file 5: Figure S6(c)). Results for posterior standard deviation of *δ*_*ik*_ s were particularly interesting: selection model led to systematically imprecise *δ*_*ik*_ s more frequently than pattern-mixture model for all prior structures and especially for moderate and large MOD.

Overall, there was agreement in strength and direction of posterior mean of log ORs and posterior mean of IFs in all analyses (Additional file 1: Tables S4 – S9). The level of agreement in extent of *τ*^2^ could not be judged with confidence due to few estimated *τ*^2^*s* (only 29).

### Additional analysis

#### Different prior values for the variance of ***δ***_***ik***_

Using conservative prior variance led to systematically smaller posterior median of *τ*^2^ s, yet systematically larger posterior standard deviation of log ORs and posterior standard deviation of SUCRAs (Additional file 5: Figure S7(a)). Contrarily, using liberal prior variance led to systematically smaller posterior standard deviations of log ORs and SUCRAs. Overall, differences between moderate and conservative prior variance ranged within wider LoA in terms of posterior distribution of NMA estimates as compared to differences between moderate and liberal prior variance. Implications for posterior median of rankings were small (Additional file 5: Figure S7(b)). There was poor agreement between moderate and alternative prior variances in terms of posterior mean and posterior standard deviation of *δ*_*ik*_ s as indicated by evidence of proportional bias (Additional file 5: Figure S7(c)). Compared to moderate prior variance, posterior mean of *δ*_*ik*_ s was scattered across twice the range under conservative variance but half the range under liberal prior variance (Additional file 5: Figure S7(d)). Furthermore, posterior standard deviation of *δ*_*ik*_ s did not concur between moderate and alternative prior variances as the former always gave smaller and larger posterior standard deviations compared to conservative and liberal prior variance, respectively (Additional file 5: Figure S7(d)). Overall, there was good agreement in strength and direction of posterior mean of log ORs and posterior mean of IFs (Additional file 1: Table S10). The level of agreement in the extent of *τ*^2^ could not be judged with confidence.

## Discussion

Using a collection of 29 NMAs from a wide range of health-related fields [16], we have performed the first empirical study on the most frequently described Bayesian modelling strategies for *binary* MOD in meta-analyses and elucidated their implications for core NMA estimates.

We found that consideration of BC or WC resulted systematically in much larger and lower log ORs, respectively, particularly when the network was predominated by trials with moderate or large MOD (Fig. 2). A number of methodological articles have illustrated these implications in the context of pairwise and network meta-analysis using invented or real-life examples [2, 4, 8, 10, 11]. Some of the authors pronounced these scenarios as being unrealistic for primary and sensitivity analysis, especially for considerable numbers of missing participants in included trials [2].

Furthermore, we revealed that ignorance of uncertainty due to MOD could implicate estimation of NMA components. Specifically, such a strategy yielded systematically smaller posterior standard deviation of log ORs and smaller posterior standard deviation of SUCRA values, systematically larger posterior mean of log ORs and larger posterior median of *τ*^2^ s when coupled with extreme scenarios and slight exaggeration of potency of highly ranked interventions in terms of SUCRA value. In our previous study, we showed that fixing *δ*_*ik*_ s, while considering BC or WC scenarios, considerably perturbed effects of log ORs and inflated *τ*^2^ even in the case of low MOD [11]. White et al. [3], Turner et al. [10], Spineli et al. [11], and Spineli [37] also indicated an association between *τ*^2^ inflation and fixation of the observations or missingness parameter, especially under extreme scenarios. A possible explanation might be that by fixing the observations or missingness parameter, uncertainty about the trial-specific estimates is reduced and hence, the extent of *τ*^2^ is uncovered.

We found that pattern-mixture and selection models yielded similar results, particularly when trial-specific structure was considered for *δ*_*ik*_ s. White et al. [4] compared selection model with pattern-mixture model in a real meta-analysis and found a tendency of the former to provide slightly larger ORs. Nevertheless, we found that selection model yielded imprecise *δ*_*ik*_ s and by extension, reduced our ability to learn about the missingness mechanism with certainty.

Making different assumptions about prior structure of *δ*_*ik*_ added further insights into implications of MOD on NMA estimates. Selecting between identical and hierarchical structure mostly affected estimation of *τ*^2^, whereas the decision to select common-within-network, trial-specific or intervention-specific prior structure for *δ*_*ik*_ mostly implicated uncertainty around the estimation of log ORs and SUCRA values, especially in the case of moderate and large MOD. We found that the intervention-specific structure led to systematically larger posterior standard deviation of log ORs and SUCRAs as opposed to the other prior structures for *δ*_*ik*_. A possible explanation might be the following: since most networks had either low or moderate but balance MOD across trials, the common-within-network and trial-specific structure (which assumed that MOD were equally informative in the whole network or in all arms of each trial, respectively [3]) assigned relatively larger weight on these trials as opposed to the intervention-specific structure (which assumed that MOD were differently informative in the arms of each trial [3]) – the latter was affected by extent of total MOD in each trial [3].

Nevertheless, as mentioned by Turner et al. [10], structural assumptions for the missingness parameter would be best led by experts and tailored to the condition and interventions investigated, since different prior structures may affect our ability to learn about the missingness mechanisms in a specific meta-analysis and by extension, may impact meta-analysis results. In the context of NMA, the analyst deals with multiple interventions that are appointed to a wider patient setting and thus, interventions may bear on different degree of MOD in different comparisons and possibly different missingness mechanisms. Consequently, we view common-within-network to be a rather implausible structure, especially in networks that include interventions of different functionality (e.g. placebo and active interventions), as the missingness mechanisms are expected to differ in different interventions.

The shortcomings of our study must be acknowledged. First, we were able to extract arm-level binary outcome data in every trial in only 29 (11%) out of 273 NMAs with MOD due to severe limitations in reporting quality of the reviews [16]. As a result, there was scarcity of points in Bland-Altman plots for *τ*^2^ and *δ*_*ik*_ s for the common-within-network structure that prevented us from fully understanding method performance when compared for these components. Nevertheless, we would not expect our conclusions to differ should a larger dataset be collected. Furthermore, the limited extracted networks did not allowed us to thoroughly learn about the implications of extent of MOD (in terms of prevalence and imbalance) on NMA estimates since relevant groups (as defined in Methods under Characterising networks based on prevalence and balance of MOD) were considerably unbalanced in frequency (Results under Definition of MOD across health-related fields).

Second, using the extraction criteria we developed in a previous work [38], we found that extraction quality was *unacceptable* in 23 (79%) reviews, because reviewers provided no information on observed outcome or how MOD were handled, whereas for the remaining 6 reviews, extraction was judged as *unclear*, since only information on observed outcome was unavailable (Additional file 1: Table S11). Consequently, no distinction could be made between observed and imputed outcomes in order to achieve an accurate extraction. In nine networks, unacceptable extraction manifested as calculated negative non-events in some of the included trials, which we removed in order to be able to perform NMA. For discussion on the issue of negative non-events the reader could refer to Spineli [38].

Ideally, good agreement should reflect clinically meaningful differences in measurements of compared methods [28]. We determined two methods as having good agreement when average bias was close to 0 (for differences) or 1 (for ratios) and points were uniformly scattered within narrow LoA. Since we dealt with many different conditions and clinical outcomes, it was not possible to decide in advance on a specific clinically meaningful average bias that would indicate good agreement between compared methods.

Finally, normal prior distributions on log IMORs were specified using values for mean and variance as recommended in relevant methodological articles [3, 4, 11] rather than based on expert opinion. Ideally, informative prior distributions should be elicited tailored to the clinical condition and interventions studied, since the extent and reasons for MOD are expected to vary across different conditions and interventions [10]. Empirical elicitation studies are needed to provide us with proper prior distributions for log IMORs.

### Recommendations for good practice

While the focus of our study was on systematic reviews with NMA, the following recommendations also apply to systematic reviews with pairwise meta-analyses.

- In line with other authors [2, 39–41], perform a primary analysis under on average MAR assumption, and opt for assumptions with clinical plausibility as sensitivity analyses in order to explore robustness of primary analysis results.
- Avoid fixing the dataset either by imputing or excluding MOD before analysis and instead, opt for modelling the missingness mechanism via the IMOR parameter in order to accommodate uncertainty about the missingness scenarios considered.
- Consider hierarchical rather than identical structure on *δ*_*ik*_ s when MOD are substantial. Nevertheless, further research is needed to clarify conditions for proper utilization of each structure.
- Opt for trial-specific prior structure on *δ*_*ik*_ s when compared interventions are believed to trigger similar missingness mechanisms as opposed to trial set-up. Consider intervention-specific prior structure on *δ*_*ik*_ s when missingness mechanisms are believed to differ across interventions. Avoid the common-within-network prior structure, especially in the case of moderate or large MOD. Consult an expert to discuss the prior structure on *δ*_*ik*_ that best fits collected trials (i.e. good knowledge of the specific examples being considered and detailed inspection of the properties of included trials is desired). In line with the aforementioned point, further research is needed to comprehend performance of NMA components under different prior structures for *δ*_*ik*_ s in depth.
- When low MOD is present, choice between pattern-mixture and selection models could be based upon conceptual and computational convenience for the researcher. For considerable MOD, pattern-mixture model tends to preserve precision in estimation of *δ*_*ik*_ s. Nevertheless, further research is needed to understand when it is most proper to use one model over the other.
- In terms of prior variance for *δ*_*ik*_, select liberal prior variance (*σ*^2^ = 0.25) for large MOD and moderate prior variance (*σ*^2^ = 1) for moderate MOD in order to preserve precision in NMA estimates.

## Conclusions

Addressing MOD using extreme scenarios and/or ignoring uncertainty induced by MOD constitutes naïve strategy with serious implication for NMA estimates, especially when participant losses in included trials are substantial. Instead, aiming to model MOD via the log IMOR parameter can ensure credible NMA results via adjustment of attrition bias and, furthermore, offer valuable insights into underlying missingness mechanisms. Researchers should consult an expert in order to decide on the structure of log IMOR that best aligns to the condition and intervention studied and, in addition, to define parameter values of prior distribution for log IMOR.

## Additional files

Additional file 1: **Table S1.** Overview of published methodological and tutorial articles on missing *binary* outcome data in systematic reviews. **Table S2**. Distribution of total percentage of missing outcome data per network. **Table S3**. Distribution of the difference in %MOD between compared arms per network. **Table S4**. Agreement on direction, strength of evidence and extent of heterogeneity. **Table S5**. Agreement on direction, strength of evidence and extent of heterogeneity. **Table S6**. Agreement on direction, strength of evidence and extent of heterogeneity. **Table S7**. Agreement on direction, strength of evidence and extent of heterogeneity. **Table S8**. Agreement on direction, strength of evidence and extent of heterogeneity. **Table S9**. Agreement on direction, strength of evidence and extent of heterogeneity. **Table S10**. Agreement on direction, strength of evidence and extent of heterogeneity. **Table S11**. Judgment of accuracy extraction of the eligible networks with justifications. (DOCX 102 kb)

Additional file 2: Supplementary information of the Methods. (DOCX 184 kb)

Additional file 3: Code for all network meta-analysis models. (DOCX 41 kb)

Additional file 4 Analysed dataset of 29 network meta-analyses and selected empirical prior distributions for between-trial variance. (TXT 39 kb)

Additional file 5: Supplementary Figures. (DOCX 5671 kb)

## Abbreviations

BC: Best-case scenario for all non-reference interventions
IF: Inconsistency factor
IMOR: Informative missingness odds ratio
LoA: Limits of agreement
MAR: Missing at random
MME: More missing cases are event in all interventions
MMNE: More missing cases are non-event in all interventions
MOD: Missing outcome data
NMA: Network meta-analysis
OR: Odds ratio
SUCRA: Surface under the cumulative ranking curve
WC: Worst-case scenario for all non-reference interventions

## References

[1] Gamble C, Hollis S (2005). Uncertainty method improved on best-worst case analysis in a binary meta-analysis. J Clin Epidemiol.

[2] Higgins JP, White IR, Wood AM (2008). Imputation methods for missing outcome data in meta-analysis of clinical trials. Clin Trials..

[3] White IR, Higgins JP, Wood AM (2008). Allowing for uncertainty due to missing data in meta-analysis--part 1: two-stage methods. Stat Med.

[4] White IR, Welton NJ, Wood AM, Ades AE, Higgins JP (2008). Allowing for uncertainty due to missing data in meta-analysis--part 2: hierarchical models. Stat Med.

[5] Yuan Y, Little RJ (2009). Meta-analysis of studies with missing data. Biometrics..

[6] Akl EA, Johnston BC, Alonso-Coello P, Neumann I, Ebrahim S, Briel M (2013). Addressing dichotomous data for participants excluded from trial analysis: a guide for systematic reviewers. PLoS One.

[7] Ebrahim S, Akl EA, Mustafa RA, Sun X, Walter SD, Heels-Ansdell D (2013). Addressing continuous data for participants excluded from trial analysis: a guide for systematic reviewers. J Clin Epidemiol.

[8] Mavridis D, White IR, Higgins JP, Cipriani A, Salanti G (2015). Allowing for uncertainty due to missing continuous outcome data in pairwise and network meta-analysis. Stat Med.

[9] Dimitrakopoulou V, Efthimiou O, Leucht S, Salanti G (2015). Accounting for uncertainty due to 'last observation carried forward' outcome imputation in a meta-analysis model. Stat Med.

[10] Turner NL, Dias S, Ades AE, Welton NJ (2015). A Bayesian framework to account for uncertainty due to missing binary outcome data in pairwise meta-analysis. Stat Med.

[11] Spineli LM, Higgins JP, Cipriani A, Leucht S, Salanti G (2013). Evaluating the impact of imputations for missing participant outcome data in a network meta-analysis. Clin Trials..

[12] White IR, Higgins JP (2009). Meta-analysis with missing data. Stata J.

[13] Higgins JPT, Deeks JJ, Altman DG. Special topics in statistics. In: JPT H, Green S, editors. Cochrane handbook for systematic reviews of interventions. Version 5.1.0 (updated March 2011); 2011. The Cochrane Collaboration. http://handbook-5-1.cochrane.org/.

[14] Efthimiou O, Debray TP, van Valkenhoef G, Trelle S, Panayidou K, Moons KG (2016). GetReal in network meta-analysis: a review of the methodology. Res Synth Methods.

[15] Petropoulou M, Nikolakopoulou A, Veroniki AA, Rios P, Vafaei A, Zarin W (2017). Bibliographic study showed improving statistical methodology of network meta-analyses published between 1999 and 2015. J Clin Epidemiol.

[16] Spineli LM, Yepes-Nuñez JJ, Schünemann HJ (2018). A systematic survey shows that reporting and handling of missing outcome data in networks of interventions is poor. BMC Med Res Methodol.

[17] Lee AW (2014). Review of mixed treatment comparisons in published systematic reviews shows marked increase since 2009. J Clin Epidemiol.

[18] Nikolakopoulou A, Chaimani A, Veroniki AA, Vasiliadis HS, Schmid CH, Salanti G (2014). Characteristics of networks of interventions: a description of a database of 186 published networks. PLoS One.

[19] Chambers JD, Naci H, Wouters OJ, Pyo J, Gunjal S, Kennedy IR (2015). An assessment of the methodological quality of published network meta-analyses: a systematic review. PLoS One.

[20] Walter SD (2000). Choice of effect measure for epidemiological data. J Clin Epidemiol.

[21] Sackett DL, Richardson WS, Rosenberg W, Haynes RB (1997). Evidence-based medicine: how to practice and teach EBM.

[22] Dias S, Sutton AJ, Ades AE, Welton NJ (2013). Evidence synthesis for decision making 2: a generalized linear modeling framework for pairwise and network meta-analysis of randomized controlled trials. Med Decis Mak.

[23] Magder LS (2003). Simple approaches to assess the possible impact of missing outcome information on estimates of risk ratios, odds ratios, and risk differences. Control Clin Trials.

[24] Lambert PC, Sutton AJ, Burton PR, Abrams KR, Jones DR (2005). How vague is vague? A simulation study of the impact of the use of vague prior distributions in MCMC using WinBUGS. Stat Med.

[25] van Valkenhoef G, Dias S, Ades AE, Welton NJ (2016). Automated generation of node-splitting models for assessment of inconsistency in network meta-analysis. Res Synth Methods.

[26] Dias S, Welton NJ, Caldwell DM, Ades AE (2010). Checking consistency in mixed treatment comparison meta-analysis. Stat Med.

[27] Salanti G, Ades AE, Ioannidis JP (2011). Graphical methods and numerical summaries for presenting results from multiple-treatment meta-analysis: an overview and tutorial. J Clin Epidemiol.

[28] Bland JM, Altman DG (1999). Measuring agreement in method comparison studies. Stat Methods Med Res.

[29] Cohen J (1960). A coefficient of agreement for nominal scales. Educ Psychol Meas.

[30] Landis JR, Koch GG (1977). The measurement of observer agreement for categorical data. Biometrics..

[31] Turner RM, Jackson D, Wei Y, Thompson SG, Higgins JP (2015). Predictive distributions for between-study heterogeneity and simple methods for their application in Bayesian meta-analysis. Stat Med.

[32] Su YS, Yajima M. R2jags: Using R to run ‘JAGS’. R package version 0.5–7. 2015. https://CRAN.R-project.org/package=R2jags.

[33] R Core Team. A Language and Environment for Statistical Computing. Vienna; 2016. https://www.r-project.org.

[34] van Valkenhoef G, Kuiper J. gemtc: Network meta-analysis using Bayesian methods. R package version 0.8-2, 2016. https://github.com/gertvv/gemtc.

[35] Chang W (2013). R Graphics Cookbook: practical recipes for visualizing data. 1st ed.

[36] Wilke C. cowplot: Streamlined plot theme and plot annotations for ‘ggplot2’. R package version 0.9–3. 2017. https://github.com/wilkelab/cowplot.

[37] Spineli LM (2019). Modeling missing binary outcome data while preserving transitivity assumption yielded more credible network meta-analysis results. J Clin Epidemiol.

[38] Spineli LM (2017). Missing binary data extraction challenges from Cochrane reviews in mental health and Campbell reviews with implications for empirical research. Res Synth Methods.

[39] White IR, Carpenter J, Horton NJ (2012). Including all individuals is not enough: lessons for intention-to-treat analysis. Clin Trials.

[40] Guyatt GH, Ebrahim S, Alonso-Coello P, Johnston BC, Mathioudakis AG, Briel M (2017). GRADE guidelines 17: assessing the risk of bias associated with missing participant outcome data in a body of evidence. J Clin Epidemiol.

[41] Akl EA, Kahale LA, Agoritsas T, Brignardello-Petersen R, Busse JW, Carrasco-Labra A (2015). Handling trial participants with missing outcome data when conducting a meta-analysis: a systematic survey of proposed approaches. Syst Rev.

