# Supplementary material for: An empirical comparison of Bayesian modelling strategies for missing binary outcome data in network meta-analysis
Source: BMC Med Res Methodol. 2019 Apr 24;19:86. doi: 10.1186/s12874-019-0731-y (PMC6480793; doi:10.1186/s12874-019-0731-y)
Supplement: Supplementary file 1 — Table S1. Overview of published methodological and tutorial articles on missing binary outcome data in systematic reviews. Table S2. Distribution of total percentage of missing outcome data per network. Table S3. Distribution of the difference in %MOD between compared arms per network. Table S4. Agreement on direction, strength of evidence and extent of heterogeneity. Table S5. Agreement on direction, strength of evidence and extent of heterogeneity. Table S6. Agreement on direction, strength of evidence and extent of heterogeneity. Table S7. Agreement on direction, strength of evidence and extent of heterogeneity. Table S8. Agreement on direction, strength of evidence and extent of heterogeneity. Table S9. Agreement on direction, strength of evidence and extent of heterogeneity. Table S10. Agreement on direction, strength of evidence and extent of heterogeneity. Table S11. Judgment of accuracy extraction of the eligible networks with justifications. (DOCX 102 kb) [file 12874_2019_731_MOESM1_ESM.docx]

**Additional file 1. Supplementary Tables**

**Table S1.** Overview of published methodological and tutorial articles on missing *binary* outcome data in systematic reviews

| **Article** | **MOD methods** | **MOD assumption** | **Missingness parameter** | **Missingness parameter structure** | **Analysis framework** | **MA model** | **MA parameters** | **Other** |
| --- | --- | --- | --- | --- | --- | --- | --- | --- |
| *Pairwise meta-analysis* | | | | | | | | |
| [1] | Direct imputation | MAR, BC, WC, UI | – | – | Frequentist | FE | MA OR | With and without accountability of MOD uncertainty |
| [2] | Direct imputation, two-stage pattern-mixture model | MAR, UI, AME, AMNE, BC, WC, pC, pE, p | IMOR* | identical intervention-specific | Frequentist | FE and RE^1^ | MA RR, $I^{2}$ | Various weighting schemes for MOD uncertainty |
| [3] | Two-stage pattern-mixture model | MAR, AME, AMNE, BC, WC | IMOR* | identical trial-specific, unconditional, correlated | TS, GH | FE and RE | MA OR, $\tau^{2}$ | – |
| [4] | Two-stage pattern-mixture model, One-stage selection model | MAR, UI, BC, WC | IMOR* | identical common, identical intervention-specific, identical trial-specific, independent unconditional | Bayesian | FE and RE | MA OR | Different variances for the log IMOR |
| [5] | Direct imputation | MAR, AMNE, AME, BC, WC, pC, p | RI* | identical intervention-specific | Frequentist | FE | MA OR, MA RR | Different values for RI, no accountability of MOD uncertainty |
| [6]^2^ | Direct imputation, two-stage pattern-mixture model | MAR, UI, BC, WC, LOCF | IMOR* | identical intervention-specific | Frequentist | RE | MA OR | With and without accountability of MOD uncertainty |
| [7] | Two-stage pattern-mixture model | MAR, LOCF | Sensitivity and specificity | identical intervention-specific | Bayesian | RE | MA OR, $\tau^{2}$ | Different priors on diagnostic parameters |
| [8] | One-stage pattern-mixture model, direct imputation | MAR, AMNE^3^, BC^3^, WC^3^ | $p^{m}$, IMOR* | independent common | Bayesian | FE and RE | MA OR, $\tau^{2}$ | Different priors on $p^{m}$, model fit ($\bar{D}_{res}$) |
| *Network meta-analysis* | | | | | | | | |
| [9] | One-stage selection model | MAR, AME, AMNE, BC, WC | IMOR* | identical intervention-specific | Bayesian | RE | NMA OR, $\tau^{2}$, SUCRA | With and without accountability of MOD uncertainty |

AME, more missing cases are events in both arms; AMNE, all missing cases are non-events in both arms; BC, best-case scenario for the experimental arm; $\bar{D}_{res}$, posterior mean of residual deviance; FE, fixed-effect model; GH, Gauss–Hermite approximation; IMOR, informative missingness odds ratio; LOCF, last-observation carried forward; MA, meta-analysis; MAR, missing at random; MOD, missing outcome data; p, arm-specific observed risk; pC, control-specific observed risk; pE, experimental-specific observed risk; $p^{m}$, probability of event among missing participants; RE, random-effects model; RI, relative incidence among those with missing data compared to those with available data in the same arm; SUCRA, surface under the cumulative ranking curve; TS, Taylor series approximation; UI, uncertainty interval by Gamble and Hollis; WC, worst-case scenario for the experimental arm.

^1^Results are not shown in the publication.

^2^A review and tutorial on proposed methods to handle missing binary outcome data in pairwise meta-analysis.

^3^For each assumption, missing outcome data were imputed before meta-analysis and results were compared with those after considering specific missingness parameter(s).

*The respective scenarios imply that it is more or less like that missing cases will have the event in all interventions or the experimental or the control alone.

**References**

1. Gamble C, Hollis S. Uncertainty method improved on best-worst case analysis in a binary meta-analysis. J Clin Epidemiol. 2005;58:579-88.
2. Higgins JP, White IR, Wood AM. Imputation methods for missing outcome data in meta-analysis of clinical trials. Clin Trials. 2008;5:225-39.
3. White IR, Higgins JP, Wood AM. Allowing for uncertainty due to missing data in meta-analysis--part 1: two-stage methods. Stat Med. 2008;27:711-27.
4. White IR, Welton NJ, Wood AM, Ades AE, Higgins JP. Allowing for uncertainty due to missing data in meta-analysis--part 2: hierarchical models. Stat Med. 2008;27:728-45.
5. Akl EA, Johnston BC, Alonso-Coello P, Neumann I, Ebrahim S, Briel M, et al. Addressing dichotomous data for participants excluded from trial analysis: a guide for systematic reviewers. PLoS One. 2013;8:e57132.
6. Mavridis D, Chaimani A, Efthimiou O, Leucht S, Salanti G. Addressing missing outcome data in meta-analysis. Evid Based Ment Health. 2014;17:85-9.
7. Dimitrakopoulou V, Efthimiou O, Leucht S, Salanti G. Accounting for uncertainty due to 'last observation carried forward' outcome imputation in a meta-analysis model. Stat Med. 2015;34:742-52.
8. Turner NL, Dias S, Ades AE, Welton NJ. A Bayesian framework to account for uncertainty due to missing binary outcome data in pairwise meta-analysis. Stat Med. 2015;34:2062-80.
9. Spineli LM, Higgins JP, Cipriani A, Leucht S, Salanti G. Evaluating the impact of imputations for missing participant outcome data in a network meta-analysis. Clin Trials. 2013;10:378-88.

| **Prevalence and balance of missing outcome data in each network** |
| --- |

**Table S2. Distribution of total percentage of missing outcome data per network**

| **ID** | **minimum** | **1^st^ quartile** | **median** | **mean** | **3^rd^ quartile** | **maximum** | **prevalence^†^** |
| --- | --- | --- | --- | --- | --- | --- | --- |
| 1 | 0.00% | 1.47% | 3.63% | 5.46% | 6.94% | 24.14% | Low |
| 2 | 0.49% | 1.34% | 2.15% | 3.33% | 3.06% | 19.00% | Low |
| 3 | 1.44% | 10.17% | 12.91% | 15.24% | 19.10% | 34.72% | Moderate |
| 4 | 1.97% | 2.54% | 4.07% | 4.27% | 6.01% | 6.81% | Low |
| 5 | 1.35% | 3.42% | 4.83% | 4.65% | 5.31% | 8.02% | Low |
| 6 | 1.13% | 1.38% | 2.74% | 4.20% | 6.24% | 10.74% | Low |
| 7 | 0.00% | 7.68% | 13.79% | 13.52% | 17.80% | 42.80% | Moderate |
| 8 | 0.69% | 1.38% | 1.68% | 1.99% | 2.91% | 3.30% | Low |
| 9 | 4.73% | 6.25% | 7.71% | 8.06% | 8.95% | 14.11% | Moderate |
| 10 | 8.99% | 26.75% | 29.07% | 30.98% | 39.78% | 49.66% | Large |
| 11 | 0.00% | 4.46% | 7.46% | 7.71% | 11.02% | 20.00% | Moderate |
| 12 | 0.00% | 13.62% | 18.44% | 17.31% | 22.55% | 29.11% | Moderate |
| 13 | 4.18% | 9.65% | 11.87% | 12.43% | 16.00% | 19.90% | Moderate |
| 14 | 0.00% | 12.07% | 15.65% | 18.45% | 23.81% | 42.55% | Moderate |
| 15 | 0.00% | 0.32% | 8.69% | 9.01% | 17.39% | 18.68% | Moderate |
| 16 | 3.65% | 4.35% | 5.28% | 5.28% | 6.22% | 6.92% | Moderate |
| 17 | 2.10% | 12.57% | 19.28% | 17.85% | 22.64% | 38.23% | Moderate |
| 18 | 0.00% | 12.79% | 17.68% | 17.73% | 23.20% | 38.71% | Moderate |
| 19 | 4.49% | 12.45% | 16.81% | 16.54% | 22.12% | 28.47% | Moderate |
| 20 | 1.89% | 4.41% | 5.22% | 6.07% | 6.07% | 17.46% | Moderate |
| 21 | 1.73% | 1.85% | 4.70% | 4.38% | 6.76% | 6.87% | Low |
| 22 | 3.31% | 8.40% | 14.45% | 18.80% | 24.84% | 57.83% | Moderate |
| 23 | 0.15% | 0.38% | 0.57% | 0.61% | 0.88% | 1.06% | Low |
| 24 | 0.00% | 0.00% | 4.54% | 11.54% | 13.23% | 53.16% | Low |
| 25 | 0.00% | 3.19% | 9.27% | 10.63% | 14.38% | 42.43% | Moderate |
| 26 | 0.00% | 0.00% | 1.43% | 2.13% | 1.64% | 7.56% | Low |
| 27 | 0.00% | 6.89% | 11.08% | 12.54% | 18.12% | 29.77% | Moderate |
| 28 | 0.61% | 1.93% | 3.89% | 5.57% | 7.96% | 19.47% | Low |
| 29 | 0.00% | 0.00% | 0.00% | 2.15% | 0.18% | 14.71% | Low |

**^†^**Missingness was considered to be low for median up to 5% (low attrition bias risk), large for median above 20% (large attrition bias risk) and moderate otherwise (moderate attrition bias).

**Table S3. Distribution of the difference in %MOD between compared arms per network**

| **ID** | **minimum** | **1^st^ quartile** | **median** | **mean** | **3^rd^ quartile** | **maximum** | **balance^†^** |
| --- | --- | --- | --- | --- | --- | --- | --- |
| 1 | 0.00% | 0.63% | 1.89% | 3.07% | 4.29% | 20.88% | Yes |
| 2 | 0.09% | 0.57% | 1.79% | 2.14% | 2.55% | 11.03% | Yes |
| 3 | 0.02% | 4.02% | 5.43% | 6.67% | 8.76% | 15.54% | Yes |
| 4 | 0.65% | 1.21% | 1.95% | 1.69% | 2.21% | 2.32% | Yes |
| 5 | 0.43% | 2.07% | 3.68% | 4.44% | 7.35% | 9.89% | Yes |
| 6 | 0.82% | 1.48% | 3.73% | 4.32% | 7.61% | 8.06% | Yes |
| 7 | 0.00% | 3.67% | 5.62% | 6.96% | 8.79% | 19.28% | Yes |
| 8 | 0.41% | 0.87% | 1.47% | 1.89% | 2.93% | 3.90% | Yes |
| 9 | 0.56% | 1.65% | 3.44% | 4.15% | 4.87% | 10.98% | Yes |
| 10 | 6.81% | 17.11% | 20.58% | 24.93% | 25.85% | 57.35% | No |
| 11 | 0.00% | 0.20% | 2.40% | 4.50% | 4.79% | 23.81% | Yes |
| 12 | 0.00% | 2.47% | 6.38% | 8.08% | 13.84% | 20.15% | Yes |
| 13 | 1.45% | 6.27% | 10.37% | 10.14% | 12.51% | 19.61% | No |
| 14 | 0.00% | 1.53% | 4.44% | 6.94% | 11.41% | 17.56% | Yes |
| 15 | 0.00% | 0.47% | 2.21% | 2.06% | 3.80% | 3.83% | Yes |
| 16 | 0.02% | 0.48% | 0.78% | 0.77% | 1.06% | 1.51% | Yes |
| 17 | 0.00% | 2.57% | 6.66% | 8.05% | 9.93% | 23.82% | No |
| 18 | 0.00% | 3.05% | 5.86% | 7.91% | 7.60% | 46.11% | Yes |
| 19 | 0.12% | 1.96% | 4.84% | 5.24% | 7.32% | 12.72% | Yes |
| 20 | 0.00% | 1.53% | 3.18% | 4.02% | 6.80% | 9.52% | Yes |
| 21 | 0.11% | 1.28% | 2.48% | 2.61% | 4.50% | 4.67% | Yes |
| 22 | 0.82% | 3.59% | 5.00% | 6.32% | 8.24% | 15.92% | Yes |
| 23 | 0.00% | 0.07% | 0.11% | 0.18% | 0.32% | 0.39% | Yes |
| 24 | 0.00% | 0.00% | 2.91% | 3.78% | 7.82% | 9.94% | Yes |
| 25 | 0.00% | 1.68% | 3.99% | 5.84% | 8.45% | 26.18% | Yes |
| 26 | 0.00% | 0.00% | 0.08% | 0.25% | 0.51% | 0.68% | Yes |
| 27 | 0.00% | 0.48% | 2.51% | 4.66% | 7.53% | 23.05% | Yes |
| 28 | 0.00% | 0.85% | 1.10% | 1.61% | 1.81% | 5.79% | Yes |
| 29 | 0.00% | 0.00% | 0.00% | 0.14% | 0.00% | 0.97% | Yes |

**^†^**Networks with median larger than 6.5% were considered to have imbalance in MOD between the compared intervention arms.

MOD: missing outcome data

**References of analysed networks**

1. Cipriani A, Furukawa TA, Salanti G, Geddes JR, Higgins JP, Churchill R, et al. Comparative efficacy and acceptability of 12 new-generation antidepressants: a multiple-treatments meta-analysis. Lancet. 2009;373:746-58.
2. Edwards SJ, Clarke MJ, Wordsworth S, Welton NJ. Carbapenems versus other beta-lactams in the treatment of hospitalised patients with infection: a mixed treatment comparison. Curr Med Res Opin. 2009;25:251-61.
3. Baker WL, Baker EL, Coleman CI. Pharmacologic treatments for chronic obstructive pulmonary disease: a mixed-treatment comparison meta-analysis. Pharmacotherapy. 2009;29:891-905.
4. Burch J, Paulden M, Conti S, Stock C, Corbett M, Welton NJ, et al. Antiviral drugs for the treatment of influenza: a systematic review and economic evaluation. Health Technol Assess. 2009;13:1-265, iii-iv.
5. Uthman OA, Abdulmalik J. Comparative efficacy and acceptability of pharmacotherapeutic agents for anxiety disorders in children and adolescents: a mixed treatment comparison meta-analysis. Curr Med Res Opin. 2010;26:53-9.
6. Bottomley JM, Taylor RS, Ryttov J. The effectiveness of two-compound formulation calcipotriol and betamethasone dipropionate gel in the treatment of moderately severe scalp psoriasis: a systematic review of direct and indirect evidence. Curr Med Res Opin. 2011;27:251-68.
7. Costa J, Fareleira F, Ascenção R, Borges M, Sampaio C, Vaz-Carneiro A. Clinical comparability of the new antiepileptic drugs in refractory partial epilepsy: a systematic review and meta-analysis. Epilepsia. 2011;52:1280-91.
8. Makani H, Bangalore S, Romero J, Wever-Pinzon O, Messerli FH. Effect of renin-angiotensin system blockade on calcium channel blocker-associated peripheral edema. Am J Med. 2011;124:128-35.
9. Virgili G, Novielli N, Menchini F, Murro V, Giacomelli G. Pharmacological treatments for neovascular age-related macular degeneration: can mixed treatment comparison meta-analysis be useful? Curr Drug Targets. 2011;12:212-20.
10. Gallego-Galisteo M, Villa-Rubio A, Alegre-del Rey E, Márquez-Fernández E, Ramos-Báez JJ. Indirect comparison of biological treatments in refractory rheumatoid arthritis. J Clin Pharm Ther. 2012;37:301-7.
11. Filippini G, Del Giovane C, Vacchi L, D'Amico R, Di Pietrantonj C, Beecher D, et al. Immunomodulators and immunosuppressants for multiple sclerosis: a network meta-analysis. Cochrane Database Syst Rev. 2013;CD008933.
12. Gao L, Xia L, Zhao FL, Li SC. Clinical efficacy and safety of the newer antiepileptic drugs as adjunctive treatment in adults with refractory partial-onset epilepsy: a meta-analysis of randomized placebo-controlled trials. Epilepsy Res. 2013;103:31-44.
13. Khan N, Shah D, Tongbram V, Verdian L, Hawkins N. The efficacy and tolerability of perampanel and other recently approved anti-epileptic drugs for the treatment of refractory partial onset seizure: a systematic review and Bayesian network meta-analysis. Curr Med Res Opin. 2013;29:1001-13.
14. Liu J, Dong J, Wang L, Su Y, Yan P, Sun S. Comparative efficacy and acceptability of antidepressants in Parkinson's disease: a network meta-analysis. PLoS One. 2013;8:e76651.
15. Mealing S, Barcena L, Hawkins N, Clark J, Eaton V, Hirji I, et al. The relative efficacy of imatinib, dasatinib and nilotinib for newly diagnosed chronic myeloid leukemia: a systematic review and network meta-analysis. Exp Hematol Oncol. 2013;2:5.
16. Wu MS, Tan SC, Xiong T. Indirect comparison of randomised controlled trials: comparative efficacy of dexlansoprazole vs. esomeprazole in the treatment of gastro-oesophageal reflux disease. Aliment Pharmacol Ther. 2013;38:190-201.
17. Dogliotti A, Paolasso E, Giugliano RP. Current and new oral antithrombotics in non-valvular atrial fibrillation: a network meta-analysis of 79 808 patients. Heart. 2014;100:396-405.
18. Kriston L, von Wolff A, Westphal A, Hölzel LP, Härter M. Efficacy and acceptability of acute treatments for persistent depressive disorder: a network meta-analysis. Depress Anxiety. 2014;31:621-30.
19. Roskell NS, Setyawan J, Zimovetz EA, Hodgkins P. Systematic evidence synthesis of treatments for ADHD in children and adolescents: indirect treatment comparisons of lisdexamfetamine with methylphenidate and atomoxetine. Curr Med Res Opin. 2014;30:1673-85.
20. Patel DA, Snedecor SJ, Tang WY, Sudharshan L, Lim JW, Cuffe R, et al. 48-week efficacy and safety of dolutegravir relative to commonly used third agents in treatment-naive HIV-1-infected patients: a systematic review and network meta-analysis. PLoS One. 2014;9:e105653.
21. Fournier M, Germe M, Theobald K, Scholz GH, Lehmacher W. Indirect comparison of lixisenatide versus neutral protamine Hagedorn insulin as add-on to metformin and sulphonylurea in patients with type 2 diabetes mellitus. Ger Med Sci. 2014;12:Doc14.
22. Palmer SC, Saglimbene V, Mavridis D, Salanti G, Craig JC, Tonelli M, et al. Erythropoiesis-stimulating agents for anaemia in adults with chronic kidney disease: a network meta-analysis. Cochrane Database Syst Rev. 2014;CD010590.
23. Mantha S, Ansell J. Indirect comparison of dabigatran, rivaroxaban, apixaban and edoxaban for the treatment of acute venous thromboembolism. J Thromb Thrombolysis. 2015;39:155-65.
24. Singh S, Garg SK, Pardi DS, Wang Z, Murad MH, Loftus EV Jr. Comparative efficacy of pharmacologic interventions in preventing relapse of Crohn's disease after surgery: a systematic review and network meta-analysis. Gastroenterology. 2015;148:64-76.e2.
25. Linde K, Kriston L, Rücker G, Jamil S, Schumann I, Meissner K, et al. Efficacy and acceptability of pharmacological treatments for depressive disorders in primary care: systematic review and network meta-analysis. Ann Fam Med. 2015;13:69-79.
26. Bow EJ, Vanness DJ, Slavin M, Cordonnier C, Cornely OA, Marks D, et al. Systematic review and mixed treatment comparison meta-analysis of randomized clinical trials of primary oral antifungal prophylaxis in allogeneic hematopoietic cell transplant recipients. BMC Infect Dis. 2015;15:128.
27. Tramacere I, Del Giovane C, Salanti G, D'Amico R, Filippini G. Immunomodulators and immunosuppressants for relapsing-remitting multiple sclerosis: a network meta-analysis. Cochrane Database Syst Rev. 2015;CD011381.
28. Miligkos M, Papamichael K, Vande Casteele N, Mantzaris GJ, Gils A, Levesque BG, et al. Efficacy and Safety Profile of Anti-tumor Necrosis Factor-α Versus Anti-integrin Agents for the Treatment of Crohn's Disease: A Network Meta-analysis of Indirect Comparisons. Clin Ther. 2016;38:1342-1358.e6.
29. Vieira MC, Kumar RN, Jansen JP. Comparative effectiveness of efavirenz, protease inhibitors, and raltegravir-based regimens as first-line treatment for HIV-infected adults: a mixed treatment comparison. HIV Clin Trials. 2011;12:175-89.

| **Agreement between on average missing at random and extreme scenarios** |
| --- |

**Table S4. Agreement on direction, strength of evidence and extent of heterogeneity**

| **Basic parameters (log odds ratio)** | | | | | | | | | | | | | | | | | | | | |
| --- | --- | --- | --- | --- | --- | --- | --- | --- | --- | --- | --- | --- | --- | --- | --- | --- | --- | --- | --- | --- |
|  | MME | | | | | MMNE | | | | | BC | | | | | WC | | | | |
| *Strength of evidence*^1^ | | | | | | | | | | | | | | | | | | | | |
| MAR | Weak | | | Strong | | Weak | | | Strong | | Weak | | | Strong | | Weak | | | Strong | |
| Weak | 60% | | | 1% | | 60% | | | 1% | | 53% | | | 8% | | 60% | | | 1% | |
| Strong | 1% | | | 38% | | 1% | | | 38% | | 1% | | | 39% | | 4% | | | 35% | |
| Kappa | 0.98 (0.95, 1.00)* | | | | | 0.98 (0.95, 1.00)* | | | | | 0.83 (0.75, 0.91)^†^ | | | | | 0.89 (0.83, 0.96)* | | | | |
| *Direction of evidence*^2^ | | | | | | | | | | | | | | | | | | | | |
| MAR | First | | | Second | | First | | | Second | | First | | | Second | | First | | | Second | |
| First | 85% | | | 0% | | 85% | | | 0% | | 85% | | | 0% | | 80% | | | 5% | |
| Second | 1% | | | 14% | | 0% | | | 15% | | 2% | | | 12% | | 0% | | | 15% | |
| Kappa | 0.95 (0.89, 1.00)* | | | | | 0.98 (0.94, 1.00)* | | | | | 0.91 (0.82, 1.00)* | | | | | 0.81 (0.70, 0.92)^†^ | | | | |
| *Extent of between-trial variance*^3^ | | | | | | | | | | | | | | | | | | | | |
| MAR | LO | MO | | | LA | LO | MO | | | LA | LO | MO | | | LA | LO | MO | | | LA |
| Low | 62% | 7% | | | 0% | 69% | 0% | | | 0% | 62% | 7% | | | 0% | 69% | 0% | | | 0% |
| Moderate | 0% | 10% | | | 0% | 0% | 10% | | | 0% | 0% | 10% | | | 0% | 3% | 7% | | | 0% |
| Large | 0% | 7% | | | 14% | 0% | 0% | | | 21% | 0% | 7% | | | 14% | 0% | 0% | | | 21% |
| Kappa | 0.73 (0.49, 0.98)^†^ | | | | | 1.00 | | | | | 0.73 (0.49, 0.98)^†^ | | | | | 0.92 (0.78, 1.00)^†^ | | | | |
| **Inconsistency factor (log odds ratio)** | | | | | | | | | | | | | | | | | | | | |
|  | MME | | | | | MMNE | | | | | BC | | | | | WC | | | | |
| *Strength of evidence*^1^ | | | | | | | | | | | | | | | | | | | | |
| MAR | Weak | | Strong | | | Weak | | Strong | | | Weak | | Strong | | | Weak | | Strong | | |
| Weak | 94% | | 0% | | | 94% | | 0% | | | 94% | | 0% | | | 94% | | 0% | | |
| Strong | 0% | | 6% | | | 0% | | 6% | | | 0% | | 6% | | | 1% | | 5% | | |
| Kappa | 1.00 | | | | | 1.00 | | | | | 1.00 | | | | | 1.00 | | | | |
| *Direction of evidence*^2^ | | | | | | | | | | | | | | | | | | | | |
| MAR | Positive | | Negative | | | Positive | | Negative | | | Positive | | Negative | | | Positive | | Negative | | |
| Positive | 46% | | 0% | | | 46% | | 0% | | | 46% | | 0% | | | 46% | | 0% | | |
| Negative | 1% | | 53% | | | 1% | | 53% | | | 1% | | 53% | | | 1% | | 53% | | |
| Kappa | 0.98 (0.96, 1.00)* | | | | | 0.97 (0.93, 1.00)* | | | | | 0.96 (0.91, 1.00)* | | | | | 0.98 (0.96, 1.00)* | | | | |

BC: best-case scenario; LA: large; LO: low; MAR: (on average) missing at random; MME: more missing cases are events in all interventions; MMNE: more missing cases are non-events in all interventions; MO: moderate; WC: worst-case scenario.

^1^Strong evidence when 0 (in the log scale) is not included in the 95% credible interval, otherwise weak evidence.

^2^Whether the estimated log odds ratio favors the first or second intervention in a comparison. In case of inconsistency factor, whether the difference between direct and indirect estimate for a specific comparison is positive or negative.

^3^Estimated between-trial variance is low, moderate and large when it is smaller than the median, between the median and 3^rd^ quartile and larger than the 3^rd^ quartile, respectively, of the selected empirical distribution for the true between-trial variance.

*Almost perfect agreement (0.81 – 1.00);

^†^95% confidence interval is too wide to judge the level of agreement with confidence.

| **Agreement between accounting and discounting uncertainty due to missingness** |
| --- |

**Table S5. Agreement on direction, strength of evidence and extent of heterogeneity**

| **Basic parameters (log odds ratio)** | | | | | | | | | | | | | | | | | | | | | | | | |
| --- | --- | --- | --- | --- | --- | --- | --- | --- | --- | --- | --- | --- | --- | --- | --- | --- | --- | --- | --- | --- | --- | --- | --- | --- |
|  | MAR w/o | | | | | MME w/o | | | | | MMNE w/o | | | | | BC w/o | | | | | WC w/o | | | |
| *Strength of evidence*^1^ | | | | | | | | | | | | | | | | | | | | | | | | |
| With | Weak | | | Strong | | Weak | | | Strong | | Weak | | | Strong | | Weak | | | Strong | | Weak | | Strong | |
| Weak | 56% | | | 5% | | 57% | | | 4% | | 57% | | | 4% | | 49% | | | 5% | | 61% | | 3% | |
| Strong | 0% | | | 39% | | 0% | | | 39% | | 0% | | | 39% | | 0% | | | 46% | | 0% | | 35% | |
| Kappa | 0.90 (0.84, 0.96)* | | | | | 0.92 (0.86, 0.98)* | | | | | 0.92 (0.86, 0.98)* | | | | | 0.90 (0.84, 0.96)* | | | | | 0.93 (0.87, 0.98)* | | | |
| *Direction of evidence*^2^ | | | | | | | | | | | | | | | | | | | | | | | | |
| With | First | | | Second | | First | | | Second | | First | | | Second | | First | | | Second | | First | | Second | |
| First | 85% | | | 0% | | 86% | | | 0% | | 85% | | | 0% | | 88% | | | 0% | | 76% | | 4% | |
| Second | 1% | | | 14% | | 0% | | | 14% | | 0% | | | 15% | | 1% | | | 11% | | 0% | | 20% | |
| Kappa | 0.95 (0.89, 1.00)* | | | | | 1.00 | | | | | 0.98 (0.94, 1.00)* | | | | | 0.95 (0.88, 1.00)* | | | | | 0.89 (0.81, 0.97)* | | | |
| *Extent of between-trial variance*^3^ | | | | | | | | | | | | | | | | | | | | | | | | |
| With | LO | MO | | | LA | LO | MO | | | LA | LO | MO | | | LA | LO | MO | | | LA | LO | MO | | LA |
| Low | 59% | 10% | | | 0% | 62% | 0% | | | 0% | 59% | 10% | | | 0% | 62% | 0% | | | 0% | 69% | 3% | | 0% |
| Moderate | 0% | 10% | | | 0% | 0% | 24% | | | 0% | 0% | 10% | | | 0% | 0% | 24% | | | 0% | 0% | 7% | | 0% |
| Large | 0% | 0% | | | 21% | 0% | 0% | | | 14% | 0% | 0% | | | 21% | 0% | 0% | | | 14% | 0% | 0% | | 21% |
| Kappa | 0.80 (0.60, 1.00)^†^ | | | | | 1.00 | | | | | 0.80 (0.60, 1.00)^†^ | | | | | 1.00 | | | | | 0.92 (0.78, 1.00)^†^ | | | |
| **Inconsistency factor (log odd ratio)** | | | | | | | | | | | | | | | | | | | | | | | | |
|  | MAR w/o | | | | | MME w/o | | | | | MMNE w/o | | | | | BC w/o | | | | | WC w/o | | | |
| *Strength of evidence*^1^ | | | | | | | | | | | | | | | | | | | | | | | | |
| With | Weak | | Strong | | | Weak | | Strong | | | Weak | | Strong | | | Weak | | Strong | | | Weak | | Strong | |
| Weak | 94% | | 0% | | | 94% | | 0% | | | 94% | | 0% | | | 94% | | 0% | | | 95% | | 0% | |
| Strong | 0% | | 6% | | | 0% | | 6% | | | 0% | | 6% | | | 1% | | 5% | | | 0% | | 5% | |
| Kappa | 1.00 | | | | | 1.00 | | | | | 1.00 | | | | | 0.93 (0.79, 1.00)* | | | | | 1.00 | | | |
| *Direction of evidence*^2^ | | | | | | | | | | | | | | | | | | | | | | | | |
| With | Positive | | Negative | | | Positive | | Negative | | | Positive | | Negative | | | Positive | | Negative | | | Positive | | Negative | |
| Positive | 64% | | 0% | | | 46% | | 1% | | | 47% | | 1% | | | 46% | | 1% | | | 46% | | 1% | |
| Negative | 1% | | 52% | | | 1% | | 51% | | | 1% | | 51% | | | 1% | | 51% | | | 1% | | 51% | |
| Kappa | 0.97 (0.93, 1.00)* | | | | | 0.96 (0.91, 1.00)* | | | | | 0.97 (0.93, 1.00)* | | | | | 0.96 (0.91, 1.00)* | | | | | 0.96 (0.91, 1.00)* | | | |

BC: best-case scenario; LA: large; LO: low; MAR: missing at random; MME: more missing cases are event in all interventions; MMNE: more missing cases are non-events in all interventions; MO: moderate; WC: worst-case scenario; w/o: without.

^1^Strong evidence when 0 (in the log scale) is not included in the 95% credible interval, otherwise weak evidence.

^2^Whether the estimated log odds ratio favors the first or second intervention in the respective basic parameter. In case of inconsistency factor, whether the difference between direct and indirect estimate for a specific comparison is positive or negative.

^3^Estimated between-trial variance is low, moderate and large when it is smaller than the median, between the median and 3^rd^ quartile or larger than the 3^rd^ quartile, respectively, of the selected empirical distribution for the true between-trial variance.

*Almost perfect agreement (0.81 – 1.00); ^†^95% confidence interval is too wide to judge the level of agreement with confidence.

| **Agreement between identical and hierarchical structure of log IMOR** |
| --- |

**Table S6. Agreement on direction, strength of evidence and extent of heterogeneity**

| **Basic parameters (log odds ratio)** | | | | | | | | | | | | | | |
| --- | --- | --- | --- | --- | --- | --- | --- | --- | --- | --- | --- | --- | --- | --- |
|  | Common-within-network | | | | | | Trial-specific | | | | Intervention-specific | | | |
| *Strength of evidence*^1^ | | | | | | | | | | | | | | |
| IDE | Weak | | | | Strong | | Weak | | Strong | | Weak | | Strong | |
| Weak | 57% | | | | 1% | | 56% | | 0% | | 60% | | 1% | |
| Strong | 2% | | | | 41% | | 2% | | 42% | | 1% | | 38% | |
| Kappa | 0.95 (0.91, 1.00)* | | | | | | 0.95 (0.91, 1.00)* | | | | 0.96 (0.93, 1.00)* | | | |
| *Direction of evidence*^2^ | | | | | | | | | | | | | | |
| IDE | | First | | | Second | | First | | Second | | First | | Second | |
| First | | 86% | | | 0% | | 86% | | 0% | | 85% | | 1% | |
| Second | | 0% | | | 14% | | 0% | | 14% | | 0% | | 14% | |
| Kappa | | 1.00 | | | | | 1.00 | | | | 0.98 (0.93, 1.00)* | | | |
| *Extent of between-trial variance*^3^ | | | | | | | | | | | | | | |
| IDE | | LO | MO | | | LA | LO | MO | | LA | LO | MO | | LA |
| Low | | 62% | 0% | | | 0% | 62% | 0% | | 0% | 66% | 0% | | 0% |
| Moderate | | 10% | 7% | | | 0% | 14% | 3% | | 0% | 7% | 7% | | 3% |
| Large | | 0% | 0% | | | 21% | 0% | 7% | | 14% | 0% | 3% | | 14% |
| Kappa | | 0.79 (0.57, 1.00)^†^ | | | | | 0.57 (0.27, 0.88)^†^ | | | | 0.71 (0.45, 0.97)^†^ | | | |
| **Inconsistency factor (log odd ratio)** | | | | | | | | | | | | | | |
|  | | Common-within-network | | | | | Trial-specific | | | | Intervention-specific | | | |
| *Strength of evidence*^1^ | | | | | | | | | | | | | | |
| IDE | | Weak | | Strong | | | Weak | | Strong | | Weak | | Strong | |
| Weak | | 94% | | 0% | | | 94% | | 0% | | 94% | | 1% | |
| Strong | | 0% | | 6% | | | 0% | | 6% | | 1% | | 4% | |
| Kappa | | 1.00 | | | | | 1.00 | | | | 0.85 (0.64, 1.00)^†^ | | | |
| *Direction of evidence*^2^ | | | | | | | | | | | | | | |
| IDE | | Positive | | Negative | | | Positive | | Negative | | Positive | | Negative | |
| Positive | | 48% | | 1% | | | 46% | | 0% | | 47% | | 1% | |
| Negative | | 0% | | 51% | | | 0% | | 54% | | 0% | | 52% | |
| Kappa | | 0.97 (0.93, 1.00)* | | | | | 1.00 | | | | 0.98 (0.96, 1.00)* | | | |

IDE: identical; LA: large; LO: low; MO: moderate.

^1^Strong evidence when 0 (in the log scale) is not included in the 95% credible interval, otherwise weak evidence.

^2^Whether the estimated log odds ratio favors the first or second intervention in a comparison. In case of inconsistency factor, whether the difference between direct and indirect estimate for a specific comparison is positive or negative.

^3^Estimated between-trial variance is low, moderate and large when it is smaller than the median, between the median and 3^rd^ quartile and larger than the 3^rd^ quartile, respectively, of the selected empirical distribution for the true between-trial variance.

*Almost perfect agreement (0.81 – 1.00);

^†^95% confidence interval is too wide to judge the level of agreement with confidence.

| **Agreement among different structures of prior distribution on log IMOR** |
| --- |

**Identical structure**

**Table S7. Agreement on direction, strength of evidence and extent of heterogeneity**

| **Basic parameters (log odds ratio)** | | | | | | | | | | | | | | |
| --- | --- | --- | --- | --- | --- | --- | --- | --- | --- | --- | --- | --- | --- | --- |
|  | Common *vs*. Intervention | | | | | | Common *vs*. Trial | | | | Intervention *vs*. Trial | | | |
| *Strength of evidence*^1^ | | | | | | | | | | | | | | |
|  | Weak | | | | Strong | | Weak | | Strong | | Weak | | Strong | |
| Weak | 57% | | | | 0% | | 55% | | 2% | | 55% | | 6% | |
| Strong | 4% | | | | 39% | | 1% | | 42% | | 1% | | 38% | |
| Kappa | 0.91 (0.85, 0.97)* | | | | | | 0.95 (0.91, 1.00)* | | | | 0.86 (0.79, 0.94)* | | | |
| *Direction of evidence*^2^ | | | | | | | | | | | | | | |
|  | | First | | | Second | | First | | Second | | First | | Second | |
| First | | 86% | | | 1% | | 86% | | 0% | | 86% | | 0% | |
| Second | | 0% | | | 14% | | 0% | | 14% | | 1% | | 14% | |
| Kappa | | 0.98 (0.93, 1.00)* | | | | | 1.00 | | | | 0.98 (0.93, 1.00)* | | | |
| *Extent of between-trial variance*^3^ | | | | | | | | | | | | | | |
|  | | LO | MO | | | LA | LO | MO | | LA | LO | MO | | LA |
| Low | | 62% | 0% | | | 0% | 62% | 0% | | 0% | 62% | 3% | | 0% |
| Moderate | | 3% | 14% | | | 0% | 0% | 17% | | 0% | 0% | 14% | | 3% |
| Large | | 0% | 3% | | | 17% | 0% | 0% | | 21% | 0% | 0% | | 17% |
| Kappa | | 0.87 (0.69, 1.00)^†^ | | | | | 1.00 | | | | 0.87 (0.69, 1.00)^†^ | | | |
| **Inconsistency factor (log odd ratio)** | | | | | | | | | | | | | | |
|  | | Common *vs*. Intervention | | | | | Common *vs*. Trial | | | | Intervention *vs*. Trial | | | |
| *Strength of evidence*^1^ | | | | | | | | | | | | | | |
|  | | Weak | | Strong | | | Weak | | Strong | | Weak | | Strong | |
| Weak | | 94% | | 0% | | | 94% | | 0% | | 94% | | 1% | |
| Strong | | 1% | | 5% | | | 0% | | 6% | | 0% | | 5% | |
| Kappa | | 0.93 (0.79, 1.00)* | | | | | 1.00 | | | | 0.93 (0.79, 1.00)* | | | |
| *Direction of evidence*^2^ | | | | | | | | | | | | | | |
|  | | Positive | | Negative | | | Positive | | Negative | | Positive | | Negative | |
| Positive | | 48% | | 1% | | | 46% | | 2% | | 46% | | 1% | |
| Negative | | 0% | | 51% | | | 0% | | 51% | | 0% | | 52% | |
| Kappa | | 0.98 (0.96, 1.00)* | | | | | 0.96 (0.91, 1.00)* | | | | 0.97 (0.93, 1.00)* | | | |

Common: common-within-network; Intervention: intervention-specific; LA: large; LO: low; MO: moderate; Trial: trial-specific.

^1^Strong evidence when 0 (in the log scale) is not included in the 95% credible interval, otherwise weak evidence.

^2^Whether the estimated log odds ratio favors the first or second intervention in a comparison. In case of inconsistency factor, whether the difference between direct and indirect estimate for a specific comparison is positive or negative.

^3^Estimated between-trial variance is low, moderate and large when it is smaller than the median, between the median and 3^rd^ quartile and larger than the 3^rd^ quartile, respectively, of the selected empirical distribution for the true between-trial variance.

*Almost perfect agreement (0.81 – 1.00);

^†^95% confidence interval is too wide to judge the level of agreement with confidence.

**Hierarchical structure**

**Table S8. Agreement on direction, strength of evidence and extent of heterogeneity**

| **Basic parameters (log odds ratio)** | | | | | | | | | | | | | | |
| --- | --- | --- | --- | --- | --- | --- | --- | --- | --- | --- | --- | --- | --- | --- |
|  | Common *vs*. Intervention | | | | | | Common *vs*. Trial | | | | Intervention *vs*. Trial | | | |
| *Strength of evidence*^1^ | | | | | | | | | | | | | | |
|  | Weak | | | | Strong | | Weak | | Strong | | Weak | | Strong | |
| Weak | 58% | | | | 0% | | 57% | | 1% | | 58% | | 3% | |
| Strong | 3% | | | | 39% | | 1% | | 41% | | 1% | | 39% | |
| Kappa | 0.94 (0.89, 0.99)* | | | | | | 0.95 (0.91, 1.00)* | | | | 0.92 (0.86 0.98)* | | | |
| *Direction of evidence*^2^ | | | | | | | | | | | | | | |
|  | | First | | | Second | | First | | Second | | First | | Second | |
| First | | 85% | | | 1% | | 86% | | 0% | | 85% | | 0% | |
| Second | | 0% | | | 14% | | 0% | | 14% | | 1% | | 14% | |
| Kappa | | 0.95 (0.89, 1.00)* | | | | | 1.00 | | | | 0.95 (0.89, 1.00)* | | | |
| *Extent of between-trial variance*^3^ | | | | | | | | | | | | | | |
|  | | LO | MO | | | LA | LO | MO | | LA | LO | MO | | LA |
| Low | | 72% | 0% | | | 0% | 72% | 0% | | 0% | 72% | 0% | | 0% |
| Moderate | | 0% | 7% | | | 0% | 3% | 3% | | 0% | 3% | 7% | | 0% |
| Large | | 0% | 3% | | | 17% | 0% | 7% | | 14% | 0% | 3% | | 14% |
| Kappa | | 0.92 (0.77, 1.00)^†^ | | | | | 0.75 (0.48, 1.00)^†^ | | | | 0.75 (0.48, 1.00)^†^ | | | |
| **Inconsistency factor (log odd ratio)** | | | | | | | | | | | | | | |
|  | | Common *vs*. Intervention | | | | | Common *vs*. Trial | | | | Intervention *vs*. Trial | | | |
| *Strength of evidence*^1^ | | | | | | | | | | | | | | |
|  | | Weak | | Strong | | | Weak | | Strong | | Weak | | Strong | |
| Weak | | 94% | | 0% | | | 94% | | 0% | | 94% | | 1% | |
| Strong | | 1% | | 5% | | | 0% | | 6% | | 0% | | 5% | |
| Kappa | | 0.93 (0.79, 1.00)* | | | | | 1.00 | | | | 0.93 (0.79, 1.00)* | | | |
| *Direction of evidence*^2^ | | | | | | | | | | | | | | |
|  | | Positive | | Negative | | | Positive | | Negative | | Positive | | Negative | |
| Positive | | 47% | | 0% | | | 46% | | 1% | | 46% | | 1% | |
| Negative | | 0% | | 53% | | | 0% | | 53% | | 0% | | 53% | |
| Kappa | | 1.00 | | | | | 0.98 (0.96, 1.00)* | | | | 0.98 (0.96, 1.00)* | | | |

Common: common-within-network; Intervention: intervention-specific; LA: large; LO: low; MO: moderate; Trial: trial-specific.

^2^Whether the estimated log odds ratio favors the first or second intervention in a comparison. In case of inconsistency factor, whether the difference between direct and indirect estimate for a specific comparison is positive or negative.

^3^Estimated between-trial variance is low, moderate and large when it is smaller than the median, between the median and 3^rd^ quartile and larger than the 3^rd^ quartile, respectively, of the selected empirical distribution for the true between-trial variance.

*Almost perfect agreement (0.81 – 1.00);

^†^95% confidence interval is too wide to judge the level of agreement with confidence.

| **Agreement between pattern-mixture model and selection model** |
| --- |

**Table S9. Agreement on direction, strength of evidence and extent of heterogeneity**

| **Basic parameters (log odds ratio)** | | | | | | | | | | | | | | |
| --- | --- | --- | --- | --- | --- | --- | --- | --- | --- | --- | --- | --- | --- | --- |
|  | Common within network | | | | | | Trial-specific | | | | Intervention-specific | | | |
| *Strength of evidence*^1^ | | | | | | | | | | | | | | |
| Pattern | Weak | | | | Strong | | Weak | | Strong | | Weak | | Strong | |
| Weak | 56% | | | | 1% | | 55% | | 1% | | 61% | | 1% | |
| Strong | 1% | | | | 42% | | 1% | | 43% | | 1% | | 38% | |
| Kappa | 0.96 (0.91, 1.00)* | | | | | | 0.97 (0.93, 1.00)* | | | | 0.96 (0.93, 1.00)* | | | |
| *Direction of evidence*^2^ | | | | | | | | | | | | | | |
| Pattern | | First | | | Second | | First | | Second | | First | | Second | |
| First | | 85% | | | 1% | | 86% | | 1% | | 85% | | 1% | |
| Second | | 0% | | | 14% | | 0% | | 14% | | 0% | | 14% | |
| Kappa | | 0.95 (0.89, 1.00)* | | | | | 0.98 (0.93, 1.00)* | | | | 0.98 (0.95, 1.00)* | | | |
| *Extent of between-trial variance*^3^ | | | | | | | | | | | | | | |
| Pattern | | LO | MO | | | LA | LO | MO | | LA | LO | MO | | LA |
| Low | | 59% | 3% | | | 0% | 62% | 0% | | 0% | 66% | 0% | | 0% |
| Moderate | | 3% | 14% | | | 0% | 3% | 14% | | 0% | 3% | 10% | | 3% |
| Large | | 0% | 3% | | | 17% | 0% | 0% | | 21% | 0% | 0% | | 17% |
| Kappa | | 0.81 (0.60, 1.00)^†^ | | | | | 0.93 (0.81, 1.00)* | | | | 0.86 (0.67, 1.00)^†^ | | | |
| **Inconsistency factor (log odd ratio)** | | | | | | | | | | | | | | |
|  | | Common within network | | | | | Trial-specific | | | | Intervention-specific | | | |
| *Strength of evidence*^1^ | | | | | | | | | | | | | | |
| Pattern | | Weak | | Strong | | | Weak | | Strong | | Weak | | Strong | |
| Weak | | 94% | | 0% | | | 94% | | 0% | | 94% | | 1% | |
| Strong | | 1% | | 5% | | | 0% | | 6% | | 0% | | 5% | |
| Kappa | | 0.93 (0.79, 1.00)* | | | | | 1.00 | | | | 0.87 (0.68, 1.00)^†^ | | | |
| *Direction of evidence*^2^ | | | | | | | | | | | | | | |
| Pattern | | Positive | | Negative | | | Positive | | Negative | | Positive | | Negative | |
| Positive | | 49% | | 0% | | | 46% | | 0% | | 48% | | 0% | |
| Negative | | 0% | | 51% | | | 1% | | 53% | | 1% | | 51% | |
| Kappa | | 1.00 | | | | | 0.98 (0.96, 1.00)* | | | | 0.98 (0.96, 1.00)* | | | |

Common: common-within-network; Intervention: intervention-specific; LA: large; LO: low; MO: moderate; Trial: trial-specific.

^1^Strong evidence when 0 (in the log scale) is not included in the 95% credible interval, otherwise weak evidence.

^2^Whether the estimated log odds ratio favors the first or second intervention in a comparison. In case of inconsistency factor, whether the difference between direct and indirect estimate for a specific comparison is positive or negative.

^3^Estimated between-trial variance is low, moderate and large when it is smaller than the median, between the median and 3^rd^ quartile and larger than the 3^rd^ quartile, respectively, of the selected empirical distribution for the true between-trial variance.

*Almost perfect agreement (0.81 – 1.00);

^†^95% confidence interval is too wide to judge the level of agreement with confidence.

| **Agreement between moderate and other prior variances for log IMOR** |
| --- |

**Table S10. Agreement on direction, strength of evidence and extent of heterogeneity**

| **Basic parameters (log odds ratio)** | | | | | | | | | |
| --- | --- | --- | --- | --- | --- | --- | --- | --- | --- |
|  | Conservative | | | | | Liberal | | | |
| *Strength of evidence*^1^ | | | | | | | | | |
| Moderate | Weak | | | Strong | | Weak | | Strong | |
| Weak | 61% | | | 0% | | 60% | | 2% | |
| Strong | 2% | | | 37% | | 1% | | 38% | |
| Kappa | 0.96 (0.93, 1.00)* | | | | | 0.95 (0.91, 1.00)* | | | |
| *Direction of evidence*^2^ | | | | | | | | | |
| Moderate | First | | | Second | | First | | Second | |
| First | 84% | | | 2% | | 86% | | 0% | |
| Second | 0% | | | 14% | | 1% | | 14% | |
| Kappa | 0.93 (0.86, 1.00)* | | | | | 0.98 (0.93, 1.00)* | | | |
| *Extent of between-trial variance*^3^ | | | | | | | | | |
| Moderate | LO | MO | | | LA | LO | MO | | LA |
| Low | 62% | 3% | | | 0% | 62% | 3% | | 0% |
| Moderate | 3% | 14% | | | 0% | 3% | 14% | | 3% |
| Large | 0% | 3% | | | 14% | 0% | 0% | | 17% |
| Kappa | 0.80 (0.58, 1.00)^†^ | | | | | 0.87 (0.69, 1.00)^†^ | | | |
| **Inconsistency factor (log odd ratio)** | | | | | | | | | |
|  | Conservative | | | | | Liberal | | | |
| *Strength of evidence*^1^ | | | | | | | | | |
| Moderate | Weak | | Strong | | | Weak | | Strong | |
| Weak | 94% | | 1% | | | 94% | | 1% | |
| Strong | 1% | | 4% | | | 0% | | 5% | |
| Kappa | 0.70 (0.41, 0.99)^†^ | | | | | 0.93 (0.79, 1.00)* | | | |
| *Direction of evidence*^2^ | | | | | | | | | |
| Moderate | Positive | | Negative | | | Positive | | Negative | |
| Positive | 47% | | 1% | | | 48% | | 0% | |
| Negative | 0% | | 52% | | | 1% | | 51% | |
| Kappa | 0.98 (0.96, 1.00)* | | | | | 0.99 (0.96, 1.00)* | | | |

Conservative: variance equal 4; LA: large; Liberal: variance equal 0.25; LO: low; MO: moderate; Moderate: variance equal 1.

^1^Strong evidence when 0 (in the log scale) is not included in the 95% credible interval, otherwise weak evidence.

^2^Whether the estimated log odds ratio favors the first or second intervention in a comparison. In case of inconsistency factor, whether the difference between direct and indirect estimate for a specific comparison is positive or negative.

^3^Estimated between-trial variance is low, moderate and large when it is smaller than the median, between the median and 3^rd^ quartile and larger than the 3^rd^ quartile, respectively, of the selected empirical distribution for the true between-trial variance.

*Almost perfect agreement (0.81 – 1.00);

^†^95% confidence interval is too wide to judge the level of agreement with confidence.

| **Judging the extraction accuracy of the analySed networks** |
| --- |

**Table S11. Judgment of accuracy extraction of the eligible networks with justifications**

| ID | Information on outcome of completers for all included trials | Information on outcome “withdrawal” for all included trials | Explicit description of how missing outcome data are handled in NMA | Extraction accuracy | Notes |
| --- | --- | --- | --- | --- | --- |
| 1 |  | x | x | Unclear | LOCF has been already employed in some of the trials. |
| 2 |  | x |  | Unacceptable | In Table 1, in some trials the analysed for Response is equal to or smaller than the analysed for Withdrawals. Outcomes might have been extracted and analysed as reported. |
| 3 |  | x |  | Unacceptable | The reviewers excluded trials without ITT in a sensitivity analysis. Therefore, they might have analysed the outcomes as reported in the trials |
| 4 |  | x |  | Unacceptable | 'Analyses were carried out for the intention to treat (ITT) '. Results are provided narratively without tabulation of the study results in order to understand how many were analysed out of the total randomised. |
| 5 |  | x |  | Unacceptable | In Table 1, totals under Efficacy and Acceptability are the same in some trials but smaller in the former in other trials. Probably the reviewers extracted the data as reported in trials. |
| 6 |  | x |  | Unacceptable | In Table 2, number analysed is smaller than randomised (NPts FAS) in same trials; No information on how each trials handled MOD; no information on how the reviewers handled MOD. |
| 7 |  | x |  | Unacceptable | By comparing Figure 1 with Figure 2 and account for the information on ITT/PP in Table S1, some trials did ITT (no further information) and other analysed as ACA. |
| 8 |  | x |  | Unacceptable | By comparing the totals in the efficacy outcome with those in the DO outcome, we might infer that ITT with imputation has been done; The reviewers reported that they planned ITT. |
| 9 |  | x | x | Unclear | 'We observe that, for all but EOP 1003 and EOP 1004 studies, one important limitation was that we could extract data reported with the Last Observation Carried Forward (LOCF) technique to account for missing data, although we attempted an available case extraction from material on FDA website yielding incomplete data collection.' Table 1 presents the sample analysed and the MOD frequency per arm. |
| 10 |  | x |  | Unacceptable | The reviewers report that they employed ITT but without further information; It is has been explicitly reported that each trial applied ITT. |
| 11 |  | x | x | Unclear | The reviewers explicitly reported than ITT was applied and by comparing totals reported in the Table of Characteristics with those in Analysis 1.1, we conclude that ITT might have been indeed applied |
| 12 |  | x |  | Unacceptable | Table 1 reports ITT, but the totals in Figure 2 (primary outcome) are smaller than the totals in Figure 3 (DO) for some trials. Probably outcomes extracted (and analysed) as reported in the trials. |
| 13 |  | x |  | Unacceptable | By comparing the totals in the dropout outcome (Figure 2C) with the totals in the primary outcome (Figure 2A), the latter are smaller. Probably ACA was employed. |
| 14 |  | x |  | Unacceptable | In Appendix Table S1, by comparing the total of the primary outcome (response) with the total in the DO, we see that some trials may have employed ITT whereas others ACA. Probably outcomes extracted (and analysed) as reported in the trials. |
| 15 |  | x |  | Unacceptable | Table 1 refers to Q6 'Were all analyses carried out using data from the Intention To Treat (ITT) patient group?'. Probably outcomes extracted (and analysed) as reported in the trials. |
| 16 |  | x |  | Unacceptable | Compare Appendix B (it gives information on randomised and completers’ sample) with Table 1: totals in Table 1 are smaller in all studies. Probably ACA was employed. |
| 17 |  | x |  | Unacceptable | No distinction on the analysed and randomised sample for each trial; no information on how MOD have been handled in each trial. |
| 18 |  | x | x | Unclear | Table e4 (information on number randomised) agrees with Table e2 (efficacy outcome) and Table e4 (acceptability) in terms of total analysed but no distinction is made between completers and MOD. Imputation under AMF scenario. |
| 19 |  | x |  | Unacceptable | There is explicit information on Supplemental Table 6 on the statistical method employed in the trials but the reviewers do not reported how they handled MOD. |
| 20 |  | x |  | Unacceptable | Table S1 provides information on the analysed sample for the primary outcome and the DO. These samples are the same in some trials, whereas smaller in the former in others. Possible outcome extracted and analysed as reported in the trials. |
| 21 |  | x |  | Unacceptable | No distinction on the analysed and randomised sample for each trial; no information on how MOD have been handled in each trial; reviewers don’t report how they planned to handle MOD. |
| 22 |  | x |  | Unacceptable | Number randomised has been also analysed, by comparing the Table of characteristics and the information on attrition bias with the analysed outcome. The reviewers do not mention how they handled MOD. |
| 23 |  | x |  | Unacceptable | Table 1 explicitly reports how each trial handled MOD for every outcome. Reviewers do not mention how they handled MOD. |
| 24 |  | x | x | Unclear | 'The denominator used in all trials was based on a modified intention-to-treat (mITT) analysis […]'. In Table 1 many studies provide data both in ITT and mITT and others as ITT only. |
| 25 |  | x | x | Unclear | By comparing the totals in the efficacy outcome (Figure S1) with those in the DO (Figure S2), it seems that ITT must have been employed. Imputation under AMF scenario. |
| 26 |  | x |  | Unacceptable | There is information on the analyses and randomised sample for each arm of every trial ('Results – Key information about each identified RCT' in Supplementary material); no information on how MOD were handled. |
| 27 |  | x |  | Unacceptable | By comparing the analyses totals (Analysis 1, Comparison 1) with the randomised total in RoB table it is obvious that the reviewers applied genuine ITT, but without information on how reviewers handled MOD. |
| 28 |  | x |  | Unacceptable | No distinction between randomised and analysed sample; no information on how MOD were addressed. |
| 29 |  | x |  | Unacceptable | The reviewers analysed the data as reported ''All studies considered non-completion as failure and reported data for the randomised patients who received at least 1 dose (ie, intent-to-treat exposed population), except for the study by Riddler et al, which ignored missing data''. Combination of ITT, modified-TT and ACA. |

References of the analysed reviews are provided below e-Table2
