# Supplementary material for: An empirical comparison of Bayesian modelling strategies for missing binary outcome data in network meta-analysis
Source: BMC Med Res Methodol. 2019 Apr 24;19:86. doi: 10.1186/s12874-019-0731-y (PMC6480793; doi:10.1186/s12874-019-0731-y)
Supplement: Supplementary file 2 — Supplementary information of the Methods. (DOCX 184 kb) [file 12874_2019_731_MOESM2_ESM.docx]

**Additional file 2. Supplementary information of the Methods**

| **Characterising networks based on prevalence and balance of missingness** |
| --- |

**Prevalence of total missingness**

For each network, we compared the median of the total percentage MOD (%MOD) across the included trials with the ‘five-and-twenty rule’ as proposed by Sackett et al. [1] and we considered missingness to be low for median up to 5% (low attrition bias risk), moderate for median between 5% and 20% (moderate attrition bias risk) and large for median above 20% (large attrition bias risk).

**Balance of missingness**

To consider a trial as having or not balanced MOD in the compared interventions, we tested the null hypothesis that the %MOD is the same between the compared interventions using a two-sided Pearson's chi-squared test statistic and significance level 5%. Then, we calculated the difference in %MOD between the compared interventions in each trial in order to draw the kernel density in each group (i.e. trials with balance versus trials with imbalance in MOD according to the test statistic). In case of multi-arm trials, we calculated the difference between maximum and minimum %MOD. The intersection point of the densities was selected to be the threshold of balance (see, Figure below). Then, for each network, we compared this threshold (equal to 6.5%) with the median of the difference in %MOD between the compared interventions across the included trials: networks with median larger than 6.5% were considered to have imbalance in MOD.


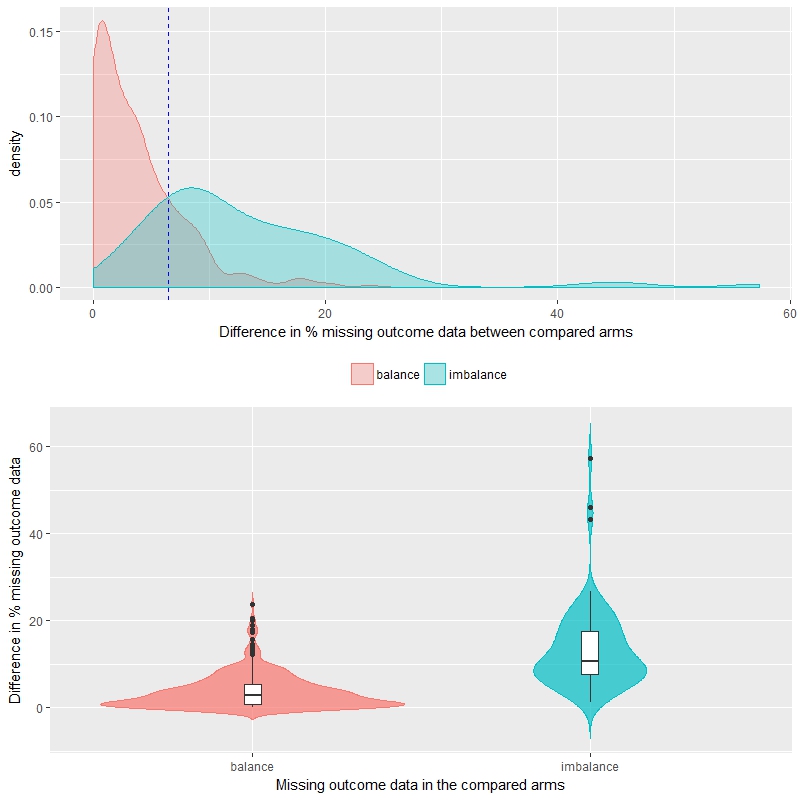


**6.5 *(threshold)***

**Figure above.** Kernel density of trials with balance (red density) versus imbalance (blue density) in %MOD between the compared interventions.

**Figure below.** Violin plot (with integrated boxplot) on the distribution of difference in %MOD between the compared interventions in trials with balance (red violin) versus trials with imbalance (blue violin) in missingness.

| **Node-splitting approach and ranking statistics** |
| --- |

**Node-splitting approach**

The node-splitting approach has been proposed by Dias et al. [1] within the Bayesian framework to assess possible inconsistency locally while using the whole network to obtain an indirect estimate for a comparison of a closed-loop. The idea behind the node-splitting approach is the following: a comparison of a closed-loop is isolated (split) and random-effects meta-analysis is performed for that comparison, whereas the remaining network is used to estimate an indirect estimate for the split comparison. The difference between direct and indirect estimate for that comparison is known as inconsistency factor (IF). A large posterior probability of IF being different from zero (e.g. above 95%) provides sufficient evidence that inconsistency may be present. To improve the estimation of $\tau^{2}$, Dias et al. [1] recommended using a common $\tau^{2}$ for both meta-analysis and NMA model (after removing the trials of the split comparison). van Valkenhoef et al. [2] refined further the node-splitting approach by proposing a decision rule to automatically select the comparisons to split and they incorporated their method in the R package *gemtc* ([http://cran.r-project.org/ package=gemtc](http://cran.r-project.org/%20package=gemtc)) [3].

**Measures of intervention hierarchy**

To provide a hierarchy of the investigated interventions for a specific outcome, various measures have been proposed that can be obtained straightforward using Bayesian approaches [4]. Ranking probabilities indicate the probability that an intervention will be placed in a specific ranking (e.g. placebo has 1% chance to be the best, but 96% chance to be the last in the hierarchy) and they can be plotted against all rankings for each intervention to obtain the so-called rankograms [4]. Rankograms reflect the uncertainty about the hierarchy of each intervention. Another measure of intervention hierarchy is the estimation of the surface under the cumulative ranking curve (SUCRA) for each intervention [4]. SUCRA reflects the percentage of potency (measured as effectiveness or safety) an intervention achieves compared to an imaginary intervention that ranks first with certainty [5].

**References**

1. Dias S, Welton NJ, Caldwell DM, Ades AE. Checking consistency in mixed treatment comparison meta-analysis. Stat Med. 2010;29:932-44.
2. van Valkenhoef G, Dias S, Ades AE, Welton NJ. Automated generation of node-splitting models for assessment of inconsistency in network meta-analysis. Res Synth Methods. 2016;7:80-93.
3. van Valkenhoef G, Kuiper J. gemtc: Network meta-analysis using Bayesian methods. R package version 0.8-2. 2016. <http://github.com/gertvv/gemtc>.
4. Salanti G, Ades AE, Ioannidis JP. Graphical methods and numerical summaries for presenting results from multiple-treatment meta-analysis: an overview and tutorial. J Clin Epidemiol. 2011;64:163-71.
5. Mavridis D, Giannatsi M, Cipriani A, Salanti G. A primer on network meta-analysis with emphasis on mental health. Evid Based Ment Health. 2015;18:40-6.

| **Information on model estimation** |
| --- |

**Network meta-analysis model**

Bayesian random-effects network meta-analysis (NMA) models with consistency equations and accountability of multi-arm trials were fitted using JAGS via the R package *R2jags* [2]. Specifically, we extended the NMA model developed by Dias et al. [3] in order to incorporate the studied missingness parameter following the parameterization of the underlying event probability described in Turner et al. [4]. Three parallel chains of different initial values were used for 10,000 updates and a burn-in of 1,000 Markov chain Monte Carlo samples [5]. Convergence assessment was based on Gelman–Rubin convergence diagnostic, $\hat{R}$ [6]. From our analyses, we excluded results on parameters that corresponded to Gelman–Rubin $\hat{R}$ larger than 1.1, as convergence for those parameters was not achieved, and by extent, the corresponding posterior distributions could not be trusted.

**Specification of prior distributions**

Normal distribution with mean 0 and variance 100^2^ was assigned on location parameters (i.e. basic parameters and trial effects on the logarithmic scale), whereas empirical priors specific to outcome and intervention-comparison type studied in each network were assigned on $\tau^{2}$ (assumed common in each network) as suggested by Turner et al. [7] in order to improve estimation of the parameter, especially, in case of sparse networks and/ or rare events (see, Additional file 4). In the selection model, we assigned normal prior distribution on $\gamma_{ik}$ with mean 0 and variance 1000 [8, 9]. In each network, the reference intervention was determined to be the most frequently studied intervention across the included trials.

**Node-splitting approach for local inconsistency**

Consistency was evaluated locally using the node-splitting approach only in networks with at least one closed loop that was not informed only by multi-arm trials [10, 11]. Initially, we used the R package *gemtc* [12] to identify the comparisons to split in each network and then, we inserted these comparisons in the node-splitting model developed by Dias et al. [11].

**References**

1. Sackett DL, Richardson WS, Rosenberg W, Haynes RB. Evidence-based medicine: how to practice and teach EBM. New York: Churchill Livingstone; 1997.
2. Su YS, Yajima M. R2jags: Using R to run ‘JAGS’. R package version 0.5-7. 2015. <https://CRAN.R-project.org/package=R2jags>.
3. Dias S, Sutton AJ, Ades AE, Welton NJ. Evidence synthesis for decision making 2: a generalized linear modeling framework for pairwise and network meta-analysis of randomized controlled trials. Med Decis Making. 2013;33:607–17.
4. Turner NL, Dias S, Ades AE, Welton NJ. A Bayesian framework to account for uncertainty due to missing binary outcome data in pairwise meta-analysis. Stat Med. 2015;34:2062-80.
5. Lunn DJ, Thomas A, Best N, Spiegelhalter D. WinBUGS – A Bayesian modelling framework: Concepts, structure, and extensibility. Stat Comput. 2000;10:325–37.
6. Gelman A, Rubin DB. Inference from iterative simulation using multiple sequences. Stat Sci. 1992;7:457–72.
7. Turner RM, Jackson D, Wei Y, Thompson SG, Higgins JP. Predictive distributions for between-study heterogeneity and simple methods for their application in Bayesian meta-analysis. Stat Med. 2015;34:984-98.
8. White IR, Welton NJ, Wood AM, Ades AE, Higgins JP. Allowing for uncertainty due to missing data in meta-analysis--part 2: hierarchical models. Stat Med. 2008;27:728-45.
9. Spineli LM, Higgins JP, Cipriani A, Leucht S, Salanti G. Evaluating the impact of imputations for missing participant outcome data in a network meta-analysis. Clin Trials. 2013;10:378-88.
10. van Valkenhoef G, Dias S, Ades AE, Welton NJ. Automated generation of node-splitting models for assessment of inconsistency in network meta-analysis. Res Synth Methods. 2016;7:80-93.
11. Dias S, Welton NJ, Caldwell DM, Ades AE. Checking consistency in mixed treatment comparison meta-analysis. Stat Med. 2010;29:932-44.
12. van Valkenhoef G, Kuiper J. gemtc: Network meta-analysis using Bayesian methods. R package version 0.8-2. 2016. <http://github.com/gertvv/gemtc>.
