# Supplementary material for: An empirical comparison of Bayesian modelling strategies for missing binary outcome data in network meta-analysis
Source: BMC Med Res Methodol. 2019 Apr 24;19:86. doi: 10.1186/s12874-019-0731-y (PMC6480793; doi:10.1186/s12874-019-0731-y)
Supplement: Supplementary file 3 — Code for all network meta-analysis models. (DOCX 41 kb) [file 12874_2019_731_MOESM3_ESM.docx]

**Additional file 3. Code for all network meta-analysis models**

######################################################################################

# #

# **Bayesian random-effects network meta-analysis** with consistency equation #

# and accommodation of multi-arm trials (Dias et al., 2013 – PMID: 23104435) #

# **Pattern-mixture model** with incorporation of log IMOR #

# (on average MAR and moderate prior variance) #

# (Turner et al., 2015 – PMID: 25809313) #

# #

######################################################################################

**## INPUT PARAMETERS**

**# ns: number of trials in the network**

**# nt: number of interventions in the network**

**# r[i, k]: number of events observed in arm k of trial i**

**# m[i, k]: number of missing participants in arm k of trial i**

**# t[i, k]: intervention in arm k of trial i**

**# obs[i, k]: number of completers in arm k of trial i**

**# n[i, k]: number randomized in arm k of trial i**

**# na[i]: number of arms in trial i**

**# ref: selected reference intervention in the network**

**# mean.tausq: mean value of the selected log-normal empirical distribution for tausq**

**# prec.tausq: precision value of the selected log-normal empirical distribution for tausq**

model{ **# MODEL STARTS**

for(i in 1:ns){

**## Baseline log odds of event**

logit(p[i, 1]) <- u[i]

u[i] ~ dnorm(0, .0001)

w[i, 1] <- 0

for(k in 1:na[i]){

**## Observed events and likelihood per arm**

r[i, k] ~ dbin(p_o[i, k], obs[i, k])

obs[i, k] <- n[i, k] - m[i, k]

p_o[i, k] <- max(0, min(1, ((-((q[i, k] - p[i, k])*(1 - IMOR[i, k]) - 1) –

sqrt((pow(((q[i, k] - p[i, k])*(1 - IMOR[i, k]) - 1), 2)) -

((4*p[i, k])*(1 - q[i, k])*(1 - IMOR[i, k]))))/(2*(1 - q[i, k])*(1 - IMOR[i, k])))))

**## Missing participants and likelihood per arm**

m[i, k] ~ dbin(q[i, k], n[i, k])

q[i, k] ~ dunif(0, 1)

**## Structure of logIMOR**

log(IMOR[i, k]) <- **[go to TABLE]**

for(k in 2:na[i]){

logit(p[i, k]) <- u[i] + theta[i, k]

theta[i, k] ~ dnorm(md[i, k], precd[i, k])

md[i, k] <- d[t[i, k]] - d[t[i, 1]] + sw[i, k]

w[i, k] <- theta[i, k] - (d[t[i, k]] - d[t[i, 1]])

sw[i, k] <- sum(w[i, 1:(k - 1)])/(k - 1)

precd[i, k] <- 2*(k - 1)*prec/k

}

}

**## Prior specification on logIMOR**

**[go to TABLE]**

**## Basic parameters - prior distributions**

d[ref] <- 0

for(t in 1:(ref - 1)){

d[t] ~ dnorm(0, .0001)

}

for(t in (ref + 1):nt){

d[t] ~ dnorm(0, .0001)

}

**## Ranking probabilities and SUCRA values**

sorted <- rank(d[])

for(t in 1:nt){

order[t] <- nt + 1 - sorted[t]

most.effective[t] <- equals(order[t], 1)

for(l in 1:nt){

effectiveness[t, l] <- equals(order[t], l)

cumeffectiveness[t, l] <- sum(effectiveness[t, 1:l])

}

SUCRA[t] <- sum(cumeffectiveness[t, 1:(nt - 1)])/(nt - 1)

}

**## Obtain reference-specific ORs and logORs**

for(t in 1:(ref - 1)){

LOR[t] <- d[t] - d[ref]

}

for(t in (ref + 1):nt){

LOR[t] <- d[t] - d[ref]

}

**## Predictive distribution for between-trial variance**

prec <- pow(tausq, -1)

tausq ~ dlnorm(mean.tausq, prec.tausq)

} **# END OF MODEL**

######################################################################################

# #

# **Bayesian random-effects network meta-analysis** with consistency equation #

# and accommodation of multi-arm trials (Dias et al., 2013 – PMID: 23104435) #

# **Selection model** with incorporation of log IMOR #

# (on average MAR and moderate prior variance) #

# (White et al., 2008 – PMID: 17703502) #

# (Spineli et al., 2013 – PMID: 23321265) #

# #

######################################################################################

**## INPUT PARAMETERS**

**# ns: number of trials in the network**

**# nt: number of interventions in the network**

**# y[k, i, 1:3]: an array consisting of the number of events, non-events, and MOD observed in arm k of trial i**

**# t[i, k]: intervention in arm k of trial i**

**# n[i, k]: number randomized in arm k of trial i**

**# na[i]: number of arms in trial i**

**# ref: selected reference intervention in the network**

**# mean.tausq: mean value of the selected log-normal empirical distribution for tausq**

**# prec.tausq: precision value of the selected log-normal empirical distribution for tausq**

model{ **# MODEL STARTS**

for(i in 1:ns){

w[i, 1] <- 0

theta[i, t[i, 1]] <- 0

logit(p[i, t[i, 1]]) <- u[i]

u[i] ~ dnorm(0, .0001)

for(k in 1:na[i]){

y[i, k, 1:3] ~ dmulti(prob[i, t[i, k], 1:3], n[i, k])

for(j in 1:3){

prob[i, t[i, k], j] <- 0.5*(3 - j)*(2 - j)*p[i, t[i, k]]*(1 - c1[i, t[i, k]]) +

(j - 1)*(3 - j)*(1 - p[i, t[i, k]])*(1 - c0[i, t[i, k]]) +

0.5*(1 - j)*(2 - j)*(p[i, t[i, k]]*c1[i, t[i, k]] + (1 - p[i, t[i, k]])*c0[i, t[i, k]])

}

}

for(k in 2:na[i]){

logit(p[i, k]) <- u[i] + theta[i, k]

theta[i, k] ~ dnorm(md[i, k], precd[i, k])

md[i, k] <- d[t[i, k]] - d[t[i, 1]] + sw[i, k]

w[i, k] <- theta[i, k] - (d[t[i, k]] - d[t[i, 1]])

sw[i, k] <- sum(w[i, 1:(k - 1)])/(k - 1)

precd[i, k] <- 2*(k - 1)*prec/k

}

}

**## Basic parameters - prior distributions**

d[ref] <- 0

for(t in 1:(ref - 1)){

d[t] ~ dnorm(0, .0001)

}

for(t in (ref + 1):nt){

d[t] ~ dnorm(0, .0001)

}

**## Ranking probabilities and SUCRA values**

sorted <- rank(d[])

for(t in 1:nt){

order[t] <- nt + 1 - sorted[t]

most.effective[t] <- equals(order[t], 1)

for(l in 1:nt){

effectiveness[t, l] <- equals(order[t], l)

cumeffectiveness[t, l] <- sum(effectiveness[t, 1:l])

}

SUCRA[t] <- sum(cumeffectiveness[t, 1:(nt - 1)])/(nt - 1)

}

**## Obtain reference-specific logORs**

for(t in 1:(ref - 1)){

LOR[t] <- d[t] - d[ref]

}

for(t in (ref + 1):nt){

LOR[t] <- d[t] - d[ref]

}

**## Specify logIMORs and their structure**

for(i in 1:ns){

for(k in 1:na[i]){

logit(c1[i, t[i, k]]) <- gamma[i, t[i, k]] + 0.5*delta[i, t[i, k]]

logit(c0[i, t[i, k]]) <- gamma[i, t[i, k]] - 0.5*delta[i, t[i, k]]

delta[i, t[i, k]] <- **[go to TABLE]**

gamma[i, t[i, k]] ~ dnorm(0, gamma.prec)

}

}

**## Prior specifications**

gamma.prec <- 0.001

**## Predictive distribution for between-trial variance**

prec <- pow(tausq, -1)

tausq ~ dlnorm(mean.tausq, prec.tausq)

} **# END OF MODEL**

##############################################################################

# #

# **Node-splitting approach** with accommodation of multi-arm trials #

# (Dias et al., 2010 – PMID: 20213715) #

# **Pattern-mixture model** with incorporation of log IMOR #

# (on average MAR and moderate prior variance) #

# (Turner et al., 2015 – PMID: 25809313) #

# #

##############################################################################

**## INPUT PARAMETERS**

**# ns: number of trials in the network**

**# nt: number of interventions in the network**

**# r[i, k]: number of events observed in arm k of trial i**

**# mod[i, k]: number of missing participants in arm k of trial i**

**# t[i, k]: intervention in arm k of trial i**

**# n[i, k]: number randomized in arm k of trial i**

**# na[i]: number of arms in trial i**

**# ref: selected reference intervention in the network**

**# split[i]: indicates the split (1 if node to split is present) and b (baseline position)**

**# m[i, k]: indexes to sweep non-baseline arms only**

**# bi[i]: vector of baseline interventions**

**# si[i, k]: matrix of non-baseline interventions**

**# pair: node to split**

**# mean.tausq: mean value of the selected log-normal empirical distribution for tausq**

**# prec.tausq: precision value of the selected log-normal empirical distribution for tausq**

model{ **# MODEL STARTS**

for(i in 1:ns){

w[i, 1] <- 0

j[i, 1] <- 0

theta[i, bi[i]] <- 0

mu[i] ~ dnorm(0,.0001)

for(k in 1:na[i]){

**## Observed events and likelihood per arm**

obs[i, k] <- n[i, k] - mod[i, k]

r[i, k] ~ dbin(p_o[i, k], obs[i, k])

p_o[i, k] <- max(0, min(1, ((-((q[i, k] - p[i, k])*(1 - IMOR[i, k]) - 1) –

sqrt((pow(((q[i, k] - p[i, k])*(1 - IMOR[i, k]) - 1), 2)) -

((4*p[i, k])*(1 - q[i, k])*(1 - IMOR[i, k]))))/(2*(1 - q[i, k])*(1 - IMOR[i, k])))))

**## Missing participants and likelihood per arm**

mod[i, k] ~ dbin(q[i, k], n[i, k])

q[i, k] ~ dunif(0, 1)

**## Structure of logIMOR**

log(IMOR[i, k]) <- **[go to TABLE]**

logit(p[i, k]) <- mu[i] + theta[i, t[i, k]]

index[i, k] <- split[i]*(equals(t[i, k], pair[1]) + equals(t[i, k], pair[2]))

}

for(k in 2:na[i]){

theta[i, si[i, k]] ~ dnorm(md[i, si[i, k]], precd[i, si[i, k]])

**## True mean LOR split into direct and indirect via NMA**

md[i, si[i, k]] <- (d[si[i, k]] - d[bi[i]] + sw[i, k])*(1 - index[i, m[i, k]]) + direct*index[i, m[i, k]]

**## Adjusting for the correlated LORs for arms removed to split node**

j[i, k] <- k - (equals(1, split[i])*step(k - 3))

**## Precision of LOR distributions**

precd[i, si[i, k]] <- prec *2*(j[i,k] - 1)/j[i, k]

**## Adjustment, multi-arm RCTs**

w[i, k] <- (theta[i, si[i, k]] - d[si[i, k]] + d[bi[i]])*(1 - index[i, k])

**## Cumulative adjustment for multi-arm trials**

sw[i, k] <- sum(w[i, 1:(k-1)])/(j[i, k] - 1)

}

}

d[ref] <- 0

**## Prior consideration on logIMOR**

**[go to TABLE]** }

**## Basic parameters - prior distributions**

for(k in 1:(ref - 1)){

d[k] ~ dnorm(0, .0001)

}

for(k in (ref + 1):nt){

d[k] ~ dnorm(0, .0001)

}

direct ~ dnorm(0, .0001)

**## Predictive distribution for between-trial variance**

prec <- 1/tausq

tausq ~ dlnorm(mean.tausq, prec.tausq)

**## Calculate the difference between 'direct' and 'lor'**

diff <- direct - lor[pair[1], pair[2]] ## pair[2] vs pair[1]

**## Calculate p-value**

prob <- step(diff)

} **# END OF MODEL**

##############################################################################

# #

# **Node-splitting approach** with accommodation of multi-arm trials #

# (Dias et al., 2010 – PMID: 20213715) #

# **Selection model** with incorporation of log IMOR #

# (on average MAR and moderate prior variance) #

# (White et al., 2008 – PMID: 17703502) #

# (Spineli et al., 2013 – PMID: 23321265) #

# #

##############################################################################

**## INPUT PARAMETERS**

**# ns: number of trials in the network**

**# nt: number of interventions in the network**

**# y[k, i, 1:3]: an array consisting of the number of events, non-events, and MOD observed in arm k of trial i**

**# t[i, k]: intervention in arm k of trial i**

**# n[i, k]: number randomized in arm k of trial i**

**# na[i]: number of arms in trial i**

**# ref: selected reference intervention in the network**

**# split[i]: indicates the split (1 if node to split is present) and b (baseline position)**

**# m[i, k]: indexes to sweep non-baseline arms only**

**# bi[i]: vector of baseline interventions**

**# si[i, k]: matrix of non-baseline interventions**

**# pair: node to split**

**# mean.tausq: mean value of the selected log-normal empirical distribution for tausq**

**# prec.tausq: precision value of the selected log-normal empirical distribution for tausq**

model{ **# MODEL STARTS**

for(i in 1:ns){

w[i, 1] <- 0

j[i, 1] <- 0

theta[i, bi[i]] <- 0

mu[i] ~ dnorm(0, .0001)

for(k in 1:na[i]){

y[i, k, 1:3] ~ dmulti(prob[i, t[i, k], 1:3], n[i, k])

for(j in 1:3){

prob[i, t[i, k], j] <- 0.5*(3 - j)*(2 - j)*p[i, t[i, k]]*(1 - c1[i, t[i, k]]) +

(j - 1)*(3 - j)*(1 - p[i, t[i, k]])*(1 - c0[i, t[i, k]]) +

0.5*(1 - j)*(2 - j)*(p[i, t[i, k]]*c1[i, t[i, k]] + (1 - p[i, t[i, k]])*c0[i, t[i, k]])

}

logit(p[i, t[i, k]]) <- mu[i] + theta[i, t[i, k]]

index[i, k] <- split[i]*(equals(t[i, k], pair[1]) + equals(t[i, k], pair[2]))

}

for(k in 2:na[i]){

theta[i, si[i, k]] ~ dnorm(md[i, si[i, k]], precd[i, si[i, k]])

**## True mean LOR split into direct and indirect via NMA**

md[i, si[i, k]] <- (d[si[i, k]] - d[bi[i]] + sw[i, k])*(1 - index[i, m[i, k]]) + direct*index[i, m[i, k]]

**## Adjusting for the correlated LORs for arms removed to split node**

j[i, k] <- k - (equals(1, split[i])*step(k - 3))

**## Precision of LOR distributions**

precd[i, si[i, k]] <- prec*2*(j[i,k] - 1)/j[i, k]

**## Adjustment, multi-arm RCTs**

w[i, k] <- (theta[i, si[i, k]] - d[si[i, k]] + d[bi[i]])*(1 - index[i, k])

**## Cumulative adjustment for multi-arm trials**

sw[i, k] <- sum(w[i, 1:(k-1)])/(j[i, k] - 1)

}

}

d[ref] <- 0

**## Specify logIMORs and their structure** for(i in 1:ns){

for(i in 1:ns){

for(k in 1:na[i]){

delta[i, t[i, k]] <- **[go to TABLE]**

logit(alpha1[i, t[i, k]]) <- gamma[i, t[i, k]] + 0.5*delta[i, t[i, k]]

logit(alpha0[i, t[i, k]]) <- gamma[i, t[i, k]] - 0.5*delta[i, t[i, k]]

gamma[i, t[i, k]] ~ dnorm(0, gamma.prec)

}

}

**## Basic parameters - prior distributions**

for(k in 1:(ref - 1)){

d[k] ~ dnorm(0, .0001)

}

for(k in (ref + 1):nt){

d[k] ~ dnorm(0, .0001)

}

direct ~ dnorm(0, .0001)

gamma.prec <- 0.001

**## Predictive distribution for between-trial variance**

prec <- 1/tausq

tausq ~ dlnorm(mean.tausq, prec.tausq)

**## Calculate the difference between 'direct' and 'lor'**

diff <- direct - lor[pair[1], pair[2]] ## pair[2] vs pair[1]

**## Calculate p-value**

prob <- step(diff)

} **# END OF MODEL**

**TABLE**

**Structure of** $\boldsymbol{\delta}_{\boldsymbol{ik}}$**s and their prior distribution assigned under on average MAR with moderate prior variance**

| **Structure** | **Structural assumption** | **Define** $\boldsymbol{\delta}_{\boldsymbol{ik}}$ | **Define relevant prior distribution(s)** |
| --- | --- | --- | --- |
| Identical | Common-within-network | log(IMOR[i, k]) <- delta | delta ~ dnorm(0.0001, 1) |
|  | Trial-specific | log(IMOR[i, k]) <- delta[i] | for(i in 1:ns){  delta[i] ~ dnorm(0.0001, 1)  } |
|  | Intervention-specific | log(IMOR[i, k]) <- delta[t[i, k]] | for(t in 1:nt){  delta[t] ~ dnorm(0.0001, 1)  } |
| Hierarchical | Common-within-network | log(IMOR[i, k]) <- delta[i, k] | for(i in 1:ns){  for(k in 1:na[i]){  delta[i, k] ~ dnorm(D, prec.d)  }  }  D ~ dnorm(0.0001, 1)  prec.d <- pow(sd.d, -2)  sd.d ~ dunif(0, 1)  tausq.d <- pow(sd.d, 2) |
|  | Trial-specific | log(IMOR[i, k]) <- delta[i, k] | for(i in 1:ns){  D[i] ~ dnorm(0.0001, 1)  prec.d[i] <- pow(sd.d[i], -2)  sd.d[i] ~ dunif(0, 1)  tausq.d[i] <- pow(sd.d[i], 2)  for(k in 1:na[i]){  delta[i, k] ~ dnorm(D[i], prec.d[i])  }  } |
|  | Intervention-specific | log(IMOR[i, k]) <- delta[i, k] | for(i in 1:ns){  for(k in 1:na[i]){  delta[i, k] ~ dnorm(D[t[i, k]], prec.d[t[i, k]]))  }  }  for(t in 1:nt){  D[t] ~ dnorm(0.0001, 1)  prec.d[t] <- pow(sd.d[t], -2)  sd.d[t] ~ dunif(0, 1)  tausq.d[t] <- pow(sd.d[t], 2)  } |
