# Supplementary material for: An empirical comparison of Bayesian modelling strategies for missing binary outcome data in network meta-analysis
Source: BMC Med Res Methodol. 2019 Apr 24;19:86. doi: 10.1186/s12874-019-0731-y (PMC6480793; doi:10.1186/s12874-019-0731-y)
Supplement: Supplementary file 5 — Supplementary Figures. (DOCX 5671 kb) [file 12874_2019_731_MOESM5_ESM.docx]

**Additional file 5. Supplementary Figures**

| **Agreement between on average missing at random and extreme scenarios** |
| --- |


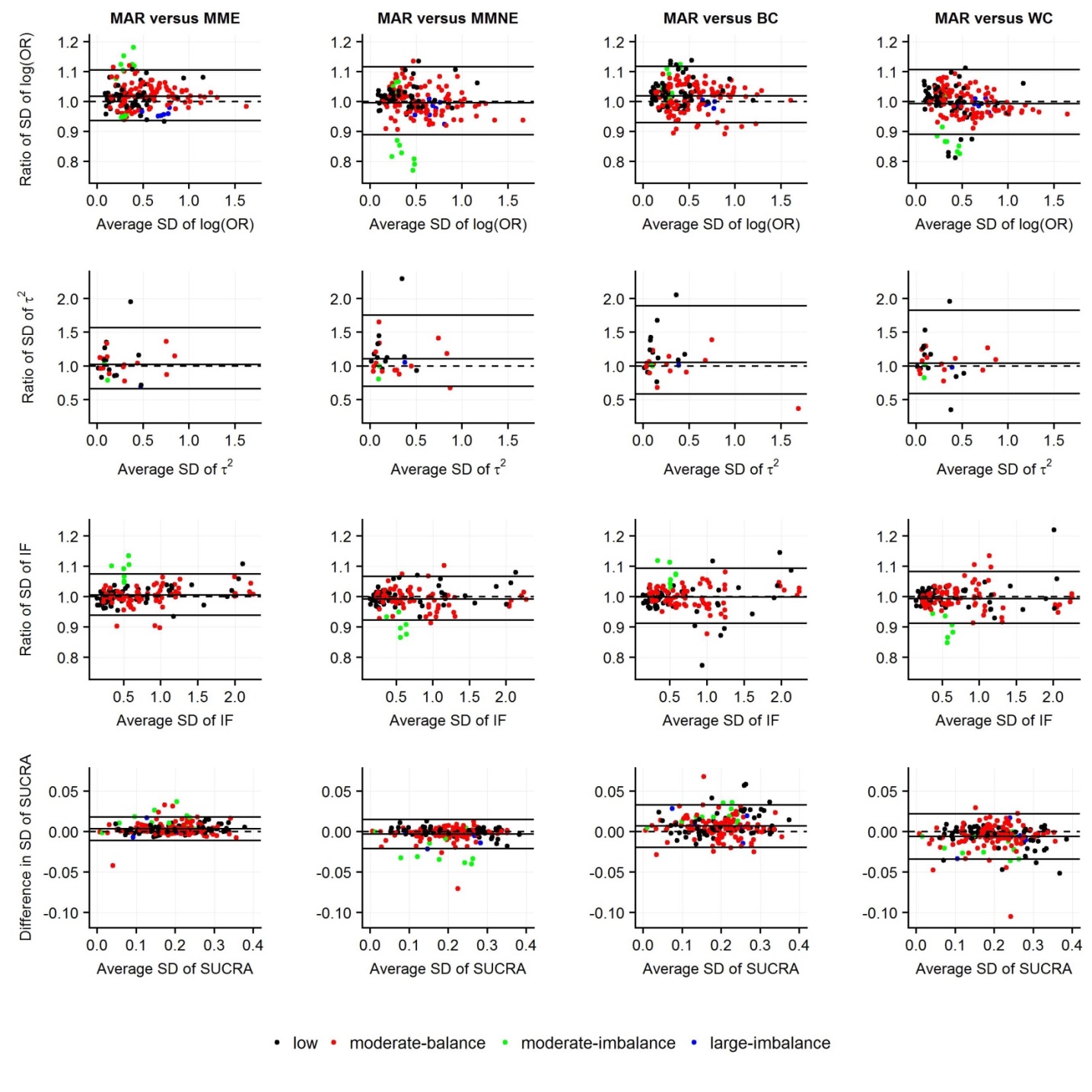


**Figure S1(a).** A series of Bland-Altman plots that illustrate *level of agreement between on average missing at random and four extreme scenarios* in terms of posterior standard deviation of log odds ratio for basic parameters (first row), posterior standard deviation of common between-trial variance (second row), posterior standard deviation of inconsistency factors (third row) and posterior standard deviation of SUCRA values (fourth row). Use of identical, intervention-specific, normal prior distribution on log IMORs with moderate prior variance. Different colors indicate extent and balance of MOD across 29 networks (17 networks with at least one closed loop). BC, best-case scenario; IF, inconsistency factor; MAR, (on average) missing at random; MME, more missing cases are events in all interventions; MMNE, more missing cases are non-events in all interventions; OR, odds ratio; SD, standard deviation; SUCRA, surface under cumulative ranking; WC, worst-case scenario.


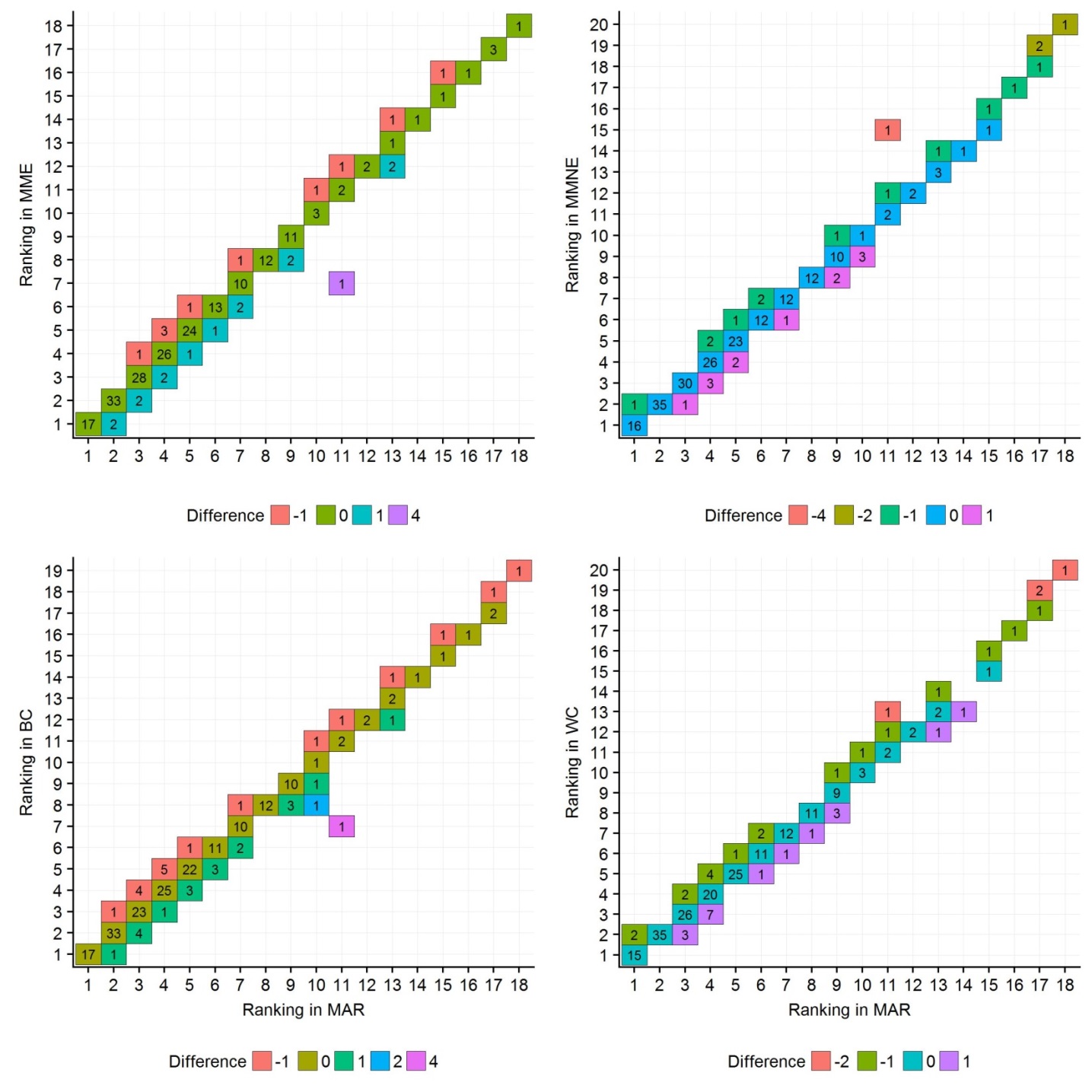


**Figure S1(b).** A series of heat-maps that illustrate *level of agreement between on average missing at random and four extreme scenarios* in terms of posterior median of intervention ranking. Use of identical, intervention-specific, normal prior distribution on log IMORs with moderate prior variance. The colored boxes indicate the number of rankings won or lost when an (on average) extreme scenario is considered instead of on average MAR. The numbers within the boxes indicate frequency of interventions that achieved a specific pair of rankings. Smaller rankings reflect a better position in the intervention hierarchy. BC, best-case scenario; MAR, (on average) missing at random; MME, more missing cases are events in all interventions; MMNE, more missing cases are non-events in all interventions; WC, worst-case scenario.

| **Agreement between accounting and discounting uncertainty due to MOD** |
| --- |


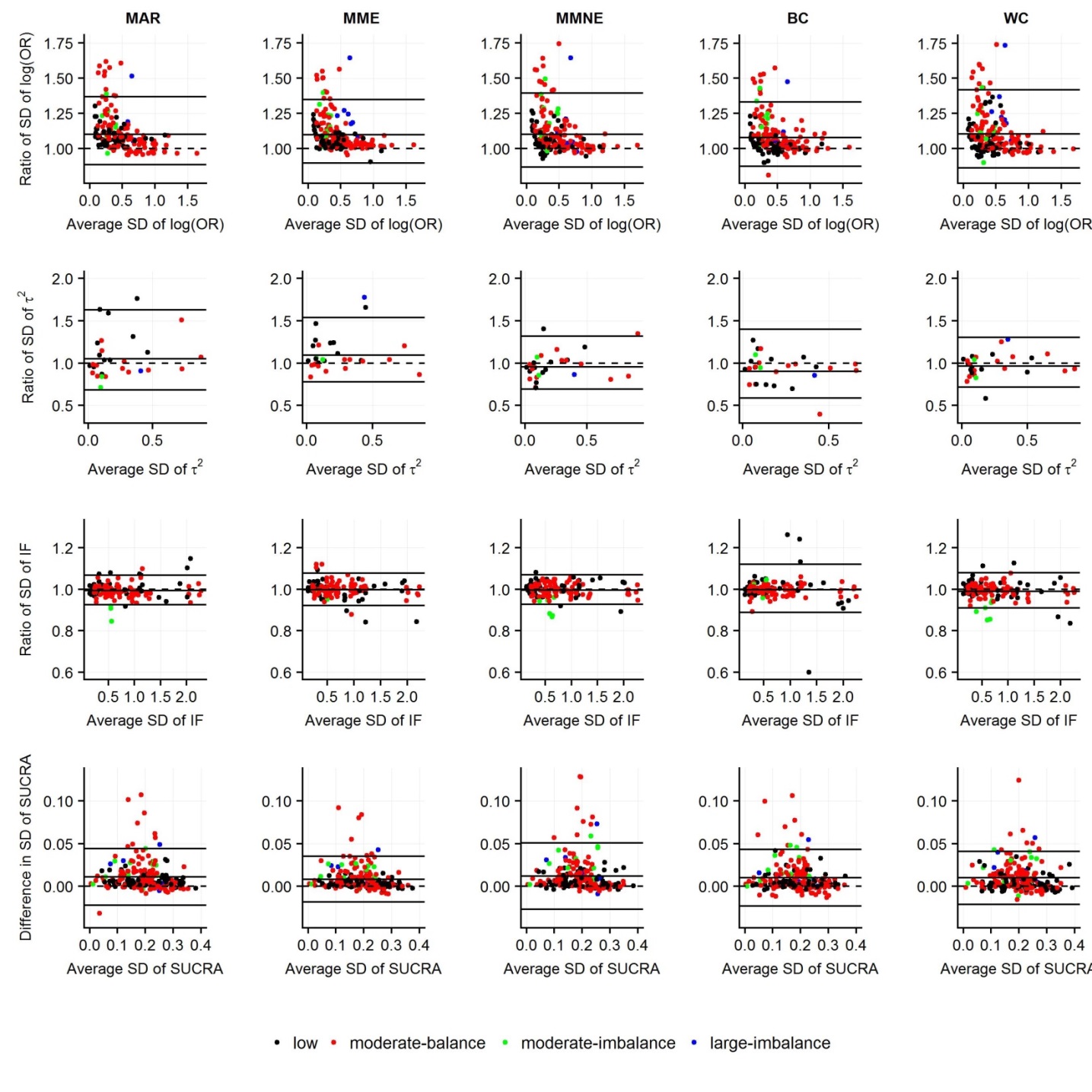


**Figure S2(a).** A series of Bland-Altman plots that illustrate *level of agreement between* *accountability and ignorance of uncertainty* *due to missingness* under missing at random and four extreme scenarios in terms of the posterior standard deviation of log odds ratio for basic parameters (first row), posterior standard deviation of common between-trial variance (second row), posterior standard deviation of inconsistency factors (third row) and posterior standard deviation of SUCRA values (fourth row). Use of identical, intervention-specific normal prior distribution on log IMORs with moderate and zero prior variance to reflect accountability and ignorance of uncertainty due to missingness, respectively. Different colors indicate extent and balance of MOD across 29 networks (17 networks with at least one closed loop). BC, best-case scenario; IF, inconsistency factor; MAR, missing at random; MME, more missing cases are events in all interventions; MMNE, more missing cases are non-events in all interventions; OR, odds ratio; SD, standard deviation; SUCRA, surface under cumulative ranking; WC, worst-case scenario.


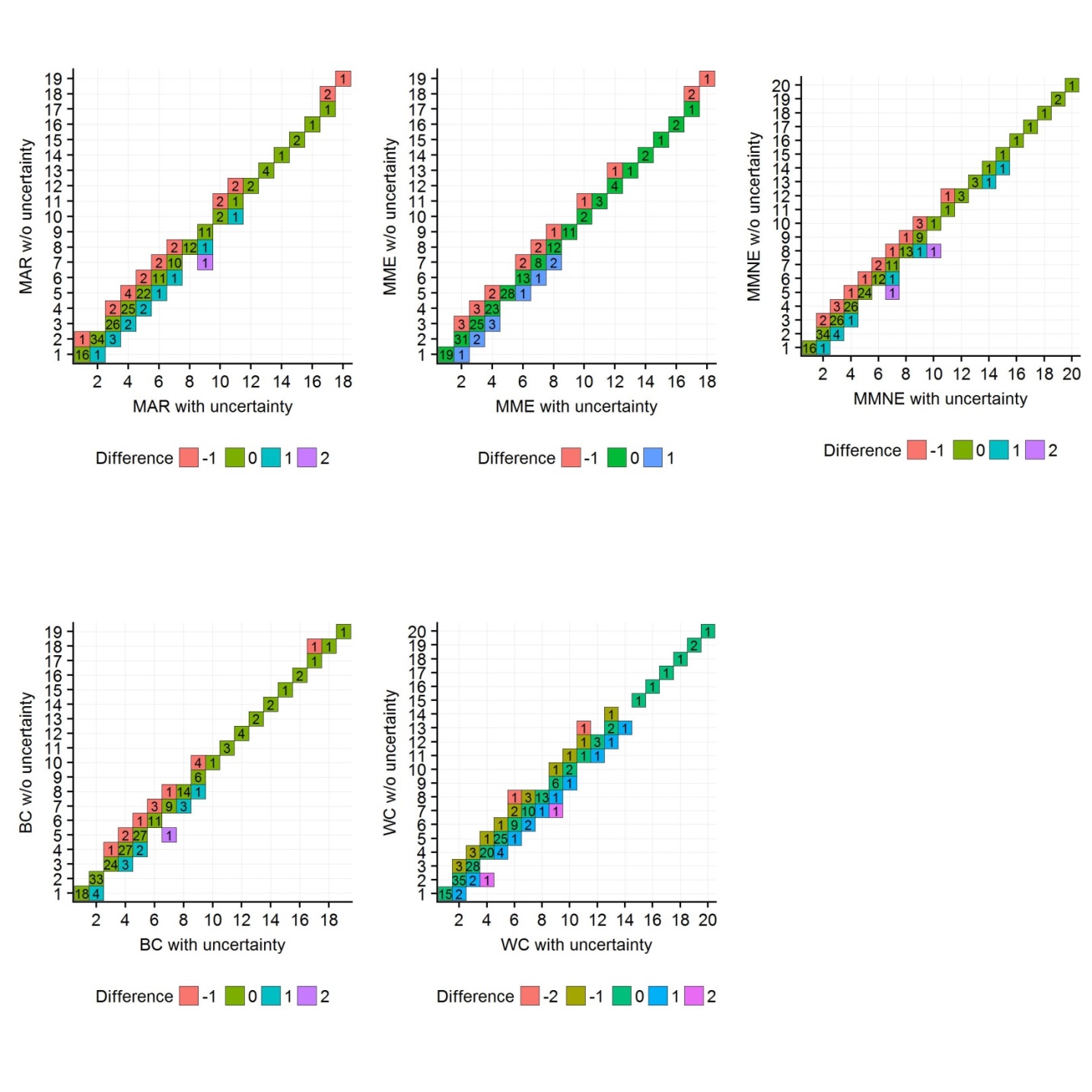


**Figure S2(b).** A series of heat-maps that illustrate *level of agreement between* *accountability and ignorance of uncertainty* *due to missingness* under missing at random and four extreme scenarios in terms of posterior median of intervention ranking. Use of identical, intervention-specific, normal prior distribution on log IMORs with moderate and zero prior variance to reflect accountability and ignorance of uncertainty due to missingness, respectively. The colored boxes indicate the number of rankings won or lost when uncertainty due to missingness is discounted rather than accounted. The numbers within the boxes indicate frequency of interventions that achieved a specific pair of rankings. Smaller rankings reflect a better position in the intervention hierarchy. BC, best-case scenario; MAR, missing at random; MME, more missing cases are events in all interventions; MMNE, more missing cases are non-events in all interventions; WC, worst-case scenario; w/o, without.

| **Agreement between identical and hierarchical prior structure for log IMOR** |
| --- |


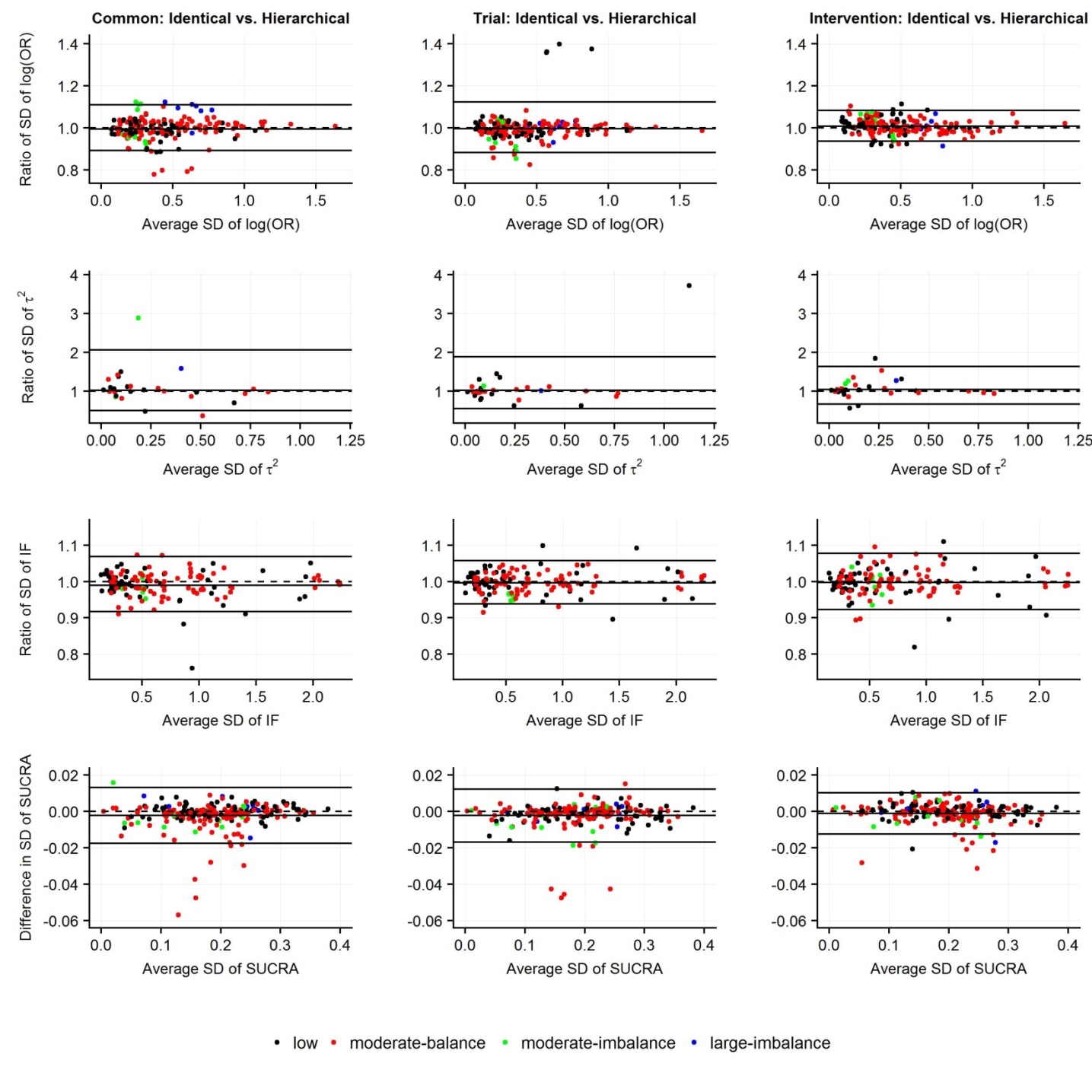


**Figure S3(a).** A series of Bland-Altman plots that illustrate *level of agreement between identical and hierarchical structure of log IMORs* in terms of posterior standard deviation of log odds ratio for basic parameters (first row), posterior standard deviation of common between-trial variance (second row), posterior standard deviation of inconsistency factors (third row) and posterior standard deviation of SUCRA values (fourth row) with respect to common-within-network, trial-specific and intervention-specific, normal prior distribution on log IMORs under on average missing at random with moderate prior variance. Different colors indicate extent and balance of MOD across 29 networks (17 networks with at least one closed loop). Common, common-within-network; IF, inconsistency factor; Intervention, intervention-specific; OR, odds ratio; SD, standard deviation; SUCRA, surface under cumulative ranking; Trial, trial-specific.


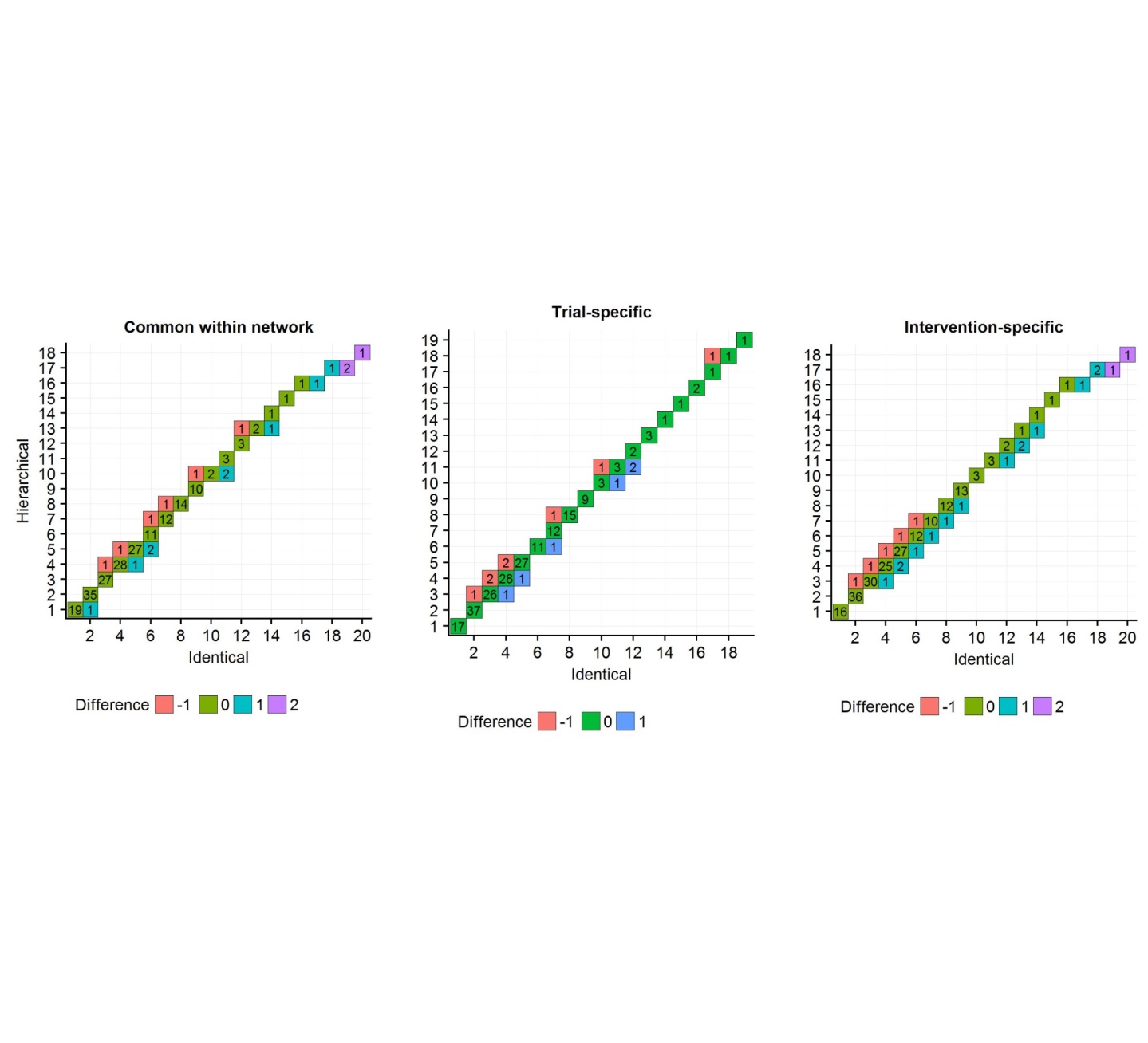


**Figure S3(b).** A series of heat-maps that illustrate *level of agreement between identical and hierarchical structure of log IMORs* in terms of posterior median of intervention ranking with respect to common-within-network, trial-specific and intervention-specific, normal prior distribution on log IMORs under on average missing at random with moderate prior variance. The colored boxes indicate the number of rankings won or lost when hierarchical than identical log IMORs are assumed. The numbers within the boxes indicate frequency of interventions that achieved a specific pair of rankings. Smaller rankings reflect a better position in the intervention hierarchy.


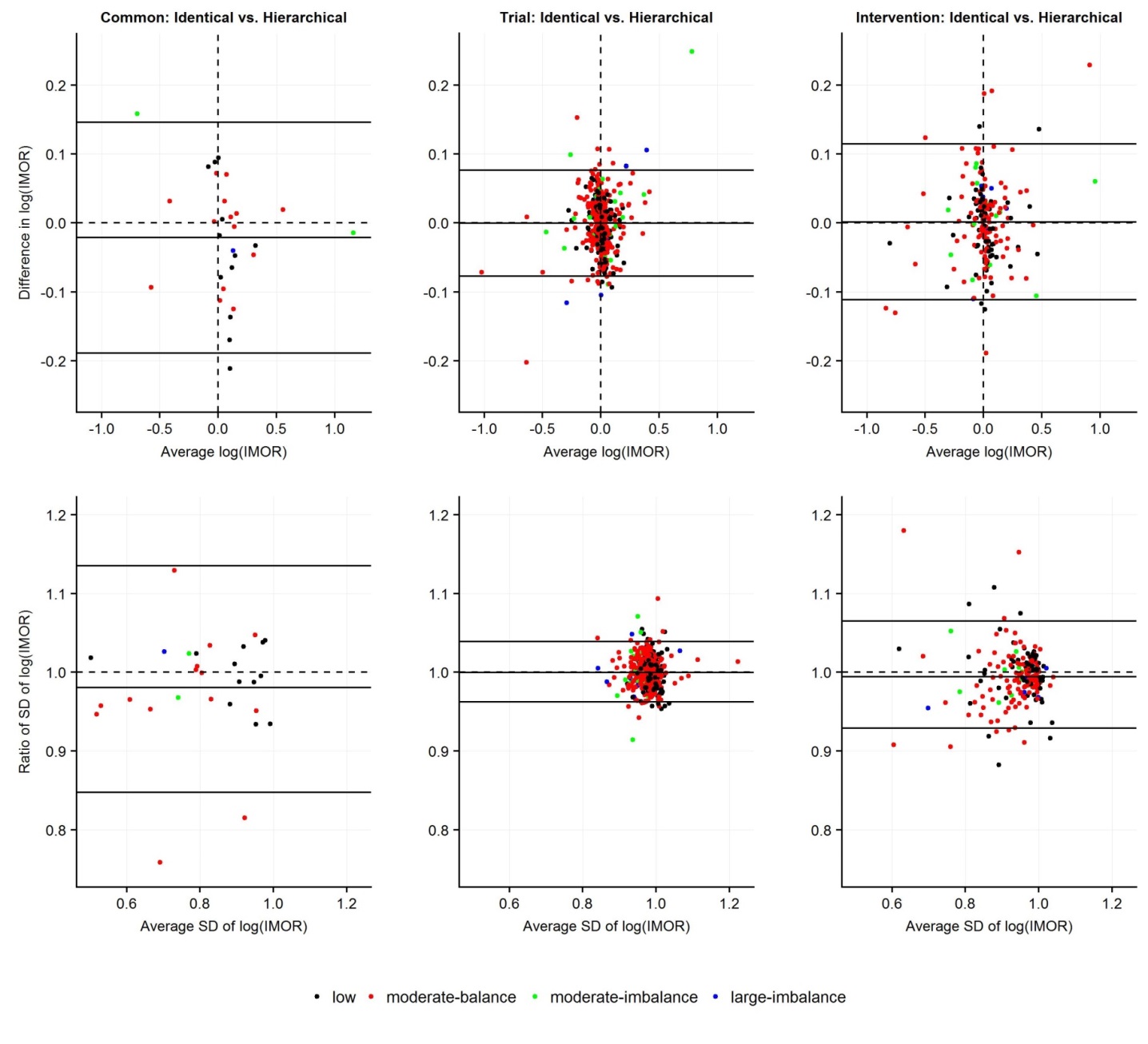


**Figure S3(c).** A series of Bland-Altman plots that illustrate *level of agreement between identical and hierarchical structure of log IMORs* in terms of posterior mean of log IMORs (first row) and posterior standard deviation of log IMORs (second row) with respect to common-within-network, trial-specific and intervention-specific, normal prior distribution on log IMORs under on average missing at random with moderate prior variance. Different colors indicate extent and balance of MOD across 29 networks (17 networks with at least one closed loop). Common, common-within-network; IMOR, informative missingness odds ratio; Intervention, intervention-specific; SD, standard deviation; Trial, trial-specific.

| **Agreement among different normal prior distribution structures for log IMOR** |
| --- |

**Identical structure**


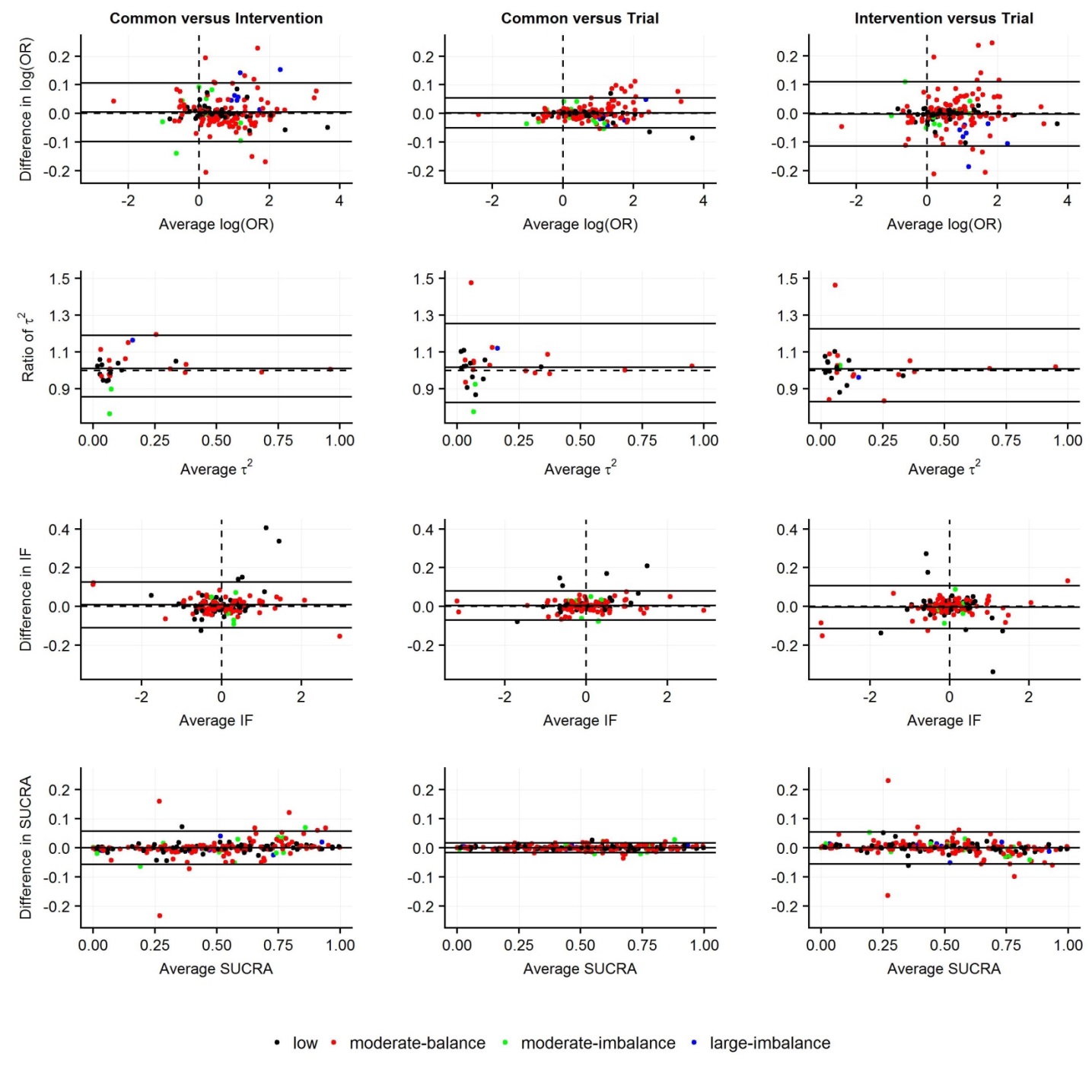


**Figure S4(a).** A series of Bland-Altman plots that illustrate *level of agreement among different structures of normal prior distribution on identical log IMORs* under on average missing at random with moderate prior variance in terms of posterior mean of log odds ratio for basic parameters (first row), posterior median of common between-trial variance (second row), posterior mean of inconsistency factors (third row) and posterior mean of SUCRA values (fourth row). Different colors indicate extent and balance of MOD across 29 networks (17 networks with at least one closed loop). Common, common-within-network; IF, inconsistency factor; Intervention, intervention-specific; OR, odds ratio; SUCRA, surface under cumulative ranking; Trial, trial-specific.


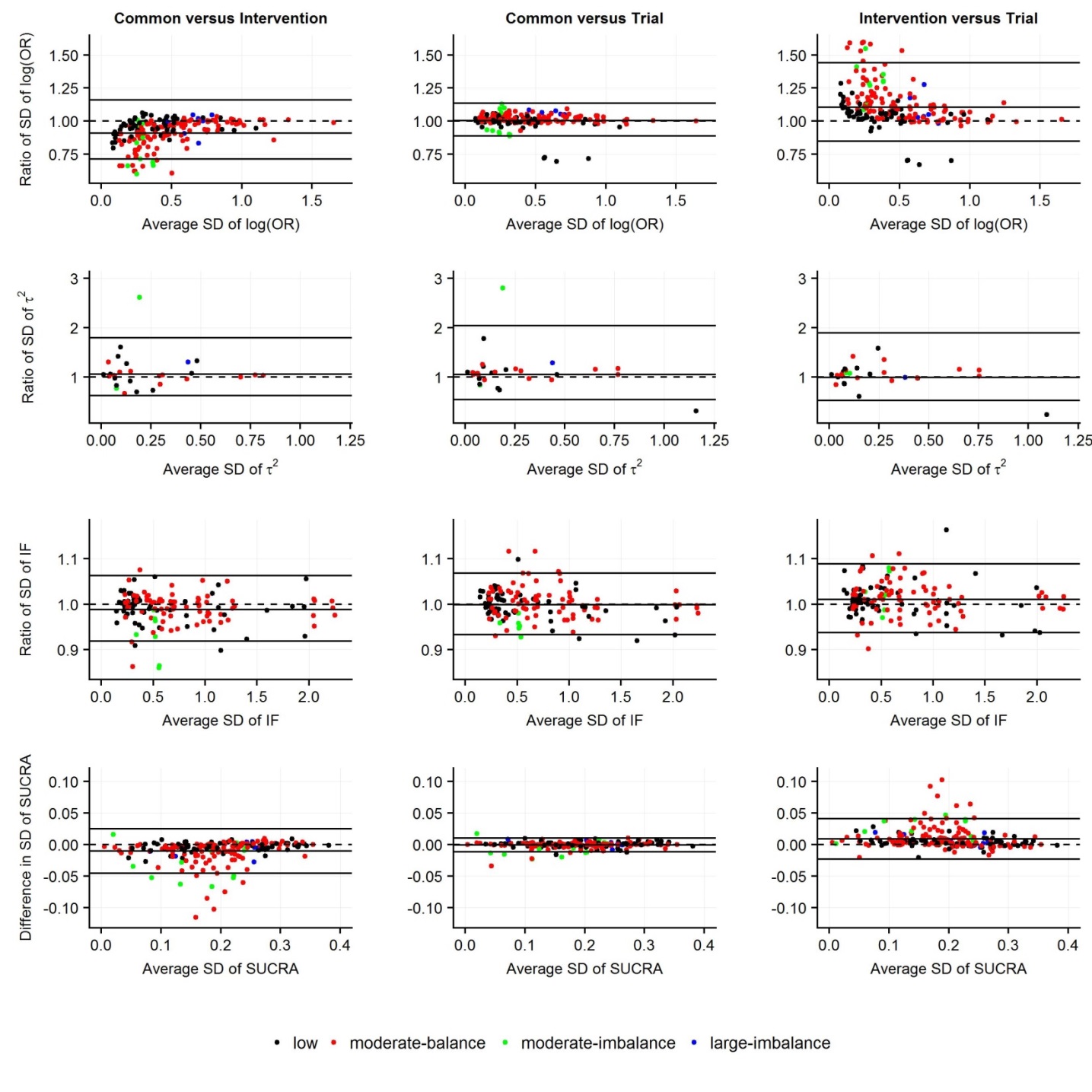


**Figure S4(b).** A series of Bland-Altman plots that illustrate *level of agreement among different structures of normal prior distribution on identical log IMORs* under on average missing at random with moderate prior variance in terms of posterior standard deviation of log odds ratio for basic parameters (first row), posterior standard deviation of common between-trial variance (second row), posterior standard deviation of inconsistency factors (third row) and posterior standard deviation of SUCRA values (fourth row). Different colors indicate extent and balance of MOD across 29 networks (17 networks with at least one closed loop). Common, common-within-network; IF, inconsistency factor; Intervention, intervention-specific; OR, odds ratio; SD, standard deviation; SUCRA, surface under cumulative ranking; Trial, trial-specific.


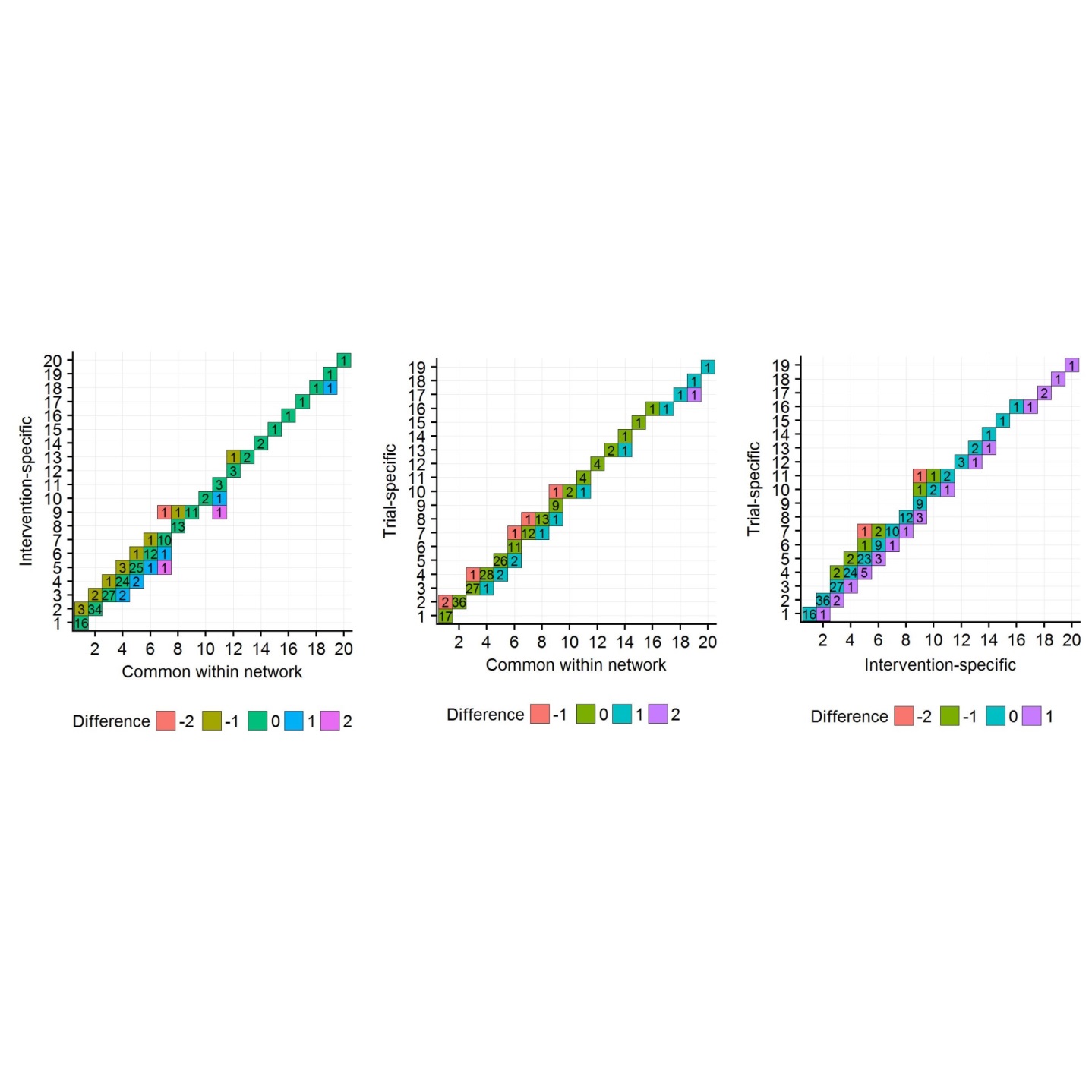


**Figure S4(c).** A series of heat-maps that illustrate *level of agreement among different structures of normal prior distribution on identical log IMORs* under on average missing at random with moderate prior variance in terms of posterior median of intervention ranking. The colored boxes indicate the number of rankings won or lost when one prior structure (that in y axis) than another (that in x axis) is assumed. The numbers within the boxes indicate frequency of interventions that achieved a specific pair of rankings. Smaller rankings reflect a better position in the intervention hierarchy.

| **Agreement among different normal prior distribution structures for log IMOR** |
| --- |

**Hierarchical structure**


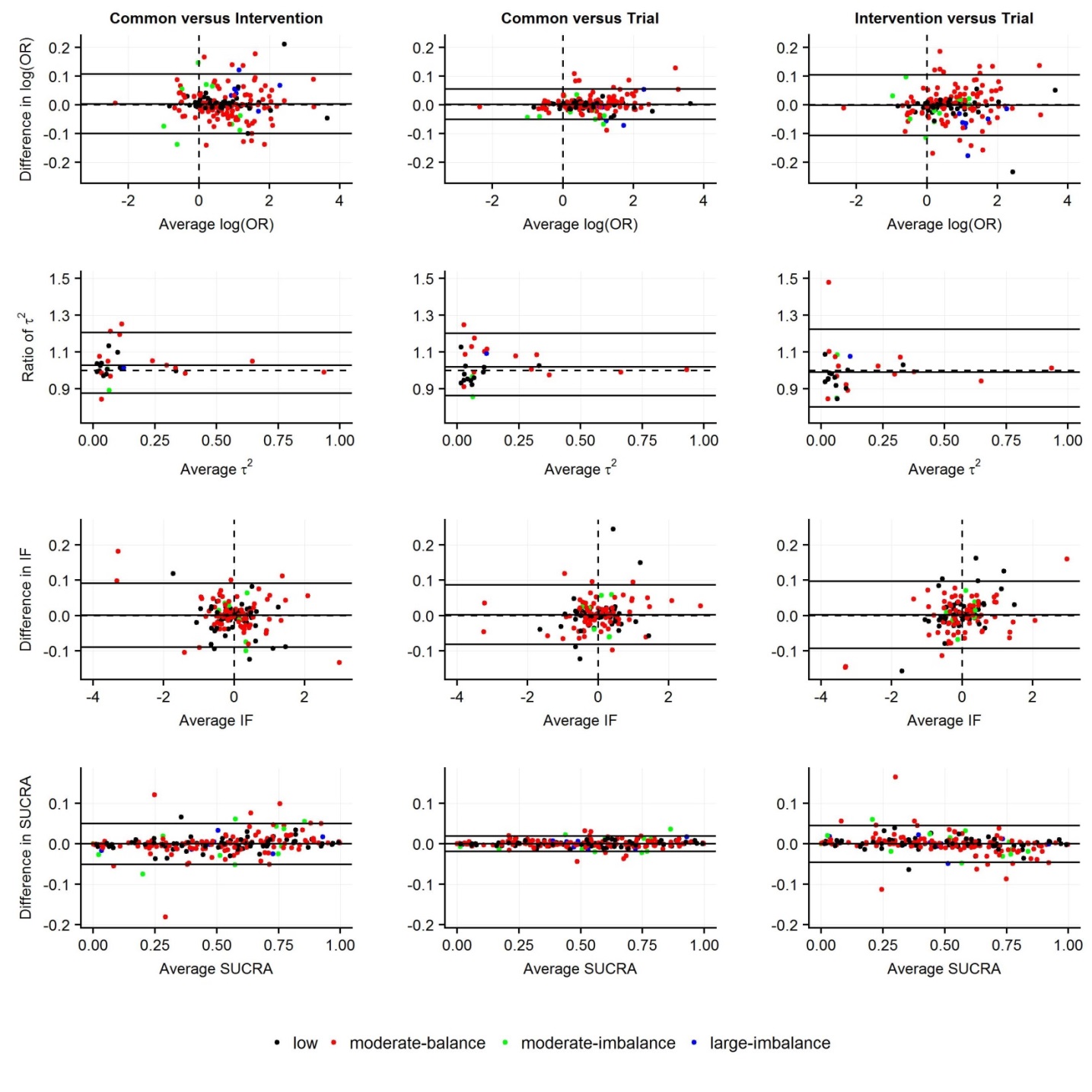


**Figure S5(a).** A series of Bland-Altman plots that illustrate *level of agreement among different structures of normal prior distribution on hierarchical log IMORs* under on average missing at random with moderate prior variance in terms of posterior mean of log odds ratio for basic parameters (first row), posterior median of common between-trial variance (second row), posterior mean of inconsistency factors (third row) and posterior mean of SUCRA values (fourth row). Different colors indicate extent and balance of MOD across 29 networks (17 networks with at least one closed loop). Common, common-within-network; IF, inconsistency factor; Intervention, intervention-specific; OR, odds ratio; SUCRA, surface under cumulative ranking; Trial, trial-specific.


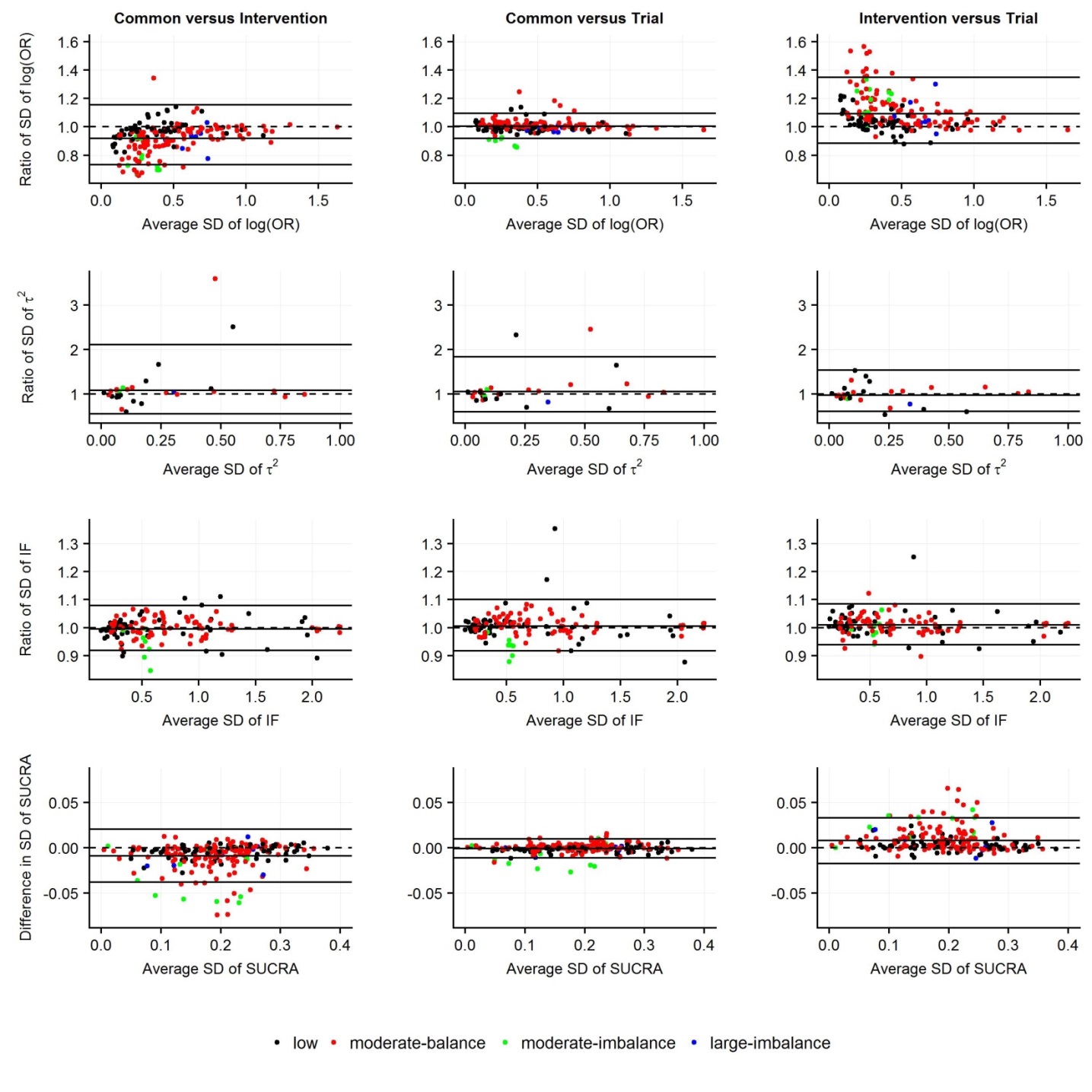


**Figure S5(b).** A series of Bland-Altman plots that illustrate *level of agreement among different structures of normal prior distribution on hierarchical log IMORs* under on average missing at random with moderate prior variance in terms of posterior standard deviation for log odds ratio of basic parameters (first row), posterior standard deviation of common between-trial variance (second row), posterior standard deviation of inconsistency factors (third row) and posterior standard deviation of SUCRA values (fourth row). Different colors indicate extent and balance of MOD across 29 networks (17 networks with at least one closed loop). Common, common-within-network; IF, inconsistency factor; Intervention, intervention-specific; OR, odds ratio; SD, standard deviation; SUCRA, surface under cumulative ranking; Trial, trial-specific


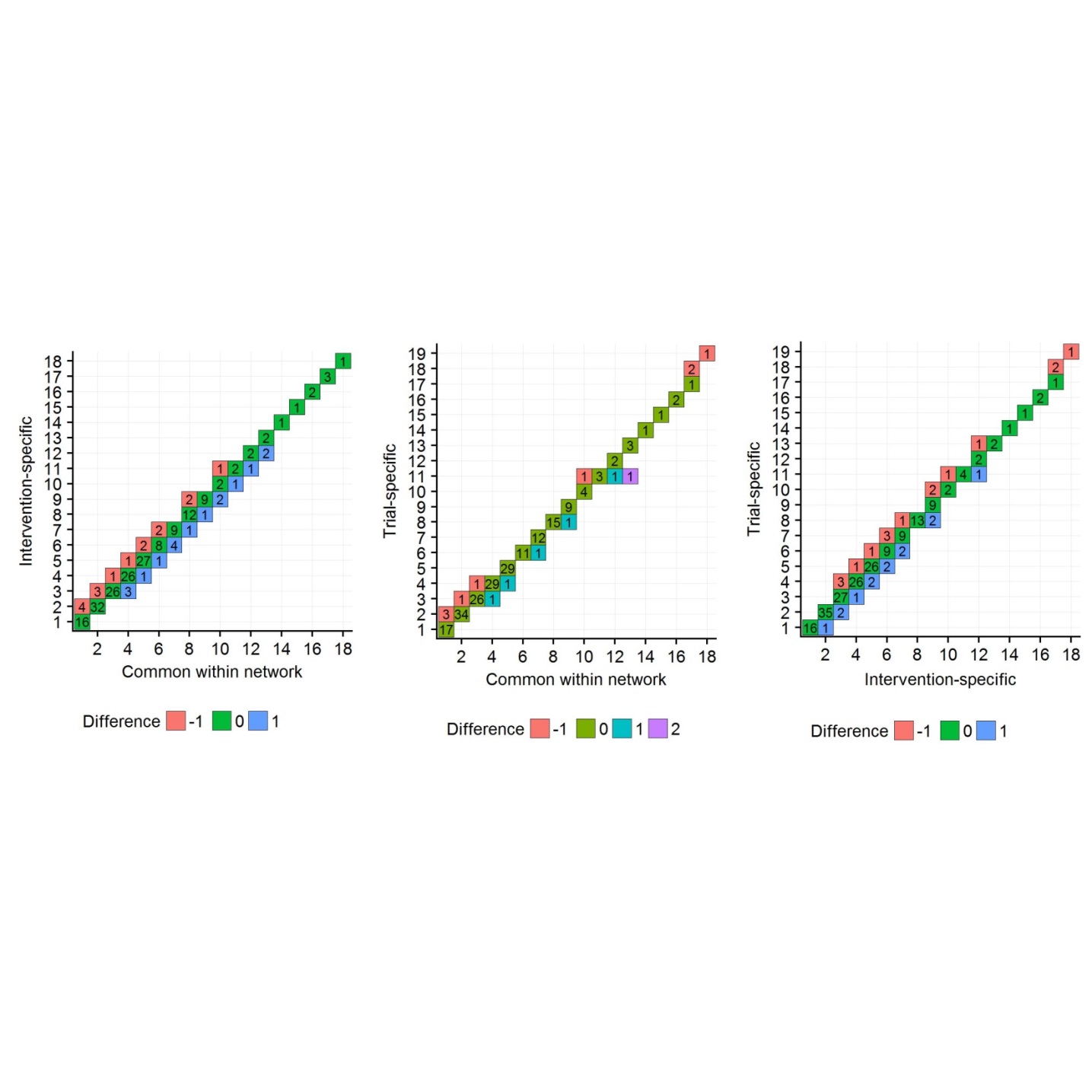


**Figure S5(c).** A series of heat-maps that illustrate *level of agreement among different structures of normal prior distribution on hierarchical log IMORs* under on average missing at random with moderate prior variance in terms of posterior median of intervention ranking. The colored boxes indicate the number of rankings won or lost when one prior structure (that in y axis) than another (that in x axis) is assumed. The numbers within the boxes indicate frequency of interventions that achieved a specific pair of rankings. Smaller rankings reflect a better position in the intervention hierarchy.

| **Agreement between pattern-mixture model and selection model** |
| --- |


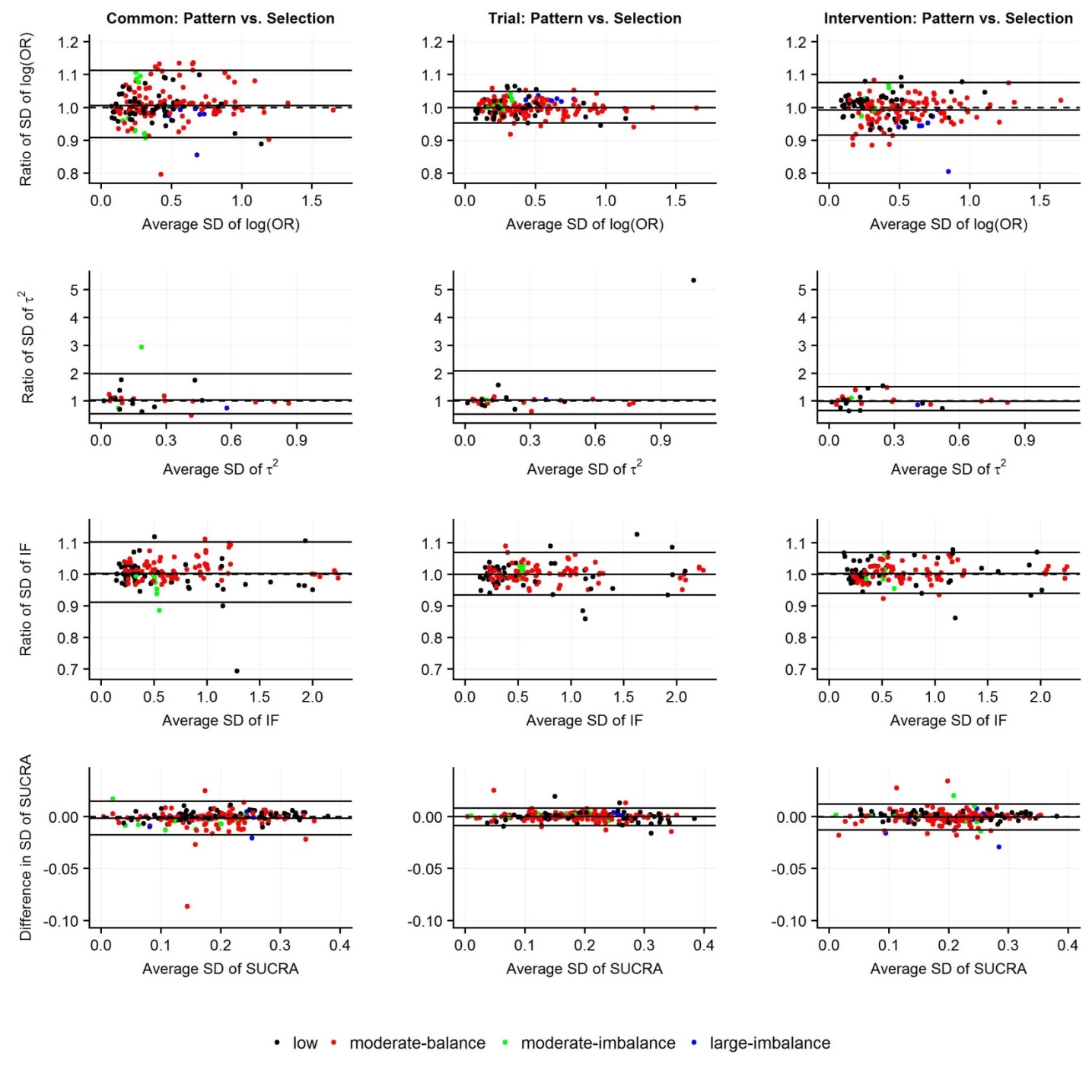


**Figure S6(a).** A series of Bland-Altman plots that illustrate *level of agreement between pattern-mixture model and selection model* in terms of posterior standard deviation of log odds ratio for basic parameters (first row), posterior standard deviation of common between-trial variance (second row), posterior standard deviation of inconsistency factors (third row) and posterior standard deviation of SUCRA values (fourth row) with respect to common-within-network, trial-specific and intervention-specific, normal prior distribution on identical log IMORs under on average missing at random with moderate prior variance. Different colors indicate extent and balance of MOD across 29 networks (17 networks with at least one closed loop). Common, common-within-network; IF, inconsistency factor; Intervention, intervention-specific; OR, odds ratio; SD, standard deviation; SUCRA, surface under cumulative ranking; Trial, trial-specific.


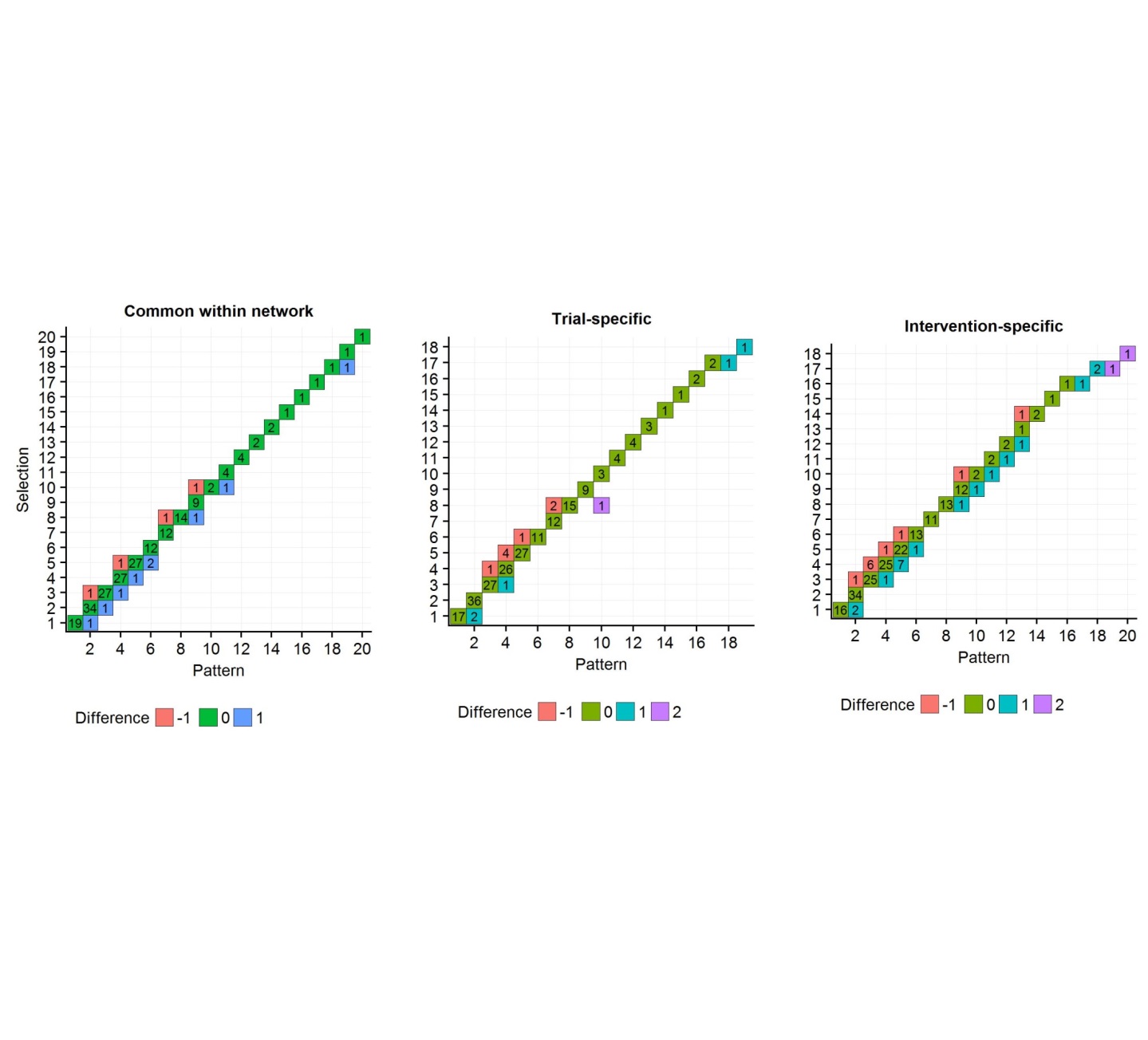


**Figure S6(b).** A series of heat-maps that illustrate *level of agreement between pattern-mixture model and selection model* in terms of posterior median of intervention ranking with respect to common-within-network, trial-specific and intervention-specific, normal prior distribution on identical log IMORs under on average missing at random with moderate prior variance. The colored boxes indicate the number of rankings won or lost when selection model than pattern-mixture model is assumed. The numbers within the boxes indicate frequency of interventions that achieved a specific pair of rankings. Smaller rankings reflect a better position in the intervention hierarchy.


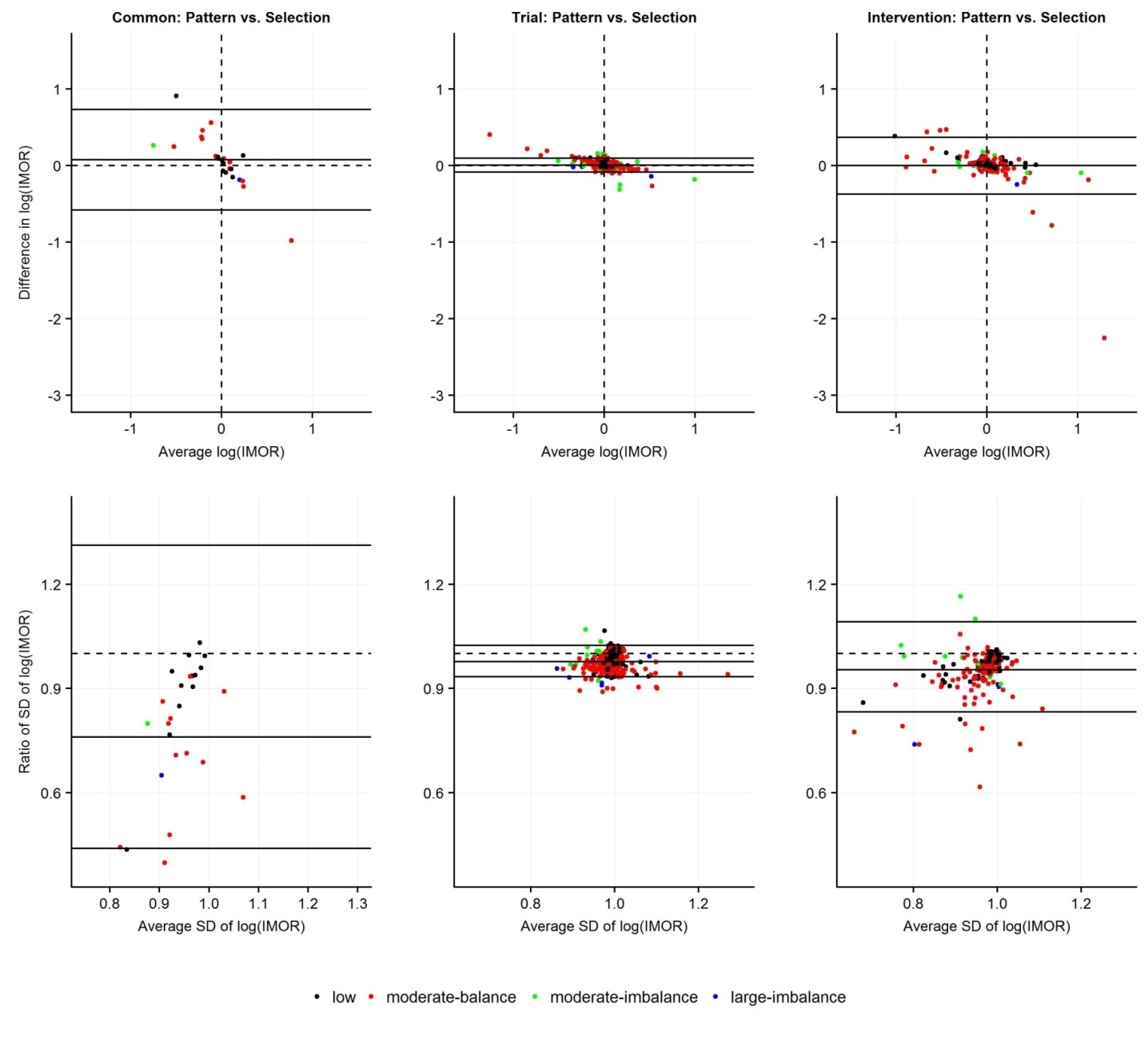


**Figure S6(c).** A series of Bland-Altman plots that illustrate *level of agreement between pattern-mixture model and selection model* in terms of posterior mean of log IMORs (first) and posterior standard deviation of log IMORs (second row) with respect to common-within-network, trial-specific and intervention-specific, normal prior distribution on identical log IMORs under on average missing at random with moderate prior variance. Different colors indicate extent and balance of MOD across 29 networks (17 networks with at least one closed loop). Common, common-within-network; IMOR, informative missingness odds ratio; Intervention, intervention-specific; SD, standard deviation; Trial, trial-specific.

| **Agreement between moderate and other prior variances for log IMOR** |
| --- |


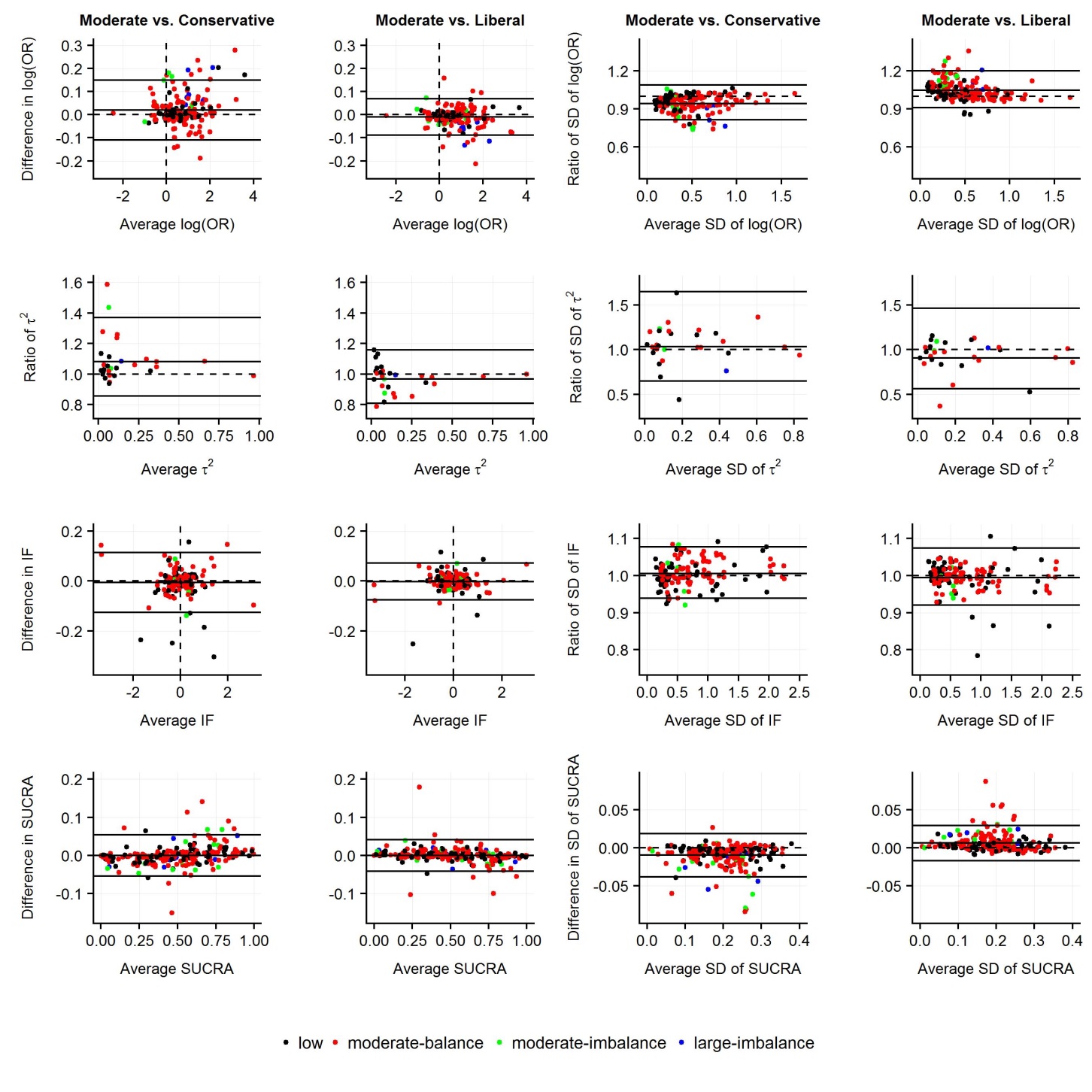


**Figure S7(a).** A series of Bland-Altman plots that illustrate *level of agreement between moderate and alternative prior variances for log IMOR* in terms of posterior mean of log odds ratio for basic parameters (first row – left panel), posterior median of common between-trial variance (second row – left panel), posterior mean of inconsistency factors (third row – left panel) and posterior mean of SUCRA values (fourth row – left panel) *as well as* posterior standard deviation of log odds ratio for basic parameters (first row – right panel), posterior standard deviation of common between-trial variance (second row – right panel), posterior standard deviation of inconsistency factors (third row – right panel) and posterior standard deviation of SUCRA values (fourth row – right panel). Use of identical, intervention-specific, normal prior distribution on log IMORs under on average missing at random. Different colors indicate extent and balance of MOD across 29 networks (17 networks with at least one closed loop). IF, inconsistency factor; OR, odds ratio; SD, standard deviation; SUCRA, surface under cumulative ranking.


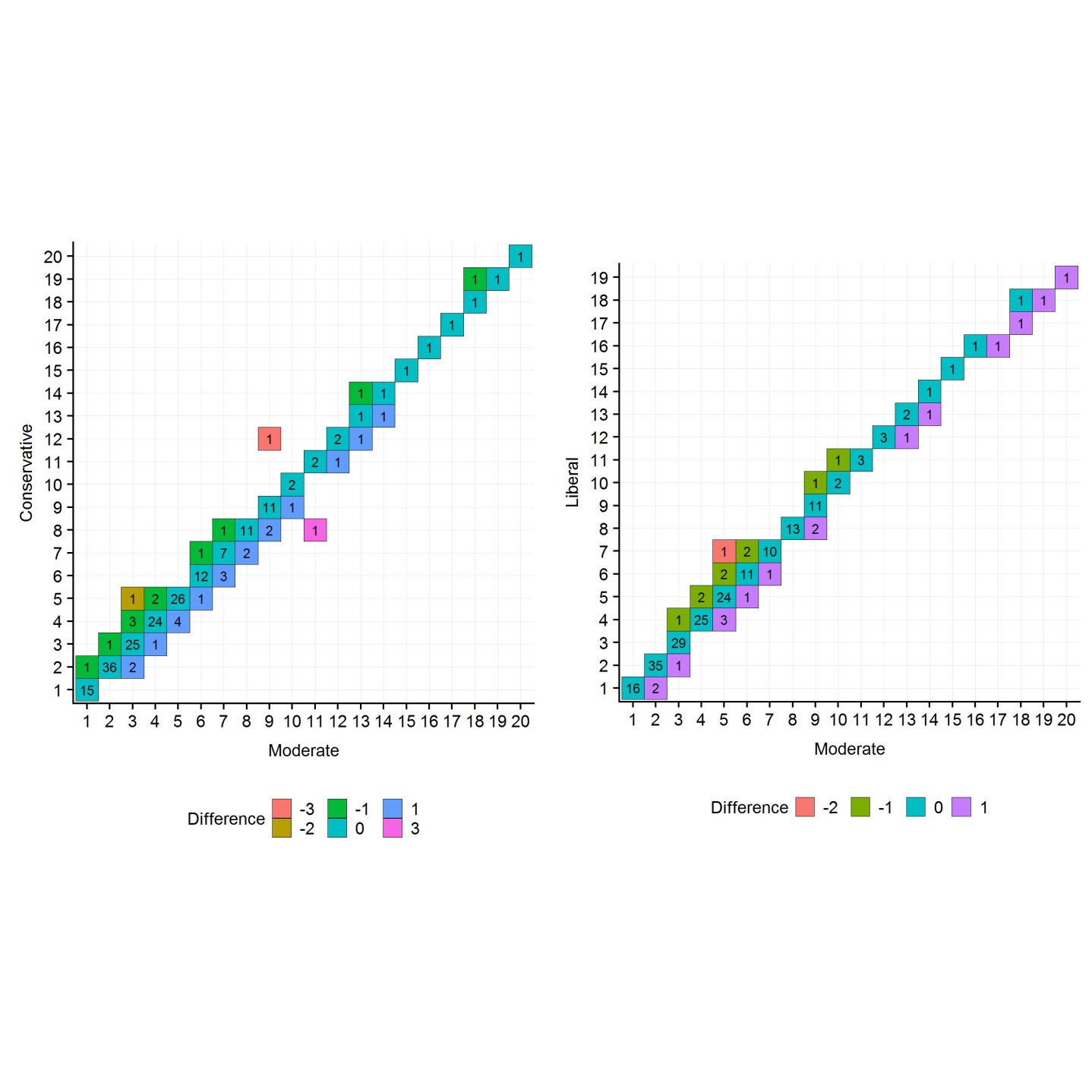


**Figure S7(b).** A series of heat-maps that illustrate *level of agreement between moderate and alternative prior variances for log IMOR* in terms of posterior median of intervention ranking. Use of identical, intervention-specific, normal prior distribution on log IMORs under on average missing at random. The colored boxes indicate the number of rankings won or lost when alternative than moderate prior variance for log IMOR is assumed. The numbers within the boxes indicate frequency of interventions that achieved a specific pair of rankings. Smaller rankings reflect a better position in the intervention hierarchy.


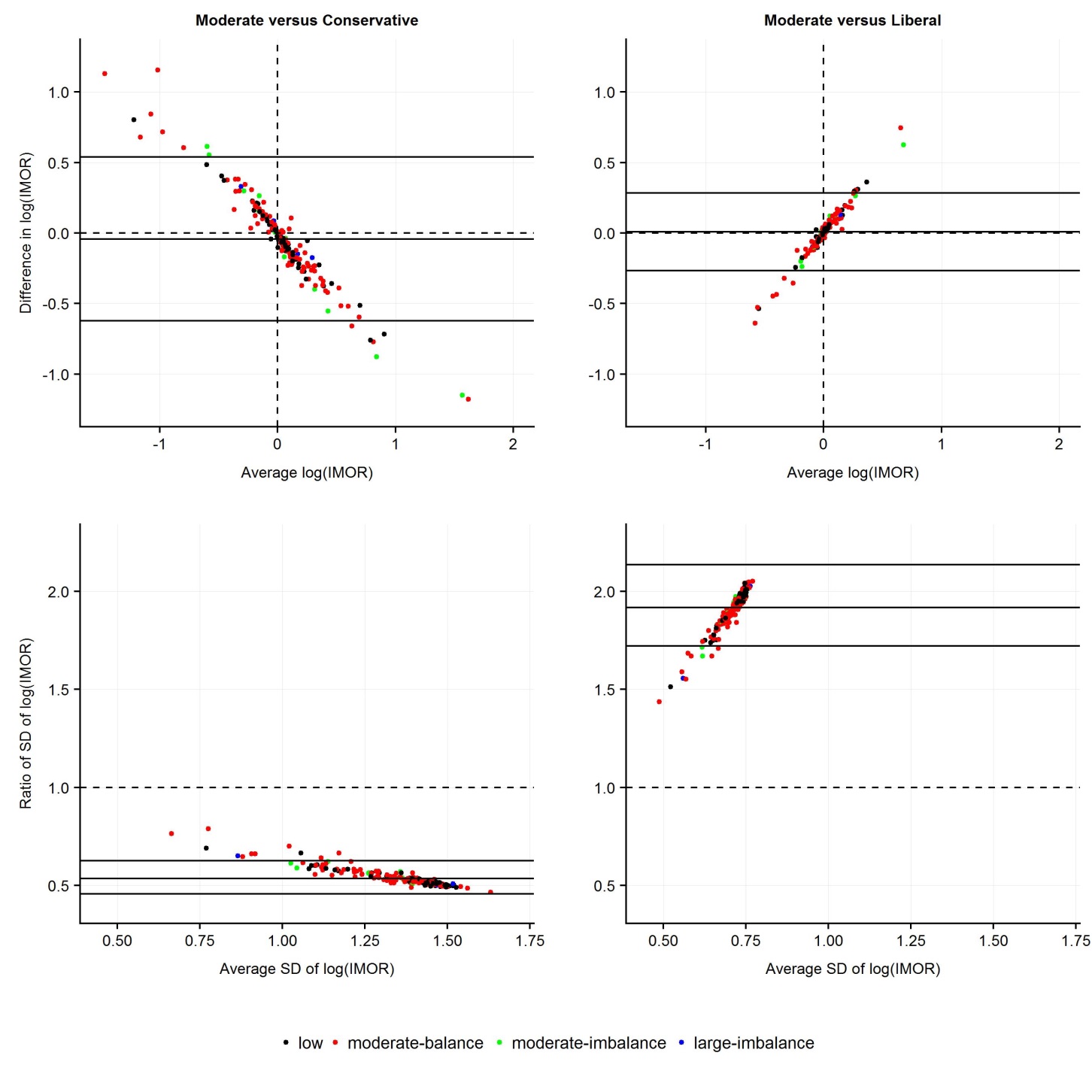


**Figure S7(c).** A series of Bland-Altman plots that illustrate *level of agreement between moderate and alternative prior variances for log IMOR* in terms of posterior mean of log IMORs (first row) and posterior standard deviation of log IMORs (second row). Use of identical, intervention-specific, normal prior distribution on log IMORs under on average missing at random. Different colors indicate extent and balance of MOD across 29 networks (17 networks with at least one closed loop). IMOR, informative missingness odds ratio; SD, standard deviation.

**
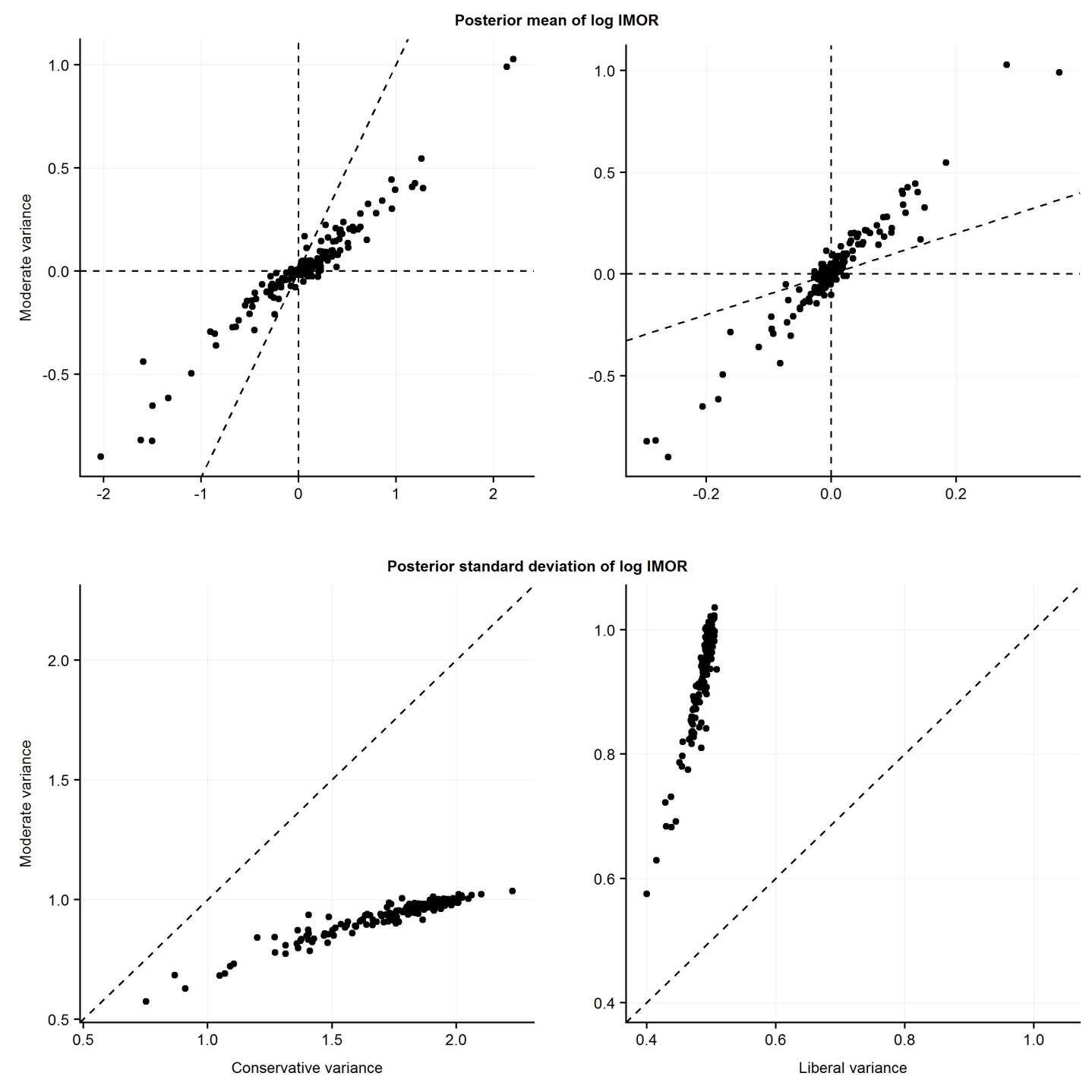
**

**Figure S7(d).** Scatterplots on posterior mean of log IMORs (first row) and posterior standard deviation of log IMORs (second row) between moderate and alternative prior variances for log IMORs. Use of identical, intervention-specific, normal prior distribution on log IMORs under on average missing at random. In the first row, 1:1 diagonal line has been included in both plots. IMOR, informative missingness odds ratio.
